# Genetic architecture of epigenetic and neuronal ageing rates in human brain regions

**DOI:** 10.1038/ncomms15353

**Published:** 2017-05-18

**Authors:** Ake T. Lu, Eilis Hannon, Morgan E. Levine, Eileen M. Crimmins, Katie Lunnon, Jonathan Mill, Daniel H. Geschwind, Steve Horvath

**Affiliations:** 1 Department of Human Genetics, David Geffen School of Medicine, University of California Los Angeles, Los Angeles, California 90095, USA; 2 University of Exeter Medical School, University of Exeter, RILD Building, Barrack Road, Exeter EX2 5DW, UK; 3 Center for Neurobehavioral Genetics, University of California Los Angeles, Los Angeles, California 90095, USA; 4 Davis School of Gerontology, University of Southern California, Ethel Percy Andrus Gerontology Center, 3715 McClintock Avenue, Los Angeles, California 90089-0191, USA; 5 Institute of Psychiatry, King's College London, London SE5 8AF, UK; 6 Neurogenetics Program, Department of Neurology, David Geffen School of Medicine, University of California Los Angeles, Los Angeles, California 90095, USA; 7 Center for Autism Research and Treatment, Semel Institute, David Geffen School of Medicine, University of California Los Angeles, Los Angeles, California 90095, USA; 8 Department of Biostatistics, School of Public Health, University of California Los Angeles, Los Angeles, California 90095, USA

## Abstract

Identifying genes regulating the pace of epigenetic ageing represents a new frontier in genome-wide association studies (GWASs). Here using 1,796 brain samples from 1,163 individuals, we carry out a GWAS of two DNA methylation-based biomarkers of brain age: the epigenetic ageing rate and estimated proportion of neurons. Locus 17q11.2 is significantly associated (*P*=4.5 × 10^−9^) with the ageing rate across five brain regions and harbours a *cis*-expression quantitative trait locus for *EFCAB5* (*P*=3.4 × 10^−20^). Locus 1p36.12 is significantly associated (*P*=2.2 × 10^−8^) with epigenetic ageing of the prefrontal cortex, independent of the proportion of neurons. Our GWAS of the proportion of neurons identified two genome-wide significant loci (10q26 and 12p13.31) and resulted in a gene set that overlaps significantly with sets found by GWAS of age-related macular degeneration (*P*=1.4 × 10^−12^), ulcerative colitis (*P*<1.0 × 10^−20^), type 2 diabetes (*P*=2.8 × 10^−13^), hip/waist circumference in men (*P*=1.1 × 10^−9^), schizophrenia (*P*=1.6 × 10^−9^), cognitive decline (*P*=5.3 × 10^−4^) and Parkinson's disease (*P*=8.6 × 10^−3^).

## Abstract (web summary)

Studies on the ‘epigenetic clock', a recently identified ageing biomarker, suggest that pathology might be linked to tissue-specific accelerated ageing. Here, the authors investigate ageing in the human brain and identify genetic loci associated with accelerated ageing in different brain regions.

It is projected that over the next 45 years, the number of older adults (ages 65 and older) in the United States will more than double—increasing from ∼46 million to over 98 million^1^. After age 65, the risk of developing a form of dementia increases exponentially^2^, highlighting the immediate need for therapeutics based on an improved understanding of the aetiology of normal cognitive ageing and neurodegenerative disease.

GWASs of dementias and cognitive functioning traits have had considerable success: 19 genetic loci have been found for Alzheimer's disease^3^, 24 loci for Parkinson's disease^4^ and 3 loci for general cognitive functioning^5^ at a genome-wide significance level (*P*<5 × 10^−8^). By contrast, relatively little is known about the genetic contributors to the underlying biological ageing processes in the brain. Although age is the major risk factor for neurodegenerative conditions, whether ageing and these disorders of ageing are part of a continuum or discrete entities remains a subject of substantial debate. One obstacle in answering this question is the lack of consensus regarding how to measure biological ageing. We recently developed a biomarker of ageing known as the epigenetic clock, which allows one to estimate the age (DNA methylation age (DNAm age)) of any human tissue or cell type (with the exception of sperm)^6^,^7^,^8^,^9^,^10^. DNAm age is calculated using the weighted average of DNA methylation levels at 353 CpG sites^6^. From this, one can define a measure of epigenetic age acceleration, by contrasting DNAm age with chronological age, such that a positive value implies the sample is biologically older than expected, whereas a negative value implies the sample is younger than expected. Epigenetic age acceleration in blood is predictive of mortality/longevity^11^,^12^,^13^,^14^,^15^ and has been linked to cognitive functioning^16^, Parkinson's disease^17^, Down syndrome^9^ and menopause^18^. In brain tissues, epigenetic age acceleration has been linked to Down syndrome, Huntington's disease (HD) and Alzheimer's disease^9^,^19^,^20^.

Epigenetic age acceleration differs across ethnic groups^21^ and is highly heritable (*h*^2^∼0.4)^6^,^11^,^20^. However, to date, only two genome-wide significant loci have been found to relate to epigenetic age acceleration: loci near genes *MLST8* and *DHX57* relate to age acceleration in the cerebellum (CRBLM)^22^. It is not yet known whether these or other genetic loci relate to the epigenetic age acceleration in other brain regions. Further, it is not known whether individuals who display signs of accelerated epigenetic ageing in CRBLM also exhibit accelerated epigenetic ageing in the prefrontal cortex (PFCTX) or other brain regions.

To address these questions, we study two distinct measures of brain ageing based on DNA methylation data: epigenetic age acceleration and the estimated proportion of neurons. We find genetic variants that accelerate brain ageing by ∼1 year. We use transcriptomic studies to prioritize genes that are located near genome-wide significant loci. The biological relevance of these findings is supported by our finding that both biomarkers of brain ageing relate to a host of age-related phenotypes according to GWAS results. Overall, this study elucidates the genetic architecture of epigenetic and neuronal ageing rates in human brain regions.

## Results

### Study overview

Our meta-analysis involved DNA methylation data and corresponding single-nucleotide polymorphism (SNP) data from seven different studies, totalling *n*=1,796 postmortem brain samples from 1,163 individuals of European ancestry (Table 1). Samples came from the PFCTX region (36.6%), including dorsolateral prefrontal cortex (DLPFX), CRBLM (31%), frontal cortex (FCTX, 18.6%), pons (PONS, 7%) and temporal cortex (TCTX, 7%). For five studies, we also had access to complementary transcriptional data collected from the same individuals (Table 1; Supplementary Tables 1 and 2a,b). The chronological age at death ranged from 1 to 108 years, with mean ages of death for the seven studies ranging from 44.3 years (study 3) to 89.3 years (study 7). About half (54%) of the individuals were female. Studies 2 and 4 involved neurologically normal individuals, whereas the remaining studies included individuals suffering from Alzheimer's disease, schizophrenia or other disorders (Supplementary Note 1). The individual studies differed greatly in terms of sample size ranging from *n*=36 (study 4) to *n*=302 (study 6).

**Figure 1:**
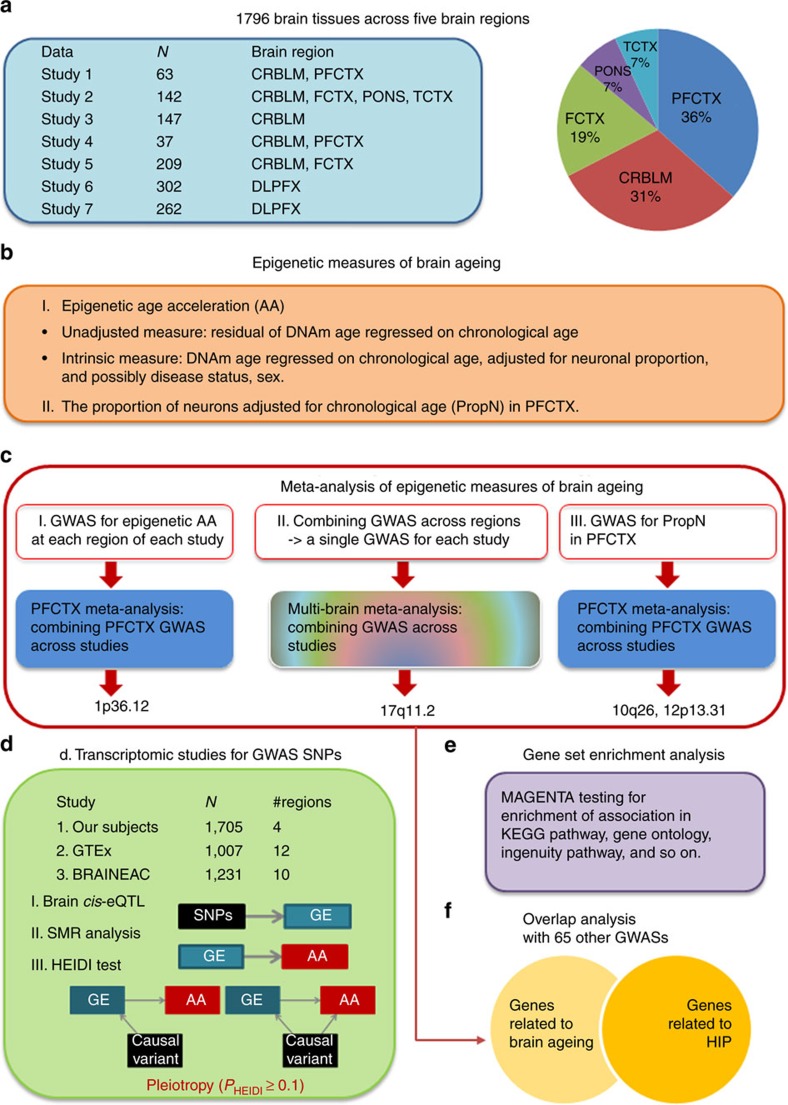
Overview of the analysis that characterized genetic factors underlying epigenetic measures of brain ageing. (**a**) The study involved SNP data and DNA methylation data from 1,796 brain tissue samples across multiple brain regions: cerebellum (CRBLM), frontal cortex (FCTX), pons (PONS), (dorsal lateral) prefrontal cortex (DLPFX/PFCTX) and temporal cortex (TCTX). (**b**) Our GWASs involved two DNA methylation-based traits of brain ageing: epigenetic age acceleration (based on the epigenetic clock) and the proportion of neurons (estimated using the CETS algorithm). The (cell-)intrinsic measure of epigenetic age acceleration in brain tissue is defined to be independent of the proportion of neurons. (**c**) To combine the GWAS results of individual brain regions across seven different studies, we used meta-analysis. (**d**) To prioritize genes near genome-wide significant loci, we used *cis*-eQTL studies and Mendelian randomization analyses based on summary test statistics. (**e**) To identify biological pathways underlying epigenetic measures of brain ageing, we used gene set enrichment analysis. (**f**) To demonstrate that SNPs associated with brain ageing are often associated with other complex phenotypes, we used a gene overlap analysis with published GWAS results. AA, age acceleration; GE, gene expression; HIP, hip circumference; SMR, summary data-based Mendelian randomization.

A graphical overview of our study samples and statistical procedures is presented in Fig. 1. Our GWAS aimed to elucidate the genetic determinants of two distinct biomarkers of brain ageing: (i) DNAm age based on 353 CpGs from the epigenetic clock method, and (ii) the proportion of neurons estimated using the CETS algorithm^23^. Each epigenetic biomarker was adjusted for chronological age and other potential confounders. The age-adjusted biomarkers were used as quantitative traits in a GWAS. GWAS results from different brain regions and studies were combined using meta-analysis. Transcriptomic data were used to prioritize candidate genes next to GWAS hits. Our overlap analysis investigates whether SNPs that relate to brain ageing exhibit a pleiotropic effect on other complex traits including neurodegenerative disease, body fat distribution, metabolic phenotypes, inflammatory disease, longevity and neuropsychiatric disorders.

### Epigenetic clock analysis

Across all seven studies, DNAm age was highly correlated with chronological age (0.61≤*r*≤0.99, Supplementary Fig. 1), which validated the high accuracy of the epigenetic clock. As expected, the largest age correlations (0.87≤*r*≤0.99) could be observed for the studies with the broadest age range (studies 2–5, Table 1). We defined a measure of epigenetic age acceleration as the residual resulting from regressing DNAm age on chronological age. Thus, a positive (negative) value of epigenetic age acceleration indicates that the brain region is older (younger) than expected based chronological age.

### Age acceleration negatively correlated with neurons

Previous work has suggested that the proportion of neurons (relative to glial cells) increases with age in many brain regions, at least in older individuals^23^. We confirmed this finding in our study, showing that the proportion of neurons, as estimated on the basis of DNA methylation data using the CETS algorithm^23^, tends to exhibit a positive correlation with chronological age in the CRBLM, FCTX and PFCTX (Supplementary Fig. 2). On the basis of this, we also examined whether the proportion of neurons varies as a function of age acceleration. Strikingly, we found a highly significant negative correlation between epigenetic age acceleration and the proportion of neurons in the PFCTX (for example, *r*=−0.33, *P*=4.0 × 10^−9^ in study 6 and *r*=−0.37, *P*=6.4 × 10^−10^ in study 7, Supplementary Fig. 3). Interestingly, Alzheimer's disease status was significantly associated with epigenetic age acceleration in PFCTX, but only after adjusting for the proportion of neurons (*P*=5.7 × 10^−3^, Supplementary Table 3). To remove the effect of potential confounders on our measure of age acceleration, we defined an intrinsic measure of age acceleration by regressing the unadjusted measure on the proportion of neurons, disease status and sex (Supplementary Table 4). By definition, the intrinsic measure of age acceleration in the brain is not correlated (*r*=0) with chronological age, the proportion of neurons, sex or disease status. A positive/negative value age acceleration indicates that the brain sample is older/younger than expected.

### Age acceleration is only weakly preserved across regions

To study whether epigenetic age acceleration in one brain region correlates with that of another brain region, we focused on individuals for whom multiple brain regions were available. Higher correlations were found between epigenetic age acceleration measures of various cortical regions, whereas lower correlations were observed between cortical regions and the CRBLM (median correlation=0.39, ranging from −0.04 to 0.52, Supplementary Fig. 4). The relatively low correlation between region-specific measures of age acceleration suggests that SNPs that relate to the epigenetic age acceleration of the PFCTX might be different from SNPs that relate to the epigenetic age acceleration of the CRBLM and vice versa. However, differences in sample size contributed to differences in statistical power when it came to detecting significant correlations between two brain regions. For example, our study had only a power of 38% (at a significance level of 0.01) to detect a moderate correlation (*r*=0.3) between the CRBLM and the PFCTX (*N*=57 pairs). However, we had a statistical power of 96% to detect a significant correlation of *r*=0.3 between the CRBLM and FCTX (*N*=201), and a power of 75% for other pairs of brain regions (*N*=112).

### 17q11 locus found by multi-brain region meta-analysis

**Figure 2:**
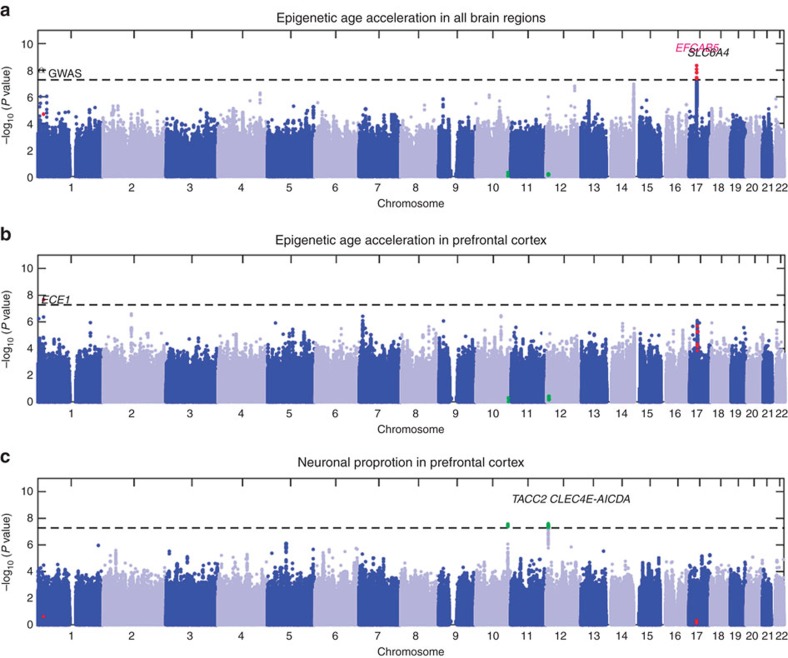
Manhattan plots of genome-wide meta-analysis. Manhattan plot for the meta-analysis GWAS *P* values of (**a**) epigenetic age acceleration across multiple brain regions (cerebellum, frontal cortex, pons and prefrontal cortex), (**b**) epigenetic age acceleration in the prefrontal cortex (PFCTX) and (**c**) an age-adjusted measure of the proportion of neurons. Each panels depicts eight SNPs (coloured in red) that are significantly (*P*<5.0 × 10^−8^) associated with epigenetic age acceleration in either (**a**) all five brain regions or in (**b**) PFCTX. Further, each panel highlights 13 SNPs (coloured in green), which are significantly associated with the proportion of neurons (*P*<5.0 × 10^−8^) in PFCTX (**c**).

**Figure 3:**
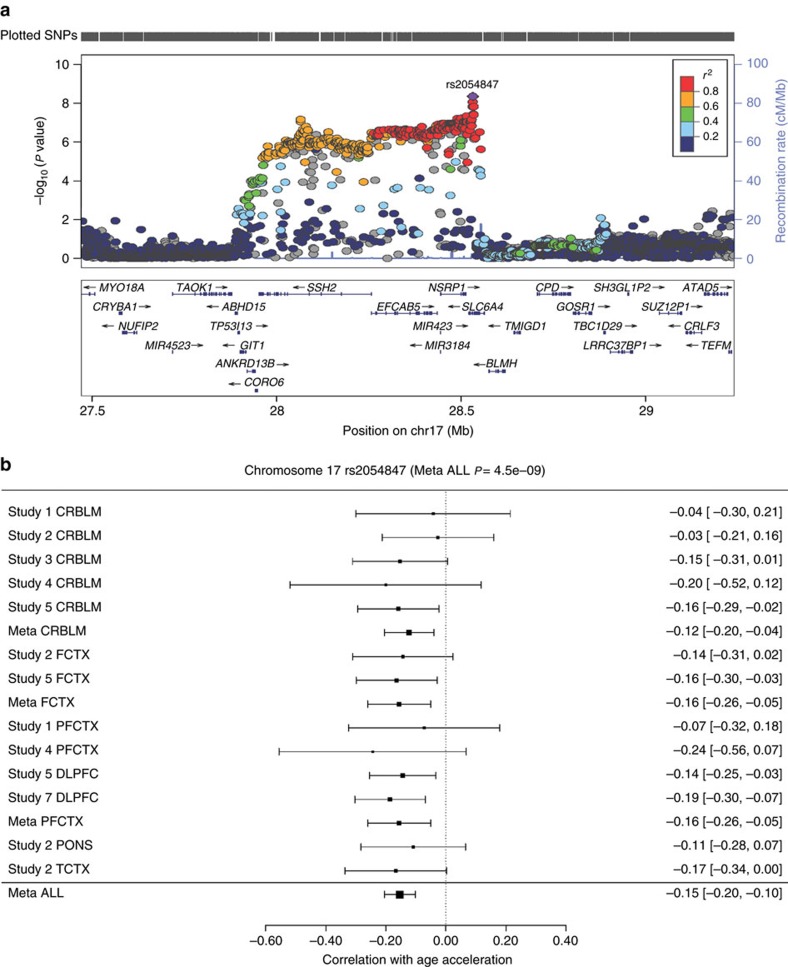
Detailed analysis of locus 17q11. (**a**) Regional association plot of locus 17q11.2 associated with epigenetic age acceleration. The *y* axis depicts log-transformed meta-analysis *P* values across studies 1–7. The colours visualize linkage disequilibrium (LD) 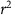 between rs2054847 (coloured in purple) and neighbouring SNPs. (**b**) The meta-analysis ‘forest' plot displays the GWAS results across all brain regions of GWASs 1–7. We display study index, brain region, width of 95% confidence interval (CI) for correlation coefficient estimate and correlation [95% CI], with respect to the minor allele A. The results from cerebellum (CRBLM), frontal cortex (FCTX) and prefrontal cortex (PFCTX) were combined into single estimates, referred to as Meta CRBLM, Meta FCTX and Meta PFCTX, respectively. The estimate Meta ALL combined each single estimate of each GWAS (1–7, total *N*=1,796) via fix-effect models weighted by inverse variance. It indicates that rs2054847 is associated with epigenetic age acceleration across five brain regions at a genome-wide significant *P*=4.5 × 10^−9^.

We performed GWAS for each brain region in each study, resulting in 13 separate GWAS results (Supplementary Table 2). The individual GWAS results were combined using two distinct meta-analyses: the first ‘multi-brain region' meta-analysis combined the GWAS across all brain regions and studies, the second ‘PFCTX' meta-analysis only combined GWAS results from the PFCTX (Methods). A Manhattan plot for the meta-analysis GWAS of intrinsic epigenetic age acceleration in the brain (Fig. 2a) reveals that the most significant locus occurs in 17q11.2. None of the significant GWAS results for age acceleration co-locate (within 1 Mb) with any of the 353 CpGs that make up the epigenetic clock. In particular, the most significant ‘leading' SNP in 17q11.2 SNP (rs4054847 at 28,532,013 bp) is 1.16 Mb away from the closest clock CpG cg06144905 (at 27,369,780 bp). Our multi-region meta-analysis revealed that seven SNPs in the 17q11.2 locus are associated with epigenetic age acceleration in the brain at a genome-wide significance level (*P*<5.0 × 10^−8^, Table 2). The leading SNP rs2054847 (*P*=4.5 × 10^−9^) is located in the serotonin transporter gene *SLC6A4* (Fig. 3a) but our *cis*-expression quantitative trait locus (*cis*-eQTL) studies (described below) provide no evidence that the SNP modulates the expression levels of this gene. The minor allele of the leading SNP is strongly associated with decreased (negative) epigenetic age acceleration across the five brain regions (Fig. 3b): each copy of the minor allele decreases the epigenetic age by 1.4 years in FCTX, ∼1 year across PONS and TCTX and 0.8 years across PFCTX and CRBLM. The association result for rs2054847 was highly conserved across individual GWASs, as reflected by a vanishing meta-analysis heterogeneity measure (*I*^2^=0%). The strong association signal in 17q11.2 is also supported by an additional 210 neighbouring SNPs that meet a suggestive significance level of *P*<5 × 10^−7^ in the linkage disequilibrium (LD; *r*^2^>0.6) region spanning 490 kb around rs2054847 (Fig. 3a). Although the genomic region surrounding rs2054847 contains many suggestive SNPs and genes, it harbours only one causal locus for epigenetic age acceleration according to the GCTA conditional analysis^24^ (Supplementary Fig. 6; Methods).

Our chromatin state analysis based on data from the Roadmap Epigenomics Consortium^25^,^26^ demonstrated that a SNP in the 17q11.2 locus (rs1128156, GWAS *P*=9.0 × 10^−8^ for age acceleration) is in an actively transcribed region in 126 out of 127 cell lines (Supplementary Fig. 7a). By contrast, the leading SNP, rs2054847, which is in high LD *r*^2^=0.89 with rs1128156, is located in an actively transcribed region for only a few cell lines (Supplementary Fig. 7b).

### 1p36 locus found by PFCTX meta-analysis

We also carried out a second meta-analysis using only PFCTX GWAS results from studies 1, 4, 6 and 7. The resulting Manhattan plot can be found in Fig. 2b. This PFCTX meta-analysis GWAS of age acceleration identified an INDEL (deletion variant) marker, rs11296960, in 1p36.12 (*P*=2.2 × 10^−8^, Table 2; Supplementary Fig. 8). Each copy of the minor allele of rs11296960 increases the epigenetic age of the PFCTX by 1.07 years (resulting in a correlation of *r*=0.21 between the minor allele count and epigenetic age acceleration). The INDEL marker, rs11296960, is located within the endothelin-converting enzyme 1 gene (*ECE1*), previously implicated in Alzheimer's disease due to its perceived effect on amyloid-beta peptides levels^27^,^28^. The association signal of rs11296960 is supported by six neighbouring SNPs (located in the *ECE1* gene), which have a suggestive association with epigenetic age acceleration (4.4 × 10^−7^<*P*<9.8 × 10^−5^, Supplementary Fig. 8a). However, the 1p36.12 locus exhibits significant heterogeneity across studies (*I*^2^=85%, *P*=0.002), which resulted from a high correlation coefficient from the smallest study (*n*=36 in study 4, Supplementary Fig. 8b). As part of a sensitivity analysis of study 4, we also conducted a robust correlation test (biweight midcorrelation^29^) that corroborated our original results (Supplementary Fig. 9).

A chromatin state analysis found that INDEL rs11296960 is located in a region that either actively involves or enhances gene regulation in 124 out of 127 cell lines, including 8 brain cell lines (Supplementary Fig. 7c).

Different from its effect in the PFCTX, INDEL rs11296960 is not associated with epigenetic age acceleration in the CRBLM (Supplementary Fig. 4a,h). Similarly, the two SNPs identified in our previous GWAS of epigenetic age acceleration in CRBLM^22^ are not associated with epigenetic age acceleration in PFCTX (Supplementary Table 5). However, these SNPs exhibit a suggestive association with age acceleration in the PONS, which is a related subcortical brain stem region highly interconnected with the CRBLM (0.026≤*P*≤0.09 in study 2).

### *cis-*eQTL studies of significant loci

**Figure 4:**
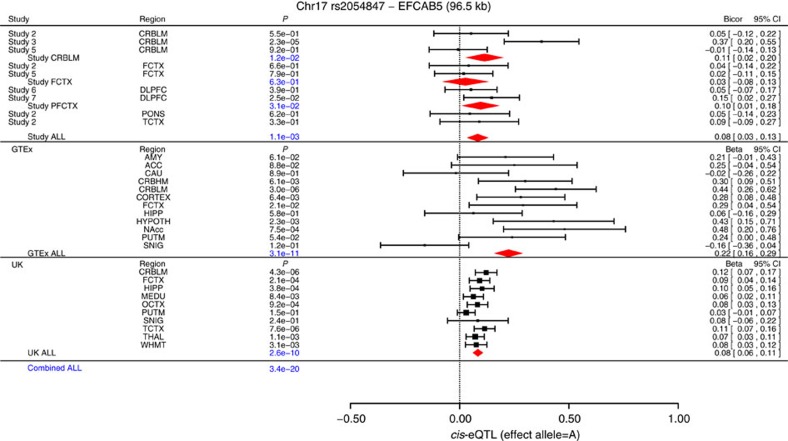
*cis*-eQTL study across 19 brain regions for the leading SNP in 17q11. The meta-analysis forest plot displays the significant *cis*-eQTL results for the leading SNP rs2054847 and expression levels of gene *EFCAB5*, by combining three panels of study results (*N*=3,943 brain tissues across 19 regions). We display study name, brain region and test statistics including *P* value, 95% confidence interval (CI) and effect size [95% CI]. The top panel reports a robust correlation coefficient (biweight midcorrelation, bicor). The remaining panels report the beta coefficient value (slopes) of linear regression models between a test allele and gene expression levels. The effect sizes are with respect to minor allele counts. The top panel reports *cis*-eQTL findings for 1,705 brain tissues across five brain regions from the individuals of our GWAS. Meta-analysis was used to combine individual results from CRBLM into a single estimate, Meta CRBLM. Similarly, we defined meta-analysis results for the frontal cortex (Meta FCTX) and prefrontal cortex (Meta PFCTX). Fixed-effects meta-analysis was used to combine Meta CRBLM, Meta FCTX and Meta PFCTX P values into the meta-analysis *P* value (Study ALL). The middle panel reports the results from the 1,007 brain tissues across 12 regions from the GTEx project. The fixed-effect model was used to combine GTEx *P* values into an overall *P* value denoted GTEx ALL. The lower panel reports the *cis*-eQTL results evaluated in up to 1,231 brain tissue samples across 10 regions, from the BRAINEAC database. The average across all available regions in the BRAINEAC data based is presented in (UK ALL). The Combined ALL P value was calculated by combining the Study ALL, GTEx ALL and UK ALL values using Stouffer's *Z* score approach. All the *cis*-eQTL models assumed used an additive allele coding of the SNP. ACC, anterior cingulate cortex; AMY, amygdala; CAU, caudate basal ganglia; CORTEX, cortex; CRBHM, cerebellar hemisphere; CRBLM, cerebellum; DLPFX, dorsolateral prefrontal cortex; FCTX, frontal cortex; HIPP, hippocampus; HYPOTH, hypothalamus; MEDU, medulla; NAcc, nucleus accumbens; OCTX, occipital cortex; PONS, pons; PUTM, putamen; SNIG, substantia nigra; TCTX, temporal cortex; THAL, thalamus; WHMT, intralobular white matter.

We performed a *ci*s-eQTL analysis to identify the functional consequences and regulatory targets of our GWAS hits within an interval of ±1 Mb (Fig. 1d; Methods). We analysed brain expression data (*n*=3,943 brain samples from 19 brain regions) by leveraging the following three large data sets: (1) transcriptomic data on the individuals from our GWAS (*n*=1,705 samples from 4 brain regions), (2) Genotype-Tissue Expression project (GTEx, see URL)^30^ (*n*=1,007 samples, across 12 brain regions, from 449 individuals, most of whom were neurologically normal and of European Ancestry, Supplementary Table 6) and (3) the Brain eQTL Almanac (BRAINEAC, see URL)^31^ (*n*=1,231 samples from 10 brain regions of 134 neurologically normal individuals of European ancestry). We combined the eQTL *P* values across the three studies using Stouffer's meta-analysis method. The 1p36.12 locus, which was implicated in our PFCTX meta-analysis, did not exhibit any *cis*-eQTL after correcting for multiple comparisons. However, locus 17q11.2, which was identified by our multi-region meta-analysis, implicated four gene transcripts that are significantly correlated with the leading SNP, rs2054847 (Fig. 4; Supplementary Fig. 10; Supplementary Table 7). The minor allele of rs2054847, which is associated with decreased epigenetic age acceleration in brain, is positively correlated with the expression levels of *EFCAB5* (EF-hand calcium-binding domain 5) in 12 different brain regions (Meta *P*=3.4 × 10^−20^, Fig. 4) and even in non-neural tissues, such as colon, nerve, skin and thyroid (Supplementary Table 8). A less significant *cis*-effect of SNP rs2054847 can be observed for the expression levels of three other neighbouring genes: *GOSR1* (*P*=5.5 × 10^−12^, Supplementary Fig. 10a), *CRLF3* (*P*=2.1 × 10^−6^), and *BLMH* (*P*=6.3 × 10^−6^, Supplementary Fig. 10b,c). Each of the four putative *cis*-acting genes has at least a marginally significant correlation with chronological age (meta-analysis *P*<0.05), but the most significant age correlations can be observed for *CRLF3* (meta-analysis *P*=1.7 × 10^−5^, Supplementary Figs 11 and 12).

### *EFCAB5* is the most striking gene in 17q11

If the expression level of a gene is influenced by a genetic variant, also known as an expression QTL, then there will be differences in gene expression levels among individuals carrying different genotypes of the genetic variant. Then, if the expression level of the gene has an effect on epigenetic age acceleration, the genetic variant will also show an effect on epigenetic age acceleration. This approach is very similar to the concept of a Mendelian randomization (MR) analysis, where a genetic variant (for example, a SNP) is used to test for the causative effect of an exposure (for example, gene expression) on an outcome (for example, epigenetic age acceleration), yielding a measure of the causative effect, irrespective of potential confounders. Therefore, one can, in principle, use MR analysis to search for the most functionally relevant genes at the loci identified in a GWAS for a complex traits^32^. However, MR analysis based on a single genetic variant is unable to distinguish the causal model (SNP→expression→age acceleration) from the alternative causal scenario of pleiotropy (expression←SNP→age acceleration, Supplementary Fig. 13a,b)^32^. To err on the side of caution, we refer to a significant MR test between the expression trait and epigenetic age acceleration as ‘pleiotropic association' even though it could indicate a causal effect of gene expression on age acceleration.

To detect the effect of a gene expression on epigenetic age acceleration using a two-stage least-squares MR approach probably requires a large sample size (possibly thousands of individuals), whereas we only had access to a moderate sample size of individual-level data (that is, individuals for whom DNA methylation, SNP and gene expression data were measured at the same time). Nevertheless, we were able to leverage summary-level data (test statistics) from large-scale GWAS and eQTL studies in the public domain, and apply the summary data-based Mendelian randomization (SMR) method to identify genes whose expression levels are associated with epigenetic age acceleration^32^. The SMR analysis combined our GWAS results of epigenetic age acceleration with *cis*-eQTL GWAS results from (1) our study data, (2) GTEx and (3) BRAINEAC. The SMR analysis of the 17q11.2 region suggests a pleiotropic association between epigenetic age acceleration and the expression levels of four genes: *EFCAB5*, *GOSR1*, *CRLF3* and *BLMH*^32^ (Fig. 1d). The EF-hand gene *EFCAB5* has the strongest pleiotropic association with epigenetic age acceleration (Table 3; Supplementary Table 9). The pleiotropic association between *EFCAB5* and epigenetic age acceleration is due to a single causal variant in 17q11.2 according to the insignificant HEIDI test (Table 3; Supplementary Fig. 13). The minor allele ‘A' of the leading SNP rs2054847 is associated with higher expression levels of *EFCAB5* in multiple brain regions, which suggests that elevated expression levels are associated with delayed brain ageing. Using individual-level data, we find a striking negative correlation between *EFCAB5* expression levels and epigenetic age acceleration in the CRBLM (Meta *P*=1.7 × 10^−10^, Table 3), FCTX (Meta *P*=7.8 × 10^−6^), PFCTX (*P*=9.2 × 10^−3^) and TCTX (*P*=2.9 × 10^−4^). Overall, we find a highly significant association between *EFCAB5* expression and epigenetic age acceleration in brain across all studies (*P*=1.2 × 10^−16^, Table 3).

We cannot rule out that the genome-wide significant SNPs directly affect epigenetic ageing rates, which subsequently alter gene transcript levels. An SMR analysis that reverses the roles of gene transcripts and epigenetic ageing rates indicates that the rates might have a direct causal effect on *EFCAB5* expression levels in the CRBLM (SMR *Z*=−3.66 and *P*=2.5 × 10^−4^) and in the PFCTX (SMR *Z*=−2.03 and *P*=4.3 × 10^−2^).

### Only suggestive enrichment for pathways

To learn more about the biological processes that may underlie epigenetic age acceleration in the brain, we performed pathway analysis using MAGENTA^33^ for two sets of SNPs—those resulting from our meta-analysis of all regions and those from our meta-analysis of PFCTX (Methods; Fig. 1e). While the *P* values are not significant after adjusting for multiple comparisons, we find suggestive evidence that genes that relate to epigenetic age acceleration of the PFCTX play a role in DNA damage, GTPase inhibitor activity and neuroactive ligand receptor interactions (Supplementary Table 10; Supplementary Data 1). Similarly, genes that relate to epigenetic age acceleration across multiple brain regions are enriched with genes that play a role in mitogen-activated protein kinase signalling (Supplementary Table 10; Supplementary Data 1).

### Significant overlap with GWAS results of other phenotypes

To rank genes (as opposed to individual SNPs) based on our GWAS of age acceleration, we used the MAGENTA software to assign an overall *P* value per gene based on multiple underlying SNPs. Towards this end, MAGENTA assigns a *P* value to each gene by adjusting the most significant SNP association *P* value (within the gene boundary ±50 kb) for gene size, number of SNPs in LD per gene and other potential confounders^33^.

Similarly, we ranked the results from 65 GWAS of a broad spectrum of phenotypes such as neurodegenerative diseases^3^,^34^,^35^,^36^, neuropsychiatric disorders^37^, body fat distribution^38^, metabolic phenotypes^39^, inflammatory disease^40^ and longevity (Fig. 1f; Methods; Supplementary Note 2). We then examined the overlap between the top 2.5% most significant genes (roughly 500 genes ranked by the MAGENTA *P* value) for epigenetic age acceleration and an analogous set of genes found by GWAS of other phenotypes. According to the overlap analysis (Table 4), genes associated with intrinsic epigenetic age acceleration in the PFCTX have been implicated in cognitive decline (*P*=1.2 × 10^−3^), dementia (*P*=1.2 × 10^−3^), Alzheimer's disease (*P*=4.9 × 10^−3^) and hip/waist circumference (adjusted for body mass index (BMI)) in a sex-specific manner: the most significant overlap can be observed for males. When studying the overlap between sets of genes based on a MAGENTA threshold of 15% (roughly 2,800 significant genes), we found that genes related to epigenetic age acceleration in all brain regions (*P*=7.4 × 10^−5^) and in PFCTX (*P*=3 × 10^−3^) overlap with genes that are known to modify the age of onset of HD according to a recent large-scale GWAS^35^. The latter results are consistent with our recent finding that HD is associated with epigenetic age acceleration in human brain tissue^19^.

Individual genes that relate both to brain epigenetic age acceleration and to at least one test trait according to our overlap analysis can be found in Supplementary Data 2 and 3.

### GWAS of the proportion of neurons in PFCTX

In the following, we describe the results for our second measure of brain ageing: an age-adjusted measure of the proportion of neurons, which was estimated using DNA methylation data (Methods).

Our meta-analysis involving 600 PFCTX tissues (from studies 4, 6 and 7, Fig. 1c; Methods) identified two significant loci (Fig. 2c): 10q26 (3 SNPs within gene *TACC2*) and 12p13.31 (10 SNPs near genes *CLEC4E-AICDA*, Supplementary Table 12; Supplementary Fig. 14). As described above, we used MAGENTA to define sets of genes that relate to the proportion of neurons according to our GWAS analysis. According to our GWAS-based overlap analysis, the age-adjusted proportion of neurons relates significantly to 21 traits (Table 4; Supplementary Table 13; Supplementary Data 4) including age-related macular degeneration (*P*=1.4 × 10^−12^), schizophrenia (*P*=1.6 × 10^−9^), cognitive decline (5.3 × 10^−4^), Parkinson's disease (*P*=8.6 × 10^−3^) and all three subtypes of inflammatory bowel disease (*P*≤6.0 × 10^−9^ including ulcerative colitis *P*<1.0 × 10^−20^), type 2 diabetes (*P*=2.8 × 10^−13^ in individuals of European ancestry), and various measures of body fat distribution, with stronger effects found for males (such as hip and waist circumference adjusted for BMI in males of European ancestry *P*=1.1 × 10^−9^, Table 4).

### Unclear causal relationship between adiposity and brain age

It is striking that, according to our overlap analysis, both of our DNA methylation-based biomarkers of brain ageing (epigenetic age acceleration and the proportion of neurons) relate to measures of adiposity (hip and waist circumference adjusted for BMI) in a sex-specific manner. To assess whether epigenetic brain ageing is a downstream causal consequence of adiposity, or whether pleiotropy links adiposity with brain ageing, we used a powerful variant of MR analysis (MR-Egger regression^41^) that effectively combines the information of multiple SNPs (Methods). Towards this end, we used multiple genome-wide significant SNPs for body fat distribution traits that were found in a GWAS of 93,965 males of European ancestry from the GIANT consortium^38^ (Methods). We considered several measures of adiposity including waist and hip circumference (both adjusted and unadjusted for BMI). However, MR-Egger regression analysis did not reveal significant evidence for a causal effect of adiposity on our biomarkers of brain ageing or vice versa (Supplementary Table 14).

As a secondary analysis, we also carried out a polygenic risk score analysis for the measures of adiposity (waist and hip circumference adjusted for BMI), which were constructed using the data from 93,965 males of European ancestry in the GIANT consortium (Supplementary Tables 15 and 16). The polygenic risk scores applied to our individual-level data resulted in genetic estimates of waist/hip circumference, which exhibited insignificant correlations with our epigenetic biomarkers (Supplementary Table 15).

## Discussion

Our study elucidates the genetic underpinnings of two DNA methylation-based biomarkers of brain ageing: the first, epigenetic age acceleration based on the epigenetic clock, is associated with two loci (17q11.2 and 1p36.12); the second, proportion of neurons based on the CETS algorithm, is associated with two other loci (10q26 and 12p13.31). Our transcriptomic studies allowed us to prioritize genes that are located near these genome-wide significant loci. Interestingly, Alzheimer's disease is associated with an increased epigenetic age acceleration of the PFCTX after adjusting for the proportion of neurons. This is consistent with our previous work showing that epigenetic age acceleration in PFCTX both relates to and shares a genetic correlation with Alzheimer's disease-related neuropathology^20^. By definition, our intrinsic measure of age acceleration is not confounded by the proportion of neurons, chronological age, sex or disease status.

Both biomarkers of brain ageing are associated with a host of complex phenotypes according to our GWAS-based overlap analysis. The genetic overlap between neurodegeneration and epigenetic age acceleration is evidenced by our results, showing that gene sets identified by our GWAS of epigenetic ageing in the PFCTX were significantly enriched with genes associated with cognitive decline, dementia, Alzheimer's disease and age of HD onset.

Locus 17q11.2 is particularly interesting since it is associated with epigenetic age acceleration across multiple brain regions. To study the biological mechanism of the leading GWAS SNP, rs2054847, we carried out *cis*-eQTL studies and MR studies. Our *cis*-eQTL study based on individual-level data shows that rs2054847 is associated with the expression levels of multiple genes (*EFCAB5*, *GOSR1*, *CRLF3* and *BLMH*) in multiple brain regions except for PONS. But differences in sample sizes per brain region contribute to differences in statistical power when it came to detecting SNPs for age acceleration and corresponding expression QTLs. We had a relatively low power of 64% (at a significance level of 0.05) to detect a weak correlation of 0.2 between a SNP and a neighbouring gene transcript in the PONS (*N*=134) compared to a high power of 93% in PFCTX (*N*≥288 in two studies). To overcome our limited sample size in individual-level data, we used summary-level data from published eQTL studies to show that *EFCAB5* correlates with rs2054847 in 12 brain regions and in non-neural tissues such as colon, nerve, skin and thyroid. Using individual-level data, we found that *EFCAB5* expression levels correlate positively with chronological age but negatively with epigenetic age acceleration in several brain regions. *EFCAB5* is an intriguing gene in the context of brain ageing because it is known to play a role in brain-related processes such as Ca^2+^ signalling, synaptogenesis, dendritic arborization and cell survival^42^.

We demonstrate that SNPs associated with epigenetic brain ageing in one brain region are typically different from those affecting ageing in another brain region. In particular, the CRBLM is distinct from other regions—an observation that is not surprising given its relative protection from most disorders associated with ageing and its slow epigenetic ageing rate^8^. This probably explains why the two SNPs identified in our previous GWAS of epigenetic age acceleration in CRBLM^22^ are not associated with epigenetic age acceleration in PFCTX.

We identified an INDEL variant rs11296960 near *ECE1* in 1p36.12, which relates to epigenetic age acceleration in PFCTX, but not in CRBLM. The INDEL variant is located in an active chromosomal region for gene regulation in brain and other cell lines. It has been suggested that *ECE1* acts as an Aβ-degrading enzyme in the brain, and that decreased presence of *ECE1* is associated with reduced Aβ clearance and increased plaque deposition^27^,^28^.

Although neuronal loss has been observed with ageing and dementia, we found that the actual proportion of neurons, relative to glia, is positively correlated with chronological age in several brain regions. Epigenetic age acceleration has a strong negative correlation with the proportion of neurons (on average *r*=−0.35), but these biomarkers only exhibited a vanishing genetic correlation (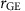
=0.005 according to the GCTA software^43^,^44^) with each other, which probably reflects the low heritability of the proportion of neurons (*h*^2^=7.2%) or the relatively low sample size (*n*<1,000). At least 5,900 samples are needed to reach a statistical power of 80% for detecting a heritability of 10% at a 0.05 significance level according to a GCTA-GREML power analysis^44^.

Our overlap analysis further suggests that gene sets identified in the GWAS for epigenetic ageing in PFCTX and those identified in the GWAS for proportion of neurons relate to fat distribution traits especially in males. We also find significant genetic overlap between the proportion of neurons and type II diabetes. This is particularly intriguing given the rich literature linking obesity and metabolic outcomes to cognitive functioning. For instance, obesity is associated with earlier onset of Alzheimer's disease^45^ and has been linked to cognitive decline and dementia^46^,^47^,^48^,^49^. However, our MR analysis (MR-Egger regression) did not reveal significant evidence for a causal effect of adiposity on our brain ageing measures. While these results point towards biological pleiotropy between age acceleration and measures of adiposity, additional studies will be needed to arrive at definitive results regarding the causal relationships between these complex traits.

## Methods

### Data sets

An overview of our data sets is presented in Table 1. Additional details can be found in Supplementary Tables 1 and 2, and Supplementary Note 1. All studies involved DNA methylation and SNP data measured from the same individuals. Furthermore, gene expression data (microarray or RNA sequencing) were available for all studies except studies 1 and 4. Our meta-analysis was approved by the ethics review board at UCLA (IRB#15-001479 and IRB#14-000061).

### Code availability

The measures of DNAm age are implemented in our freely available software (https://dnamage.genetics.ucla.edu).

### Estimation of neuronal proportions in brain tissues

The CETS R package^23^ was used to estimate the proportion of neurons based on DNA methylation data. We independently confirmed the high accuracy of the CETS algorithm by applying it on sorted neurons, which led to estimates of the proportion of neurons in excess of 0.99.

### Heritability estimation based on GCTA

The REML and bi-REML procedures of the GCTA software^44^,^50^ were used to estimate the heritability of and genetic correlations between epigenetic age acceleration and proportion of neurons, respectively. Towards this end, we applied the GCTA analysis to a large Alzheimer's disease data set^51^,^52^,^53^,^54^ (studies 6 and 7, Supplementary Information). The analysis was performed on 8,185,912 genotyped or imputed markers that satisfied the following criteria: marker info measure<0.6 and minor allele frequency (MAF)≥0.02. Both REML and bi-REML models were adjusted for sex, study set, disease status and ten principal components estimated from identity-by-state relationships.

### Conditional analysis

To test whether multiple independent causal variants are located in 17q11.2, we used the GCTA conditional analysis based on GWAS summary statistics^24^. The association analysis conditioned on the leading (most significant) SNP, rs2054847. The reference panel for inferring the LD pattern was based on the 1000 genome individuals (released in December 2013) with European ancestry (*N*=379).

### GWAS analysis for epigenetic age acceleration

SNP quality was assessed by estimating MAF, Hardy–Weinberg equilibrium and missingness rates across individuals (Supplementary Table 2). European ancestry of the individuals from study 2 was validated by the authors^55^, which led to the removal of two inconsistent individuals. The reported genetic ancestry of other study individuals was confirmed using principal component analysis plots or multidimensional scaling plots in conjunction with principal component analysis in PLINK^56^ and EIGENSTRAT^57^.

### Imputation

We used IMPUTE2 (refs 58, 59) with haplotypes phased by SHAPEIT^60^ to impute variants such as SNP and INDEL markers based on the latest 1000 Genome phase 3 haplotypes from 2,504 individuals (released in October 2014) with the exception of study 1 that was based on the haplotypes from 1,092 individuals (released in December 2013). The quality of imputed markers was assessed by the info measure>0.4 (in IMPUTE2). For association analysis, we regressed the age acceleration trait values on (1) estimated genotype dosage (counts of test alleles) or (2) expected genotype dosage, possibly adjusted for the first two principal components derived from identity-by-state relationships in case of admixed populations (Supplementary Table 2).

### Genome-wide meta-analysis of epigenetic age acceleration

Our meta-analysis was based on correlation coefficients or partial correlation coefficients (in case of principal component adjustment). Our multi-brain region GWAS used the UV-MA (meta-analysis of univariate results) approach^61^, which proceeded along the following steps. First, we performed GWAS for each brain region in each study, resulting in 13 separate GWAS results. Second, the GWAS results from multiple brain regions of the same study (that is, based on the same individuals) were combined using fixed-effects meta-analysis weighted by inverse variance. This study-specific meta-analysis resulted in a single meta-analysis GWAS for studies 1, 2, 4 and 5, respectively. However, the intra-individual correlations resulted in inflated meta-analysis *P* values for each study, which were corrected in the next step. Third, we applied genomic control corrections to the meta-analysis *P* value from each of the four studies. Fourth, we again used a fixed-effects meta-analysis to combine the seven GWAS results (from seven studies that involve independent individuals) into a final meta-analysis GWAS.

Our meta-analysis GWAS of the PFCTX combined the results from this brain region across studies 1, 4, 6 and 7 using a fixed-effects meta-analysis weighted by inverse variance. Our fixed-effects meta-analysis models were carried out with the software Metal^62^.

### Pre-processing steps of GWAS

Our GWAS focused on common SNP markers (MAF>5%). Further, we removed SNPs that exhibited substantial heterogeneity across studies according (Cochran Q *I*^2^ *P* value≤0.001). We used 6,935,762 (genotyped or imputed) SNPs present in at least four study sets for our multi-region region GWAS and 6,853,936 SNPs present in at least three study sets for our PFCTX GWAS. In a *post hoc* analysis, we evaluated the SNPs that were removed in our pre-processing/pre-filtering analysis. None of the removed SNPs exhibited genome-wide significant (*P*<5.0 × 10^−8^) associations with measures of epigenetic age acceleration or with disease status (for example, Alzheimer's disease) in the respective studies. Significant heterogeneity (Cochran Q) test results were largely due to study 4, which was a small (*N*=37) case–control study of schizophrenia. The small sample size of study 4 increased the heterogeneity of the meta-analysis results and prompted us to carry out a sensitivity analysis based on a robust correlation test (biweight midcorrelation^29^).

### GWAS analysis for the proportion of neurons in PFCTX

Our GWAS of the (age adjusted) proportion of neurons in PFCTX was based on a meta-analysis across studies 4, 6 and 7. Our phenotype (age-adjusted proportion of neurons) was defined as raw residual resulting from a linear regression model of the proportion of neurons (dependent variable) on chronological age at time of death (covariate). Our approaches for the GWAS of the proportion of neurons were identical to those for our GWAS of epigenetic age acceleration. The genomic inflation estimates were 1.08, 0.98 and 1.03 for GWAS studies 4, 6 and 7, respectively. Results were combined using fixed-effects meta-analysis whose genomic inflation factor was 1.03.

### LD analysis

Regional SNP association results were visualized with the software LocusZoom^63^. All LD estimates presented in this article were calculated using individuals of European ancestry from the 1000 genome reference panel (released November 2014).

### Chromatin state analysis of leading SNPs

For each genome-wide significant locus, we carried out a chromatin state analysis of the leading SNP using the UCSC genome browser. The *n*=127 diverse cell/tissue lines were profiled by the NIH RoadMap Epigenomics^26^ (*n*=111) and ENCODE projects^64^ (*n*=16). We used the 15-state chromatin model from ChromHMM, which is based on five histone modification marks^26^.

### *cis*-eQTL across brain regions

Our cis-eQTL study leveraged gene expression data from 3,943 brain samples, collected from 19 brain regions. The expression data came from three data sources. The first source involved our study individuals consisting of 1,705 brain tissue samples from four brain regions (Table 1; Supplementary Table 1). We arrived at this set of samples after excluding a couple of potential outliers, which were identified by an unsupervised hierarchical clustering analysis as detailed in Supplementary Figs 15–19. Studies 5 and 6 involved RNA sequencing array data sets in which we used the expression at gene levels for analysis. To protect against potential outliers, we ‘winsorized' the gene expression levels at a 5% threshold. The second source of expression data involved the latest eQTL results (V6) released from the GTEx project (see URL). We used the brain eQTL results evaluated in up to 1,007 brain samples from 12 brain regions collected from 449 individuals of mostly (>80%) European ancestry (Supplementary Table 6). The third source involved the cis-eQTL results evaluated in up to 1,231 brain samples across 10 brain regions from 134 neurologically normal individuals of European ancestry. We downloaded the gene expression of the study genes and their cis-SNPs from BRAINEAC (see URL).

In our study sets, we evaluated the correlation between SNPs and gene expression levels using a robust correlation estimate known as biweight midcorrelation, which is implemented in the ‘bicor' R function of the WGCNA R package^29^. To account for possible confounders, gene expression levels were adjusted for sex, batch effects and possibly the proportion of neurons (estimated using CETS). Our *cis*-eQTL involved all genes located within 1 Mb of the test SNP and preceded along the following three steps. In step (1), we identified (*cis*-acting) SNP–gene pairs by using gene expression data from our individual-level data, that is, nine gene expression data sets from five studies and four brain regions (Table 1). Genes that were significant at a Bonferroni corrected *P* value in any of the nine expression data sets were evaluated in subsequent assessments in the other two independent large-scale gene expression data sets (GTEx and the UK database), as described in Supplementary Table 7. We combined the multiple results for the CRBLM (studies 2, 3 and 5) into a single estimate using fixed-effects meta-analysis weighted by inverse variance (implemented in the ‘metafor' R package). The results can be found in Fig. 4 (Study CRBLM). Similarly, results of FCTX (PFCTX) from studies 2 and 5 (studies 6 and 7) were combined into a single estimate by fixed-effects meta-analysis in Fig. 4 (Study FCTX, PFCTX). All results were combined into a single estimate by the fixed-effect model, referred as to *Study ALL* in Fig. 4. In step (2), we reported the GTEx eQTL-released results including effect sizes (regression coefficients and s.e.'s) and associated *P* values, across a total of 12 brain regions: amygdala, anterior cingulate cortex, caudate basal ganglia, cerebellar hemisphere, CRBLM, cortex, FCTX, hippocampus, hypothalamus, nucleus accumbens, putamen and substantia nigra. We performed the same fixed-effects meta-analysis to combine the results across brain regions into a single estimate, referred as to GTEx ALL in Fig. 4. In step (3), we performed *cis*-eQTL analysis in ten brain regions including CRBLM, FCTX, hippocampus, medulla, occipital cortex, putamen, substantia nigra, TCTX, thalamus and intralobular white matter. We also performed the *cis*-eQTL analysis on the average across all available regions (downloaded from the database), yielding a single estimate for eQTL listed as UK ALL in Fig. 4. To summarize the eQTL results from the three sources of data by a single Z statistic, we applied Stouffer's meta-analysis *Z* statistic approach. This allowed us to combine three *P* values from Study ALL, GTEx ALL and UK ALL into a single *P* value referred as to Combined ALL in Fig. 4. The resulting Combined ALL *P* value should be considered as descriptive (as opposed to an inferential measure) since it ignores the dependence resulting from intra-individual correlations (due to multiple brain regions from the same individual in our study sets or in GTEx).

### SMR and HEIDI analysis

The summary data-based Mendelian randomization (SMR) analysis^32^ uses SNPs as instrumental variables to test for a direct association between gene expression levels and epigenetic age acceleration irrespective of potential confounders. The SMR approach is similar to the two sample MR approach by Burgess *et al*.^65^ Both approaches are attractive because (a) they allow the user to use summary-level GWAS data as opposed to individual-level data, (b) they can use GWAS data from different studies that greatly expands the precision of the estimates. The two sample method by Burgess is particularly attractive when it comes to carrying out a MR analysis based on multiple SNPs. In our study, we chose the SMR approach for three reasons. First, the SMR approach focuses on the identification of gene transcripts that might explain a significant GWAS finding. Towards this end, it identifies the most suitable *cis*-acting SNP for a given gene transcript. Second, the SMR approach implements a heterogeneity test (‘HEIDI' test) that allows one to distinguish linkage (where multiple causal variants underlie the association between a gene expression trait and epigenetic age acceleration) from the more interesting finding of pleiotropy (where only a single causal variant explains the association between the two traits). Third, the SMR method has been implemented in a user-friendly computer software tool that is designed specifically to deal with GWAS data and eQTL summary data.

A significant SMR test *P* value does not necessarily mean that gene expression and the trait are affected by the same underlying causal variant, as the association could possibly be due to the top associated *cis-*eQTL being in LD with two distinct causal variants. Zhu *et al*.^32^ define the scenario of several causal variants, which is of less biological interest than pleiotropy, as ‘linkage' and proposed a statistical test ‘HEIDI' for distinguishing it from pleiotropy (Supplementary Fig. 13). The null hypothesis of the HEIDI test corresponds to one of two desirable causal scenarios (causal model 1: SNP→expression→age acceleration or the pleiotropic model 2: expression← SNP→age acceleration). Thus, a nonsignificant *P* value (defined here as *P*≥0.01) of the HEIDI test is a desirable finding. Conversely, a significant HEIDI test *P* value indicates that at least two linked causal variants affect both gene expression and epigenetic age acceleration. We performed SMR in conjunction with HEIDI on the four *cis* genes in 17q11.2: *BLMH*, *CRLF3*, *EFCAB5* and *GOSR1*. As input, we used both our meta-analysis GWAS results (of epigenetic age acceleration) and *cis*-eQTL results from (1) our study, (2) GTEx and (3) BRAINEAC. The SMR analysis requires significant *cis*-eQTL relationships. For our gene expression data (1), we only analysed the subset of studies and brain regions that exhibited at least a nominally significant *cis*-eQTL (*P*<0.05) with respect to the test gene (*ECABA5* in Fig. 4 and the three remaining genes in Supplementary Fig. 10). In the SMR analysis, we used the 1000 genome individuals with European ancestry (*N*=379) as reference panel. We included the *cis-*SNPs (with MAF≥0.05) within a test gene (±1 Mb) and imposed an LD threshold of 0.9 for SNP pruning. For GTEx, we only used the *cis* genes listed in the significant eQTL (v6 version) results, stringently assessed by permutation-based thresholds at the gene level and corrected for multiple comparisons across genes and tissue types. After observing significant SMR results for *EFCAB5* in several brain regions, we conducted an *ad hoc* analysis that thresholded the FCTX *cis*-eQTL results at GTEx *P*<1.0 × 10^−3^, yielding 33 SNPs available for the SMR analysis. In (1) and (3), we set up the threshold for eQTL *P* value at 1.57 × 10^−3^ (equivalent to a chi-square value 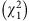 of 10) for selecting *cis*-SNPs for analysis. All SNPs selected in the SMR analysis were used in the HEIDI analysis.

### GWAS-based enrichment analysis with MAGENTA

We used the MAGENTA software^33^ to assess whether our meta-analysis GWAS results of epigenetic age acceleration are enriched with various gene sets, for example, KEGG pathways, gene ontology terms such as biological processes or molecular functions. To assign genes to SNPs, we extended gene boundaries to ±50 kb. For computational reasons, we removed categories that did not contain any genes related to age acceleration at a level of 1.0 × 10^−3^ or that contained fewer than 10 genes. The cutoffs of gene set enrichment analysis in the MAGENTA algorithm were set at 95th and 75th percentiles, which are the default parameter values for a general phenotype and for a highly polygenic trait, respectively^33^. Initially, empirical *P* values were estimated based on 10,000 permutations. For significant gene sets (empirical *P*<1.0 × 10^−4^), we estimated the final empirical *P* value using 1 million permutations. We only report gene sets whose false discovery rate (calculated by MAGENTA) was <0.25.

### GWAS-based overlap analysis of age acceleration

Our GWAS-based overlap analysis related gene sets found by our GWAS of epigenetic age acceleration with analogous gene sets found by published GWAS of various phenotypes. A description of each published GWAS study can be found in Supplementary Note 2.

The following is a brief description of the 65 published GWAS studies. Most GWAS results came from the GIANT consortium on body fat distribution^38^ such as hip and waist circumference, hip-to-waist ratio, BMI, height. Each of the 12 main GIANT GWAS results were stratified by gender (males, females and both), cross genetic ancestry (European or admixed), and adjusted for BMI. Further, we used published GWAS results from inflammatory bowel disorder^40^ and its two subtypes: Crohn's disease and ulcerative colitis, metabolic outcomes and diseases: insulin and glucose from^66^, type 2 diabetes^39^ (stage 1 and combined results), age-related macular degeneration^34^ (neovascular and geographic atrophy), Alzheimer's disease^3^ (stage 1 and combined stages 1 and 2 results), modifiers of HD motor onset^35^, Parkinson's disease^36^, attention-deficit hyperactivity disorder (ADHD), bipolar disorder, major depressive disorder, schizophrenia^37^ and longevity.

Our GWAS results of cognitive functioning traits was based on data from the Health and Retirement Study (HRS), which is a nationally representative, longitudinal study of older adults in the United States (*n*=12,452, Supplementary Table 17 (ref. 22). We either restricted the GWAS analysis to a specific ethnic group (European, African American and Amerindian ancestry) or used all individuals (denoted ‘admixed') in multivariate regression models who adjusted for principal components calculated from identity-by-state relationships (Supplementary Table 18; Supplementary Figs 20–21). We focused on two clinical traits: a longitudinal measure of age-related cognitive decline (defined in ref. 22)) and a binary variable of dementia status (defined by combining dementia assessments from the last two consecutive waves, Supplementary Information).

### MR-Egger regression

MR analyses using multiple genetic variants can be viewed as a meta-analysis of the causal estimates from each variant^41^. If the genetic variants have pleiotropic effects on the outcome, these causal estimates will be biased. MR-Egger regression offers a simple way to detect directional pleiotropy; that is, whether causal estimates from weaker variants tend to be skewed in one direction. Under a weaker set of assumptions than typically used in MR, an adaption of Egger regression (MR-Egger) can be used to detect and correct for the bias due to directional pleiotropy^41^. While the standard method of MR estimation, two-stage least squares, may be biased when pleiotropy is present, MR-Egger regression can provide a consistent estimate of the causal effect of an exposure (for example, body weight) on a trait (for example, epigenetic age acceleration). MR-Egger regression analysis requires summary-level data of SNP-exposure association and SNP-outcome association from uncorrelated SNPs. In the parlance of MR analysis, we considered two ‘exposure' variables on brain ageing: (i) hip circumference adjusted for BMI and (ii) waist circumference adjusted for BMI. While related, these two exposure variables led to two separate sets of SNPs: the first was comprised of 39 hip-associated SNPs identified from a GWAS for hip-adjusted BMI using 93,965 males of European ancestry in the GIANT consortium^38^. The second SNP set involved 29 SNPs identified from the GWAS of waist circumference using the same 93,965 males. Two meta-analysis results corresponding to the GWAS of age acceleration in PFCTX and the GWAS of neuronal proportions in PFCTX (as depicted in Fig. 1c, parts I and III) were used for SNP-outcome associations.

### URLs

1000 Genome project, http://www.1000genomes.org/

BRAINEAC, http://www.braineac.org/

DNAm age, http://labs.genetics.ucla.edu/horvath/htdocs/dnamage/

EIGENSTRAT, http://genepath.med.harvard.edu/~reich/Software.htm

GTEx, http://www.gtexportal.org/home/documentationPage#AboutGTEx

GIANT, https://www.broadinstitute.org/collaboration/giant/index.php/Main_Page

HRS, http://hrsonline.isr.umich.edu/

IMPUTE2, https://mathgen.stats.ox.ac.uk/impute/impute_v2.html

METAL, http://csg.sph.umich.edu/abecasis/Metal/

Locuszoom, http://csg.sph.umich.edu/locuszoom/

MAGENTA, https://www.broadinstitute.org/mpg/magenta/

PLINK, http://pngu.mgh.harvard.edu/~purcell/plink/

R metafor, http://cran.r-project.org/web/packages/metafor/

R WGCNA, http://labs.genetics.ucla.edu/horvath/CoexpressionNetwork/

SHAPEIT, https://mathgen.stats.ox.ac.uk/genetics_software/shapeit/shapeit.html

### Data availability

All of our data are publicly available as detailed in Table 1 and Supplementary Note 1 (dbGAP accession numbers for SNP array). DNA methylation data can be downloaded from Gene Expression Omnibus GSE59685, GSE15745, GSE36192, GSE35978, GSE38873, GSE61431, GSE36192 and GSE31694. All other data that support the findings of this study are available from the corresponding author on reasonable request.

## Additional information

**How to cite this article:** Lu, A. T. *et al*. Genetic architecture of epigenetic and neuronal ageing rates in human brain regions. *Nat. Commun.* **8**, 15353 doi: 10.1038/ncomms15353 (2017).

**Publisher's note:** Springer Nature remains neutral with regard to jurisdictional claims in published maps and institutional affiliations.

## Supplementary Material

Supplementary Information Supplementary Figures, Supplementary Tables, Supplementary Notes and Supplementary References

Supplementary Data 1 Flagged genes in functional enrichment study of SNP sets associated with epigenetic brain aging.

Supplementary Data 2 Genes that overlap between epigenetic age acceleration in ALL and at least one complex phenotype according to our GWAS overlap analysis.

Supplementary Data 3 Genes that overlap between epigenetic age acceleration in PFCTX and at least one complex phenotype according to our GWAS overlap analysis.

Supplementary Data 4 Genes that relate to the proportion of neurons in PFCTX and to at least one age-related disease according to the GWAS overlap analysis.

## Figures and Tables

**Table 1:** Overview of study data sets.

**Data**	**Age**	**Male (%)**	**Brain region**	***N***	***N***_**GWAS**_	***N***_***cis*****-eQTL**_	**Population**	**Citation**	**Availability**
Study 1	86±8.0	38	CRBLM	63	59	NA	60% ALZ	Lunnon^67^	GSE59685
	(55, 105)		PFCTX		57	NA			
Study 2	48.0±23.2	70	CRBLM	142	112	134	100% normal*	Gibbs^55^	GSE15745, GSE36192
	(16, 96)		FCTX		133	134			
			PONS		125	143			
			TCTX		125	145			
Study 3	44.3±9.6(19, 68)	63	CRBLM	147	147	130	80% PSY disorder	Zhang^68^	GSE35978, GSE38873
Study 4	64.4±17.4	61	CRBLM	37	36	NA	48% SCZ	Pidsley^69^	GSE61431
	(25, 96)		PFCTX		36	NA			
Study 5	52.3±29.8	66	CRBLM	209	201	219	100% normal*	Hernandez^70^	GSE36192, GSE31694
	(1, 102)		FCTX		201	218			
Study 6	87.9±7.3(66, 108)	32	DLPFX	302	302	294	47% ALZ†	Shulman^53^	http_ROSMAP (ref. 54)
Study 7	89.3±5.8(66, 107)	23	DLPFX	262	262	288	43% ALZ†	Shulman^53^	http_ROSMAP (ref. 54)

ALZ, Alzheimer's disease; *cis*-eQTL, *cis*-expression quantitative trait locus; CRBLM, cerebellum; DLPFX, dorsolateral prefrontal cortex; FCTX, frontal cortex; GWAS, genome-wide association study; mRNA; messenger RNA; NA, not available; PFCTX, prefrontal cortex; PONS, pons; PSY, psychiatric; SCZ, Schizophrenia, SNP, single-nucleotide polymorphism; TCTX, temporal cortex.

http_ROSMAP, https://www.synapse.org/#!Synapse:syn3168763 and https://www.synapse.org/#!Synapse:syn3388564.

The table lists seven studies that involved a total of *N*=1,796 brain tissues from 1,163 individuals who participated in our GWAS of epigenetic age acceleration in the brain. Studies 2, 3, 5, 6 and 7 are involved gene expression data including the individuals available for gene expression and SNP array data but not necessary for DNA methylation data. *N*=number of individuals passing QC (for SNP and DNA methylation array data) available for GWAS in at least one brain region; *N*_GWAS_=number of individuals passing QC available for GWAS analysis in the corresponding brain region; *N*_*cis*-eQTL_=number of individuals passing QC (for SNP and mRNA array data) available for *cis*-eQTL analysis in the corresponding brain region.

^*^Indicating neurologically normal.

^†^Including other dementia.

**Table 2:** SNPs associated with intrinsic epigenetic age acceleration of the brain.

**Meta-analysis**	**Band**	**No. of significant SNPs**	**Leading SNP**	**Nearby gene**	**Position (bp)**	**Minor/major alleles**	**MAF**	**EUR MAF**	***β*** **(s.e.)**	**Corr. (s.e.)**	**Meta** ***P*** **value**	***I***_**2**_**(%) (*****P*** **value)**
ALL	17q11.2	7	rs2054847	*SLC6A4*	28532013	A/G	0.42	0.41	−1.01 (0.20)	−0.15 (0.03)	4.5 × 10^−9^	0 (0.8)
PFCTX	1p36.12	1	rs11296960	*ECE1*	21590155	CT/C	0.47	0.47	1.07 (0.20)	0.21 (0.04)	2.2 × 10^−8^	85 (0.002)

Corr., correlation with respect to minor allele; EUR MAF, minor allele frequency calculated using 1000 genome individuals with ancestry of European (released in December 2013); MAF, mean of minor allele frequency estimates across studies weighted by study sample sizes; PFCTX, prefrontal cortex; SNP, single-nucleotide polymorphism.

Position bp based on Hg19 assembly. No. of significant SNPs=number of markers with association *P*<5.0 × 10^−8^. *β* is approximated by Corr. (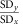), where SD_*y*_ is the pooled s.d. of age acceleration and SD_*x*_ is the s.d. of SNP covariate (coded by allele counts); SD_*y*_=4.69 for ALL and 3.60 for PFCTX. We present the loci with SNP associations at 5.0 × 10^−8^ and display the most significant SNP within each locus. Fixed-effects meta-analysis was used to estimate the correlation coefficient and s.e. (‘Corr. (s.e.)') between the minor allele and epigenetic age acceleration across studies. The corresponding meta-analysis *P* values can be found in the column ‘Meta *P*'. The prefrontal cortex (PFCTX) includes the dorsolateral prefrontal cortex.

**Table 3:** Summary data-based Mendelian randomization analysis of *EFCAB5* expression versus age acceleration.

**Region**	**Combined studies**	**Our study**			**GTEx**			**UK**		
	***P***_**SMR**_	***β***	***P***_**SMR**_	***P***_**HEIDI**_	***β***	***P***_**SMR**_	***P***_**HEIDI**_	***β***	***P***_**SMR**_	***P***_**HEIDI**_
CRBLM	1.7 × 10^−10^	−6.20*	3.4 × 10^−4^	0.9	−1.73	7.1 × 10^−5^	0.85	−5.71	1.2 × 10^−4^	0.15
FCTX	7.8 × 10^−6^	—	—	—	−1.75	3.0 × 10^−3^	0.09	−7.88	3.9 × 10^−4^	0.03
PFCTX	9.2 × 10^−3^	−14.0†	9.2 × 10^−3^	0.8	—	—	—	—	—	—
TCTX	2.9 × 10^−4^	—	—	—	—	—	—	−6.93	2.9 × 10^−4^	0.15
ALL‡	1.8 × 10^−5^	—	—	—	—	—	—	−9.57	1.8 × 10^−5^	0.54
Combined regions	1.2 × 10^−16^									

*cis*-eQTL, *cis*-expression quantitative trait locus; CRBLM, cerebellum; FCTX, frontal cortex; PFCTX, prefrontal cortex; TCTX, temporal cortex.

— denotes ‘not available' or ‘not tested'.

Results from summary data-based Mendelian randomization analysis (SMR) in conjunction with heterogeneity in dependent instruments (HEIDI) analysis. SMR and HEIDI were performed using our GWAS results surrounding the gene *EFCAB5* and the *cis*-eQTL results from (1) our individual-level study, (2) GTEx and (3) BRAINEAC (denoted by UK), respectively. The SMR test yields a slope estimate *β* for the change in epigenetic age acceleration per unit *EFCAB5* expression and the associated *P* value (*P*_SMR_). The HEIDI test yields a *P* value (*P*_HEIDI_) for interpreting the association between *EFCAB5* and age acceleration, where nonsignificant *P*_HEIDI_ (≥0.01) suggests *EFCAB5* expression and age acceleration are affected by the same causal variants. The column ‘Combined studies' presents the SMR *P* value *P*_SMR_ of each test region, combined across the three study sets using Stouffer's *Z* score approach. The ‘Combined studies' *P*_SMR_ values of CRBLM, FCTX, PFCTX and TCTX were combined across regions by Stouffer's approach as well, yielding an overall assessment for the association between *EFCAB5* and epigenetic age acceleration in brain with. *P*_SMR_=1.2 × 10^−16^ (see row ‘Combined regions').

^*^The analysis result from our study was performed on study 3.

^†^The analysis result from our study was performed on a combined sample of studies 6 and 7.

^‡^The expression averaged across the ten brain regions from the UK database.

**Table 4:** GWAS-based overlap analysis between traits.

**GWAS**	**POP**	**Sex**	**Hypergeometric** ***P***		
			**AgeAccel in ALL**	**AgeAccel in PFCTX**	**propN in PFCTX**
*Part I: age-related neurogenetic traits*					
Neurodegenerative and neuropsychiatric disorders					
AMD	EUR+ASN	M, F	0.9	>0.9	**3.8 × 10**^−**6**^
AMD geographic atrophy	EUR+ASN	M, F	0.7	0.9	**1.5 × 10**^−**7**^
AMD neovascular	EUR+ASN	M, F	>0.9	>0.9	**1.4 × 10**^−**12**^
ALZ*	EUR	M, F	0.8	**4.9 × 10**^−**3**^	>0.9
Parkinson's disease	EUR	M, F	0.8	>0.9	**8.6 × 10**^−**3**^
Schizophrenia	EUR+ASN	M, F	>0.9	>0.9	**1.6 × 10**^−**9**^
Cognitive functioning from HRS					
Cognitive decline (slope)	Admixed	M, F	0.2	**1.2 × 10**^−**3**^	0.2
Dementia	Admixed	M, F	0.6	>0.9	**5.3 × 10**^−**4**^
	EUR	M, F	2.0 × 10^−2^	**1.2 × 10**^−**3**^	3.6 × 10^−2^
	AFR	M, F	**5.4 × 10**^−**4**^	**2.6 × 10**^−**3**^	0.2
					
*Part II Body fat distribution, inflammatory outcomes and other age-related traits*					
GIANT body fat distribution^†^					
Hip	Admixed	M	0.8	0.3	**5.2 × 10**^−**7**^
	EUR	M	>0.9	0.1	**1.6 × 10**^−**7**^
Hip adj. BMI	Admixed	M, F	0.8	**3.3 × 10**^−**3**^	**1.1 × 10**^−**4**^
	EUR	M, F	>0.9	2.6 × 10^−2^	**1.5 × 10**^−**3**^
	Admixed	M	>0.9	**1.4 × 10**^−**3**^	**4.1 × 10**^−**9**^
	EUR	M	>0.9	**6.4 × 10**^−**3**^	**1.1 × 10**^−**9**^
WC adj. BMI	EUR	M, F	>0.9	8.1 × 10^−2^	**3.3 × 10**^−**3**^
	Admixed	M	0.8	**6.0 × 10**^−**4**^	**6.0 × 10**^−**4**^
	EUR	M	0.9	**2.5 × 10**^−**4**^	**9.9 × 10**^−**5**^
WHR	Admixed	M	0.1	>0.9	**1.4 × 10**^−**3**^
	EUR	M	0.2	>0.9	**6.5 × 10**^−**3**^
WHR adj. BMI	Admixed	M, F	>0.9	>0.9	**1.1 × 10**^−**4**^
	EUR	M, F	>0.9	>0.9	**4.0 × 10**^−**5**^
	Admixed	M	>0.9	0.9	**4.8 × 10**^−**6**^
	EUR	M	>0.9	>0.9	**4.6 × 10**^−**6**^
Inflammatory bowel disorder					
IBD	EUR	M, F	0.8	>0.9	**9.2 × 10**^−**12**^
IBD Crohn's disease	EUR	M, F	0.6	>0.9	**6.0 × 10**^−**9**^
IBD ulcerative colitis	EUR	M, F	>0.9	>0.9	**<1.0 × 10**^−**20**^
Metabolic outcomes and diseases^†^					
T2D stage 1	EUR	M, F	0.2	0.9	**2.8 × 10**^−**13**^
T2D combined	EUR+SAS	M, F	0.7	0.4	**1.4 × 10**^−**4**^

Adj., adjusted; AFR, Africans; ALZ, Alzheimer's disease; AMD, age-related macular degeneration; AMR, Americas; ASN, Asians; EUR, Europeans; F, females; FDR, false discovery rate; GIANT, genetic investigation of anthropometric traits (see URL); Hip adj. BMI, hip-adjusted body mass index; HRS, Health Retirement Study (see URL); IBD, inflammatory bowel disorder; M, males; SAS, southern Asians; T2D, type 2 diabetes; WC, waist circumference; WHR, waist-to-hip ratio.

The table presents a total of 30 overlap results with hypergeometric *P*<0.01 using the genes related to epigenetic age acceleration (AgeAccel) in (1) all brain regions (ALL) or (2) in the prefrontal cortex (PFCTX), or (3) the genes related to the proportion of neurons (propN) in the prefrontal cortex. The gene sets were thresholded at the top 2.5%. There are 506 (455) genes listed in the top 2.5% across *n*=20,273 (18,218) autosomal genes that have a suggestive relationship with brain age acceleration of all brain regions (ALL), age acceleration of the PFCTX or the proportion of neurons in the prefrontal cortex, based on hg19 (hg18) assembly. FDR *q*≤0.05 marked in bold, evaluated based on Benjamin Hochberg method, as listed in Supplementary Table 11 for numerical results in ALL and PFCTX, and in Supplementary Table 13 for numerical results in NP.

^*^The GWAS results from stage 1 analysis.

Calculations based on Hg19 assembly, unless marked in † otherwise.
