# Supplementary material for: Genetic architecture of epigenetic and neuronal ageing rates in human brain regions
Source: Nat Commun. 2017 May 18;8:15353. doi: 10.1038/ncomms15353 (PMC5454371; doi:10.1038/ncomms15353)
Supplement: Supplementary Information — Supplementary Figures, Supplementary Tables, Supplementary Notes and Supplementary References [file ncomms15353-s1.pdf]

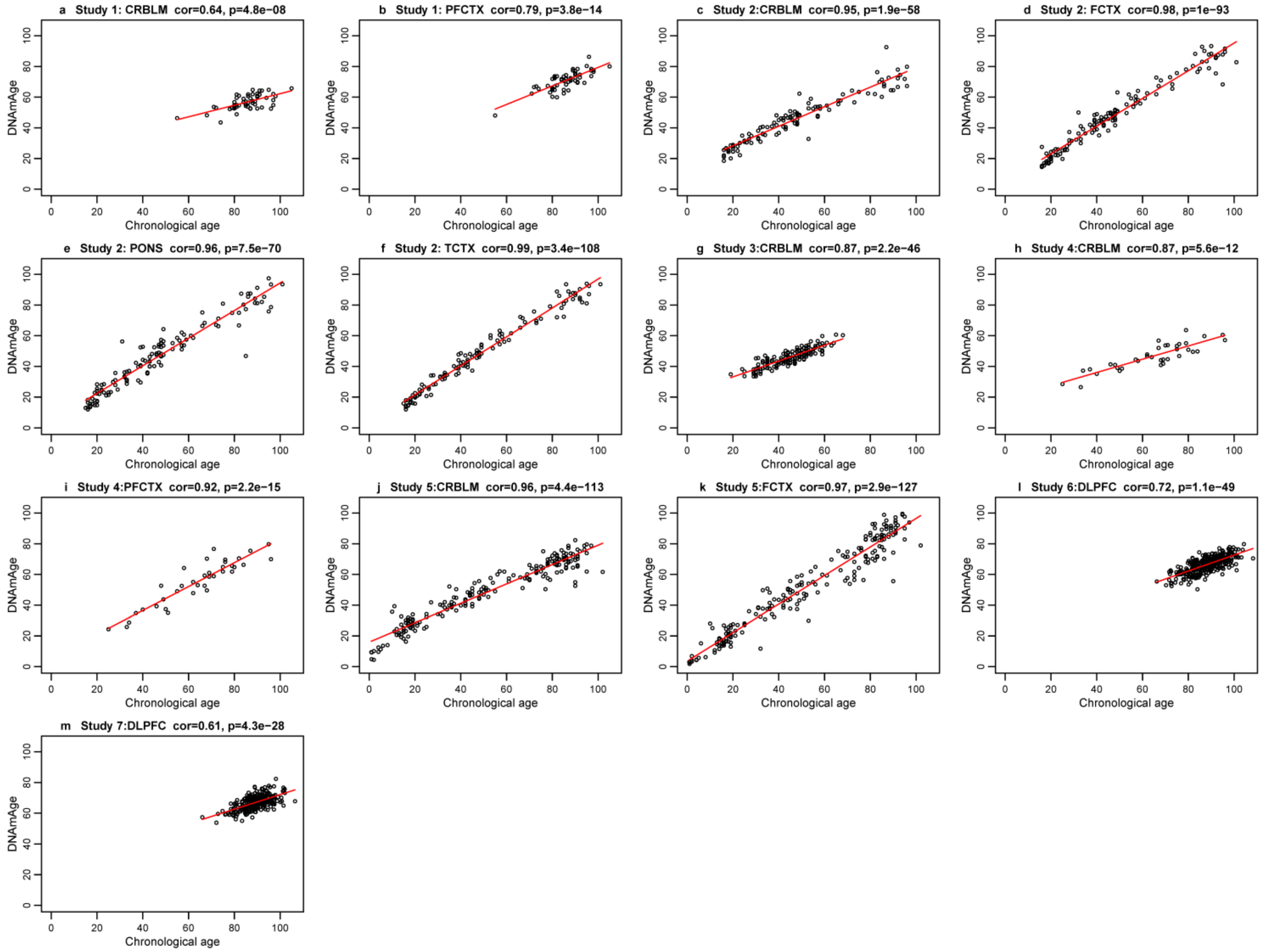

Supplementary Figure 1: DNAm age versus chronological age. (a-m) scatter plots of DNA methylation age versus chronological age (x-axis) corresponding to the different studies. Each panel reports the Pearson correlation coefficients and a corresponding p-value.

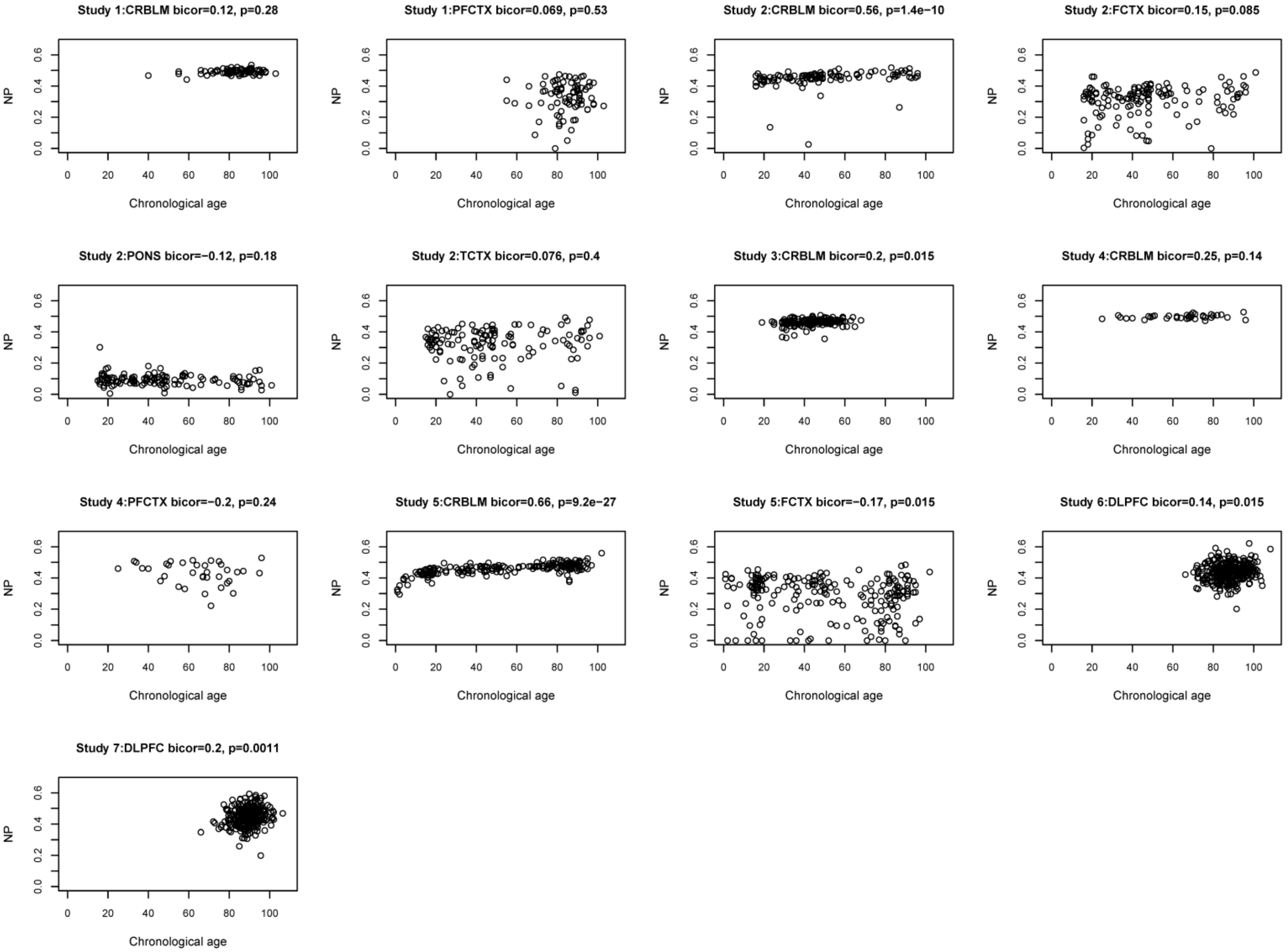

Supplementary Figure 2: Correlation of neuronal proportion versus chronological age. Scatter plots between neuronal proportion (NP) and chronological age (x-axis). The captions report the biweight midcorrelation (bicor) which is robust correlation coefficient [1]. The panels correspond to different studies and brain regions.

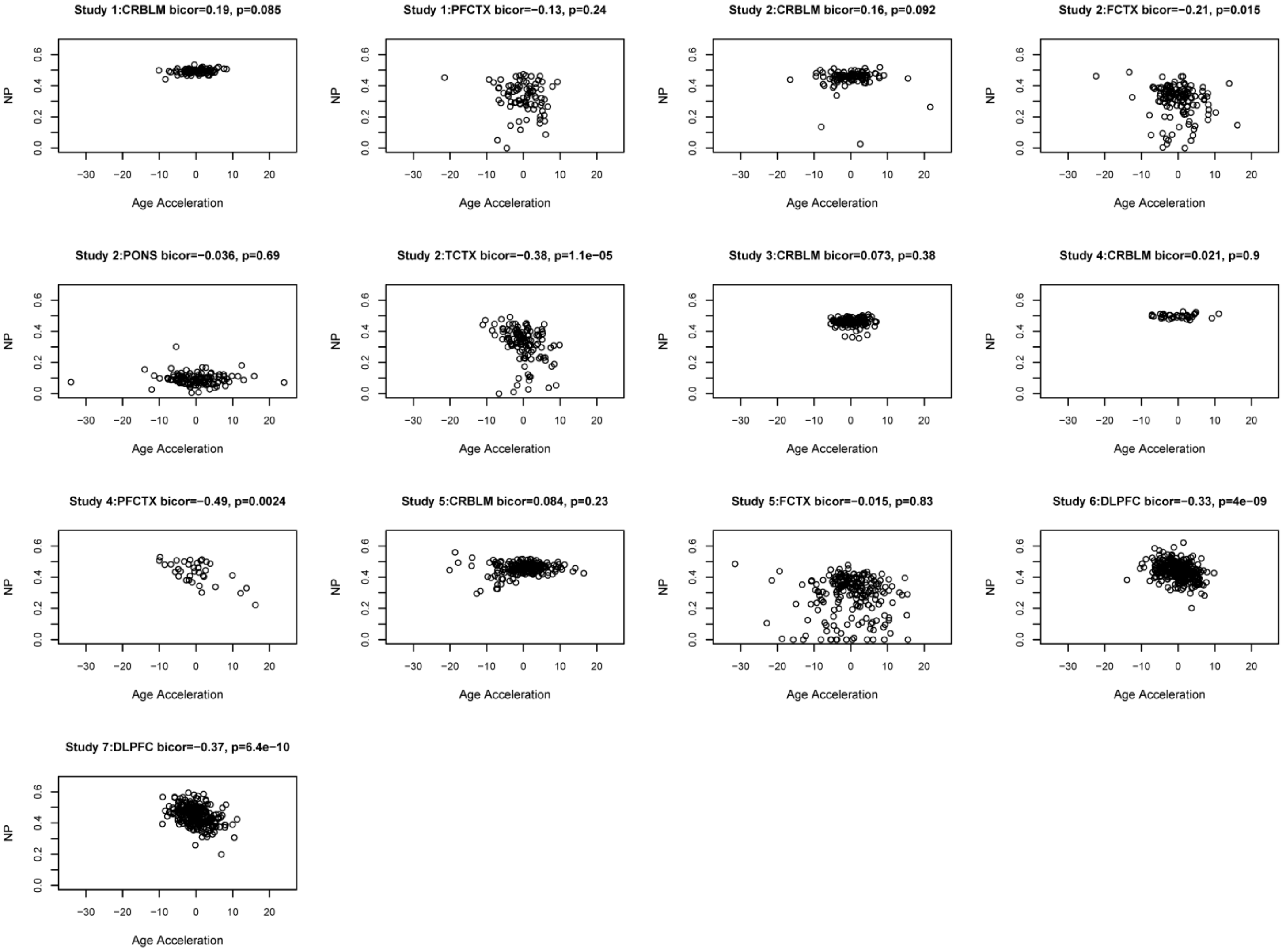

Supplementary Figure 3: Proportion of neurons versus epigenetic age acceleration stratified by study and brain region. Age acceleration was defined as raw residual resulting from regressing DNAm age on chronological age.

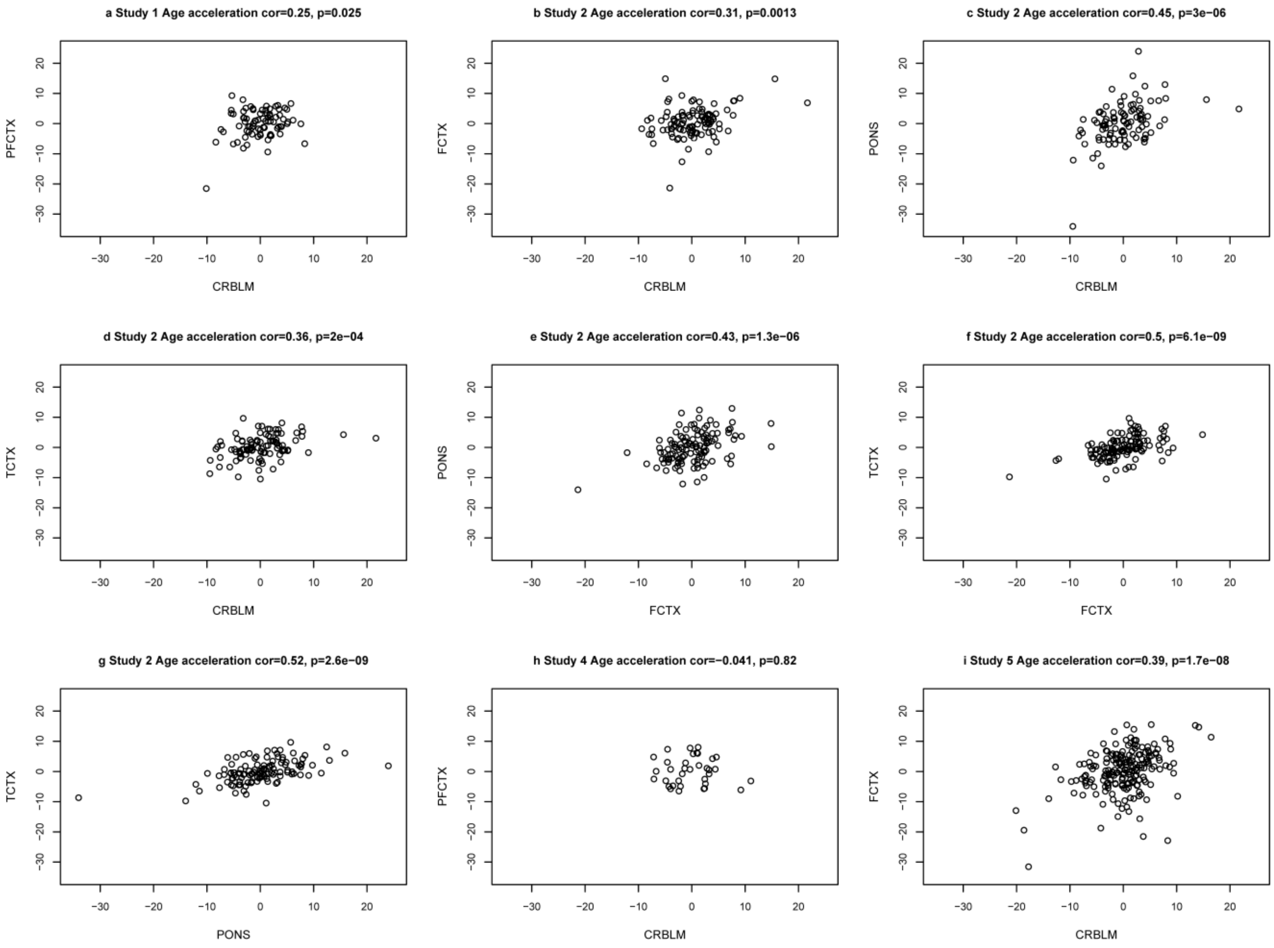

Supplementary Figure 4: Preservation of epigenetic age acceleration across brain regions. The figure depicts the scatter plots of age acceleration traits across different pairings of brain regions from studies 1,2 ,4 and 5. The measure of age acceleration was adjusted for the proportion of neurons, gender and disease status (described in Supplementary Table 4). Each panel reports the Pearson correlation coefficients and a corresponding p-value.

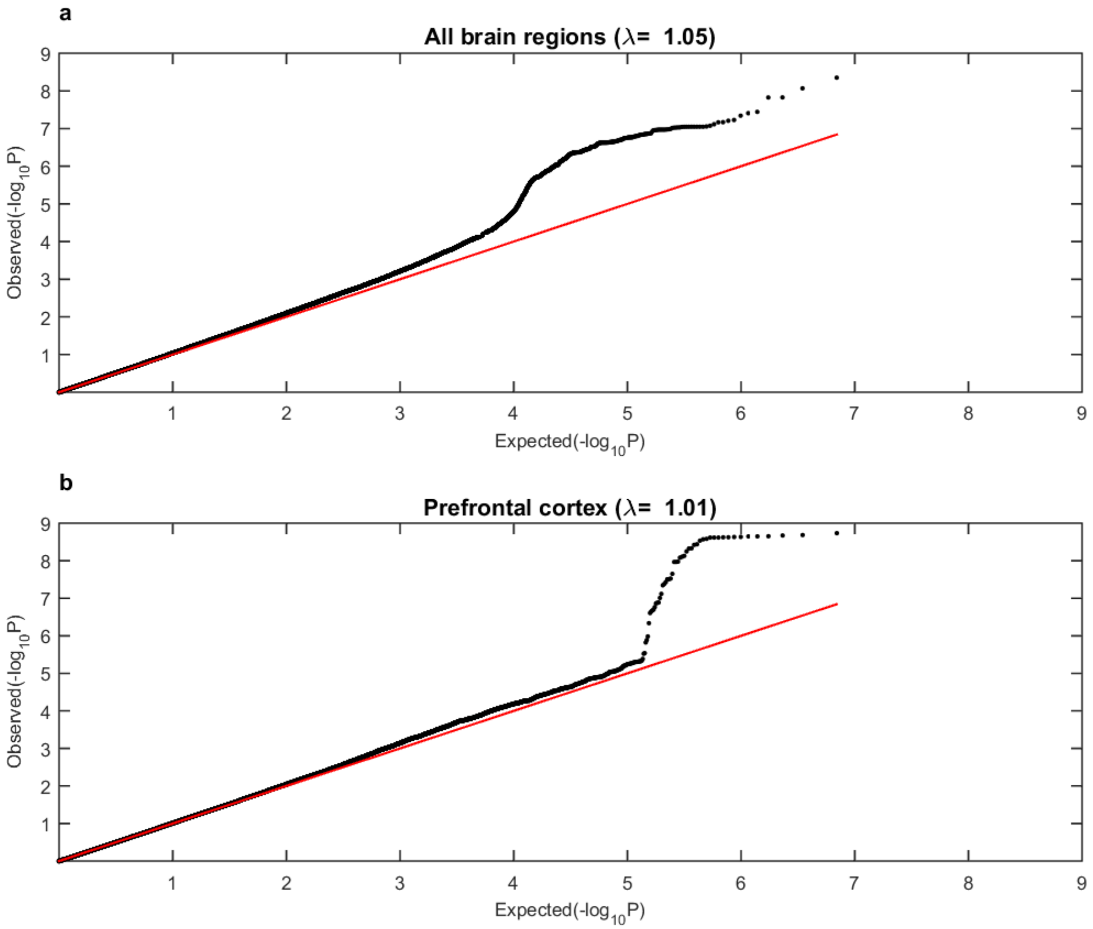

Supplementary Figure 5: QQ plots for evaluating genomic inflation in the meta-analysis. The figure presents the QQ plots based on the GWAS results using (a) all brain regions tissues, and (b) the tissues in prefrontal cortex (PFCTX), respectively. The black and red lines represent the observed and expected meta P values, respectively.

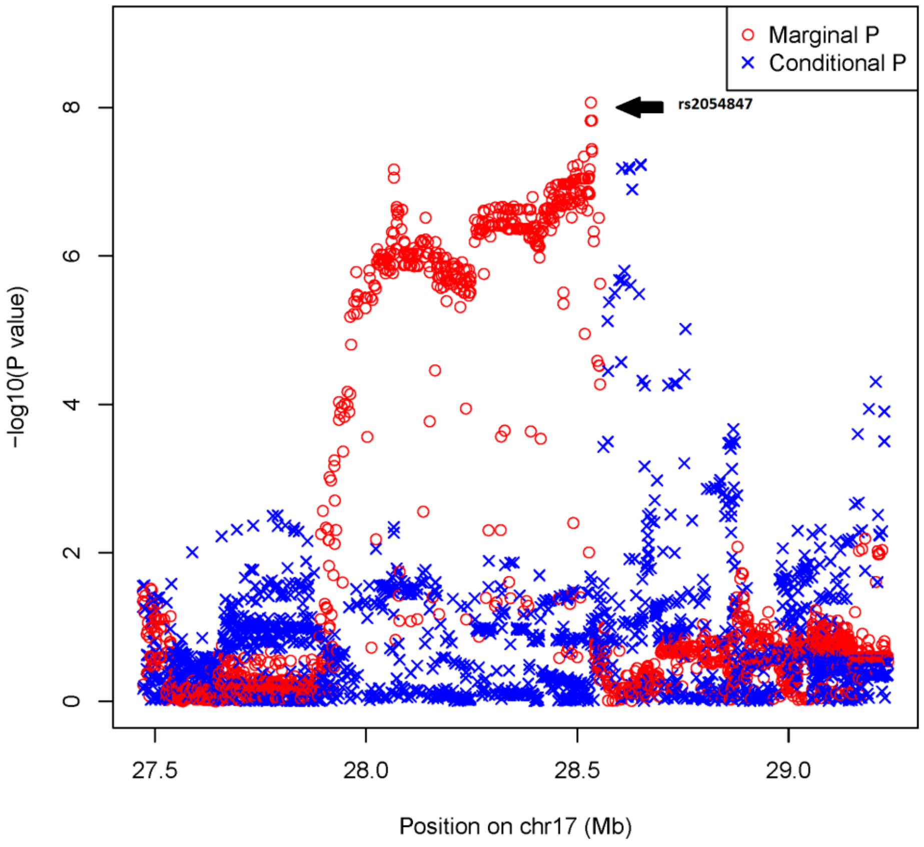

Supplementary Figure 6: Regional association analysis within 17q11.2 conditional on the leading SNP rs2054847. The plot displays the association results of meta-analysis surrounding the leading SNP rs2054847 in the GWAS locus 17q11.2. Two types of P values are presented: (1) the marginal P values (colored in red), and (2) the GCTA-based conditional P values on rs2054847 (colored in blue). The association signals for those SNPs with marginal $P < 5\text{x}10^{-7}$ significantly dropped in the conditional analysis, indicating that their signals were led by the leading SNP rs2054847.

a) rs1128156 in chromosome 17 near SLCA64

<u>127 cell/tissue types</u>

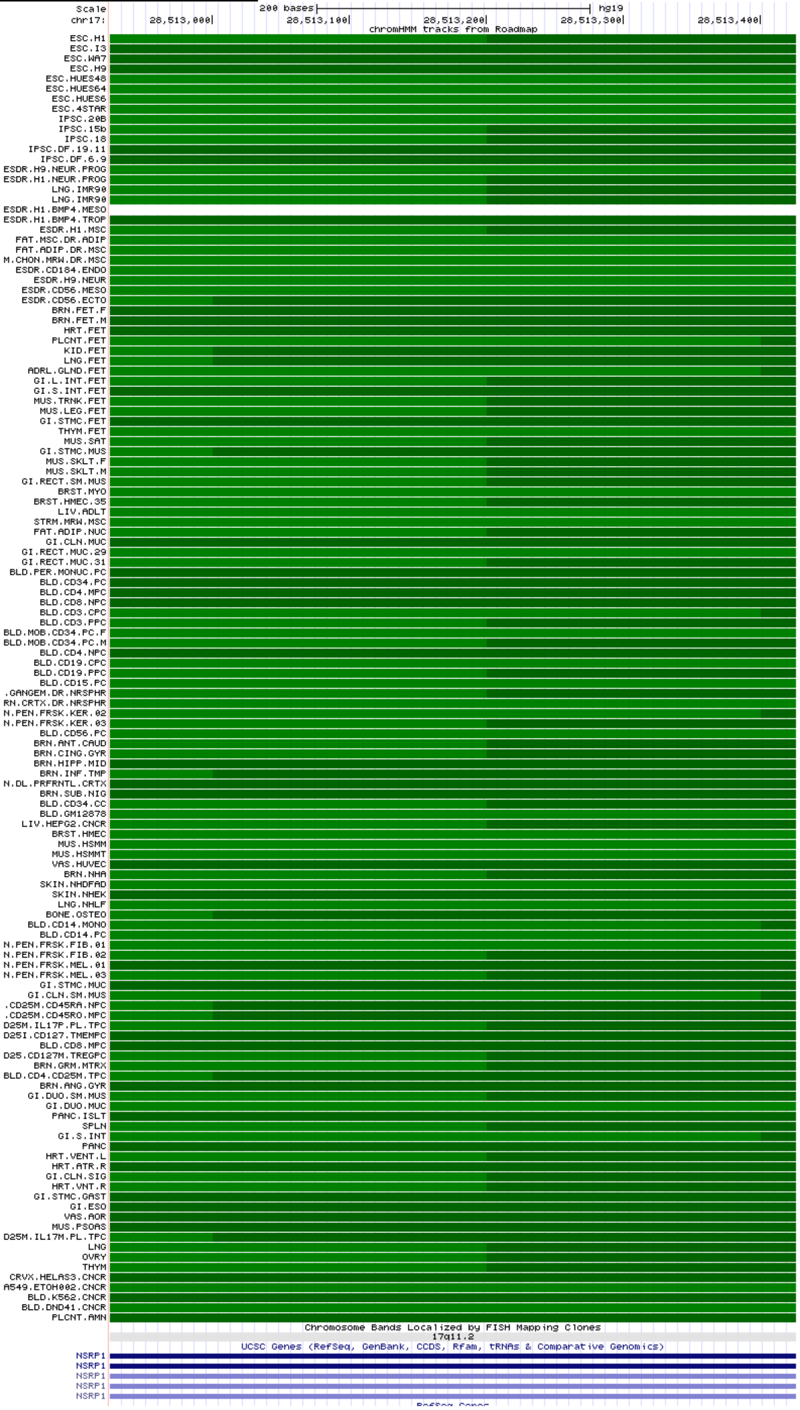

b) rs2054847 in chromosome 17 SLC6A4

<u>127 cell/tissue types</u>

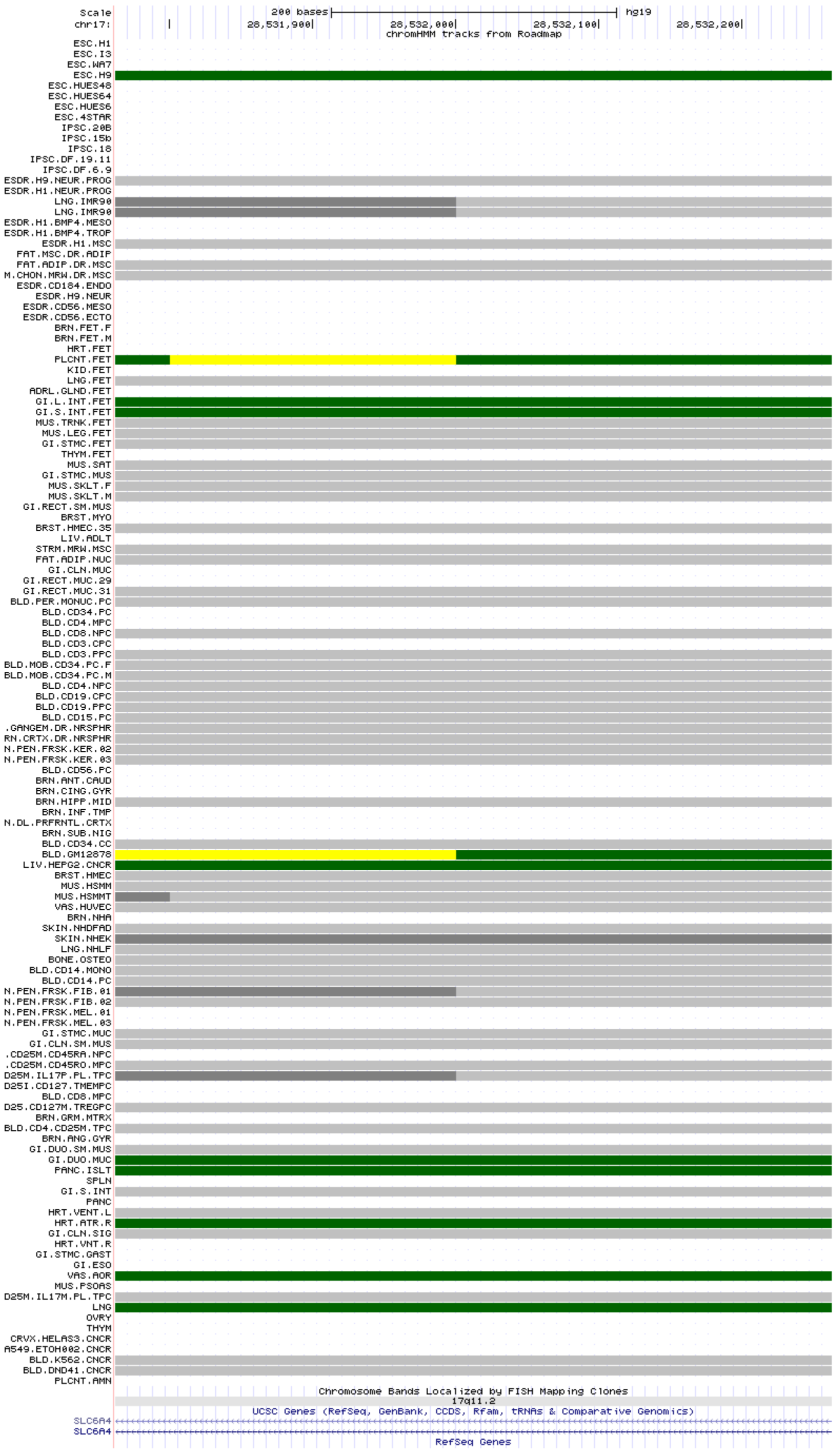

c) rs11296960 in chromosome 1 within ECE1

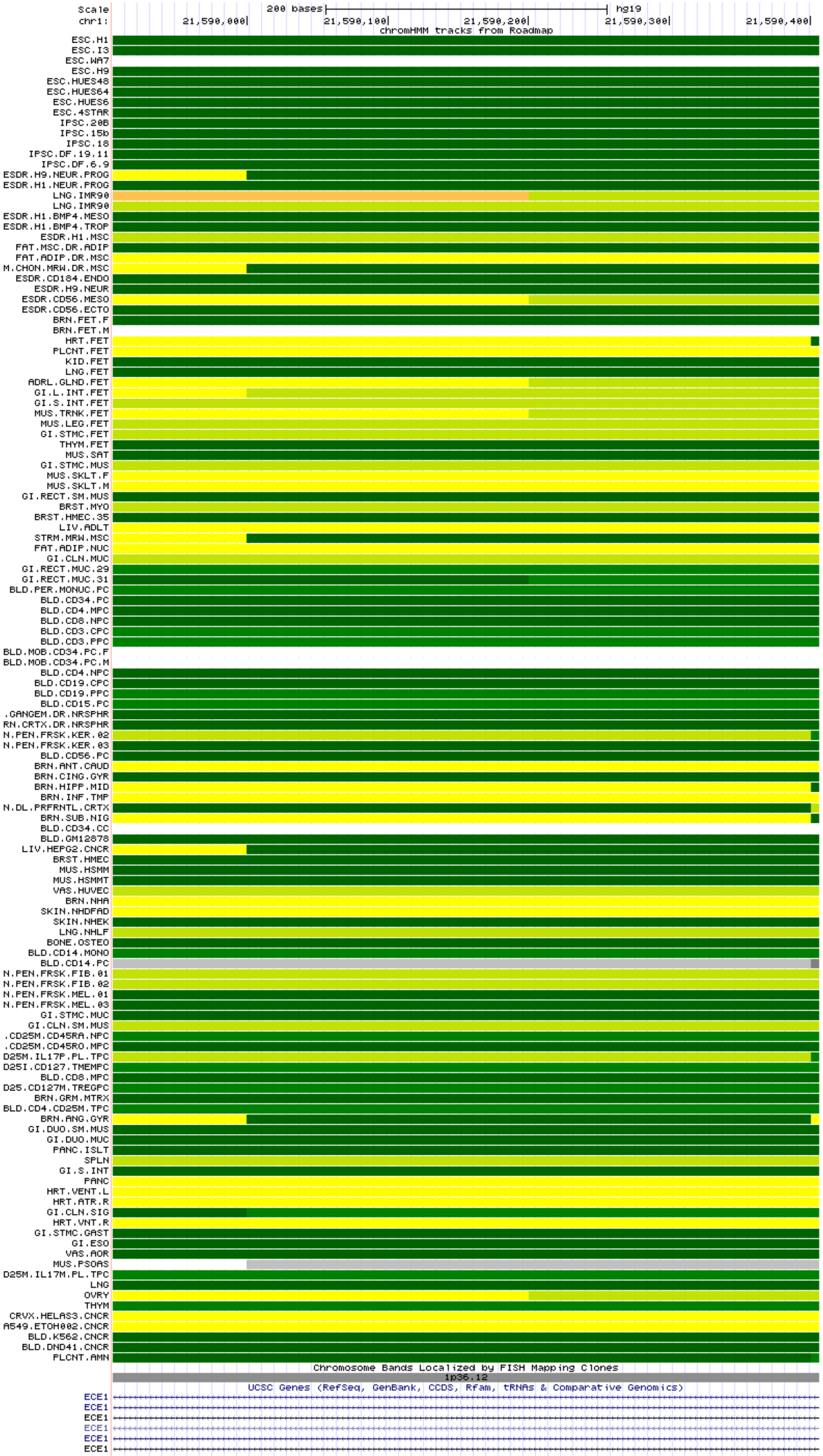

Supplementary Figure 7: Chromatin state analysis based on Roadmap Epigenomics and ENCODE. For our genome-wide significant SNP, we used results from a chromatin state analysis (15 primary states in 127 diverse human cell/tissue types) based on the hidden Markov model (HMM) [2]. The HMM classifies DNA regions dynamically into one of the 15 primary states as described below.

- State 1 - Red - TssA (Active_TSS)
- State 2 - OrangeRed - TssAFlnk (Flanking_Active_TSS)
- State 3 - LimeGreen - TxFlnk (Transcr_at_gene_5_and_3primer)
- State 4 - Green - Tx (Strong_transcription)
- State 5 - DarkGreen - TxWk (Weak_transcription)
- State 6 - GreenYellow - EnhG (Genic_enhancers)
- State 7 - Yellow - Enh (Enhancers)
- State 8 - MediumAquamarine - ZNF/Rpts (ZNF_genes&repeats)
- State 9 - PaleTurquoise - Het (Heterochromatin)
- State 10 - IndianRed - TssBiv (Bivalent/Poised_TSS)
- State 11 - DarkSalmon - BivFlnk (Flanking_Bivalent_TSS/Enh)
- State 12 - DarkKhaki - EnhBiv (Bivalent_Enhancer)
- State 13 - Silver - ReprPC (Repressed_PolyComb)
- State 14 - Gainsboro - ReprPCWk (Weak_Repressed_PolyComb)
- State 15 - White - Quies (Quiescent/Low)

More details can be found in

https://genome.ucsc.edu/cgi-bin/hgTrackUi?hgsid=433891379_Ri32gCKnmDLSQiE1ZgjJAgfHAo6H&c=chr16&g=hub_24125_RoadmapConsolidatedAssaya27004

a)

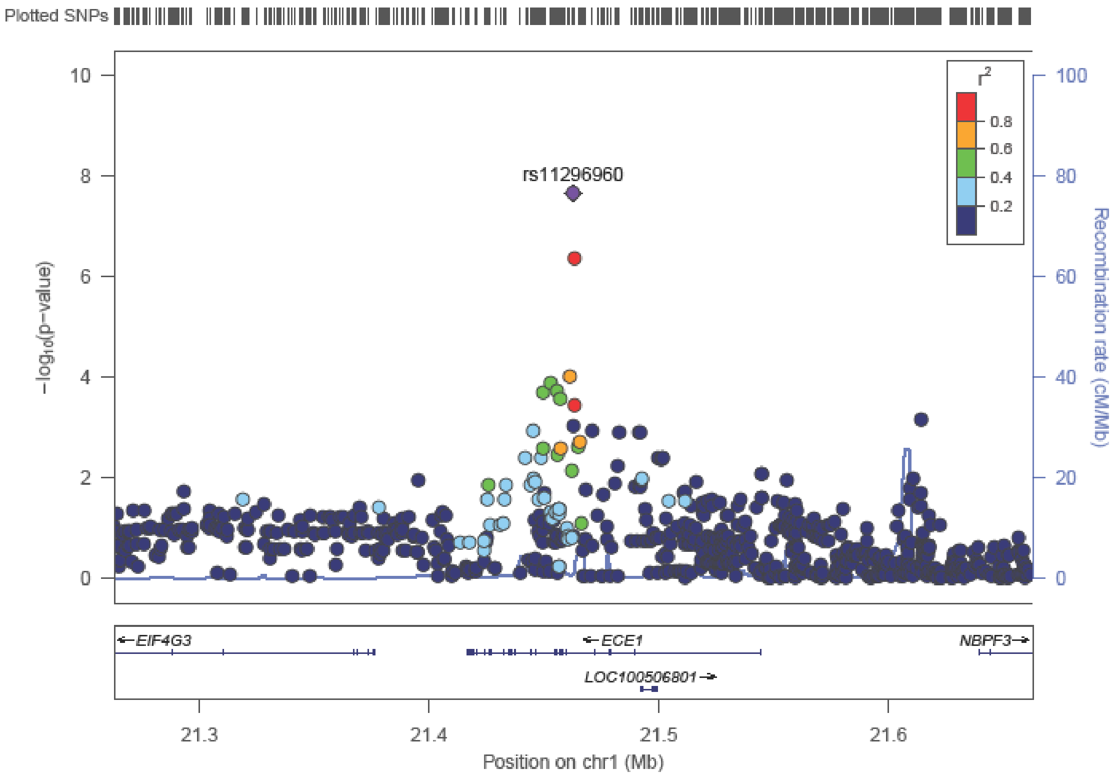
Plotted SNPs
rs11296960
−log10(p−value)
Recombination rate (cM/Mb)
r2
0.8
0.6
0.4
0.2
10
8
6
4
2
0
100
80
60
40
20
0
EIF4G3
ECE1
NBPF3
LOC100506801
21.3
21.4
21.5
21.6
Position on chr1 (Mb)

b)

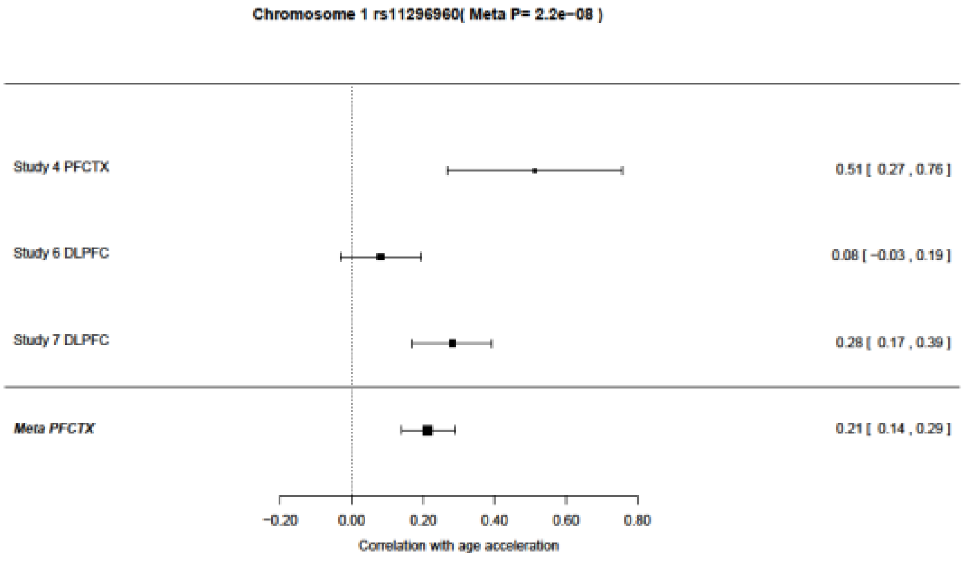
Chromosome 1 rs11296960(Meta P= 2.2e−08)
Study 4 PFCTX
0.51 [0.27 , 0.76]
Study 6 DLPFC
0.08 [−0.03 , 0.19]
Study 7 DLPFC
0.28 [0.17 , 0.39]
Meta PFCTX
0.21 [0.14 , 0.29]
−0.20
0.00
0.20
0.40
0.60
0.80
Correlation with age acceleration

Supplementary Figure 8: Detailed analysis of locus 1p36.12 associated with intrinsic epigenetic age acceleration in PFCTX. (a) The association *P* values resulted from the meta-analysis that combined GWAS of age acceleration in PFCTX from studies 1, 4, 6 and 7. The colors visualize linkage disequilibrium (LD) r^2 between rs11296960 (colored in purple) and neighboring SNPs in 1p36.12. (b) The forest plot displays the individual association results of the GWAS SNP rs11296960 with age acceleration in studies 4, 6 and 7. The genotypes of rs11296960 were missing in study 1. The estimate *Meta PFCTX P*=$2.2\text{x}10^{-8}$ reports the meta-analysis results across the 3 GWAS studies.

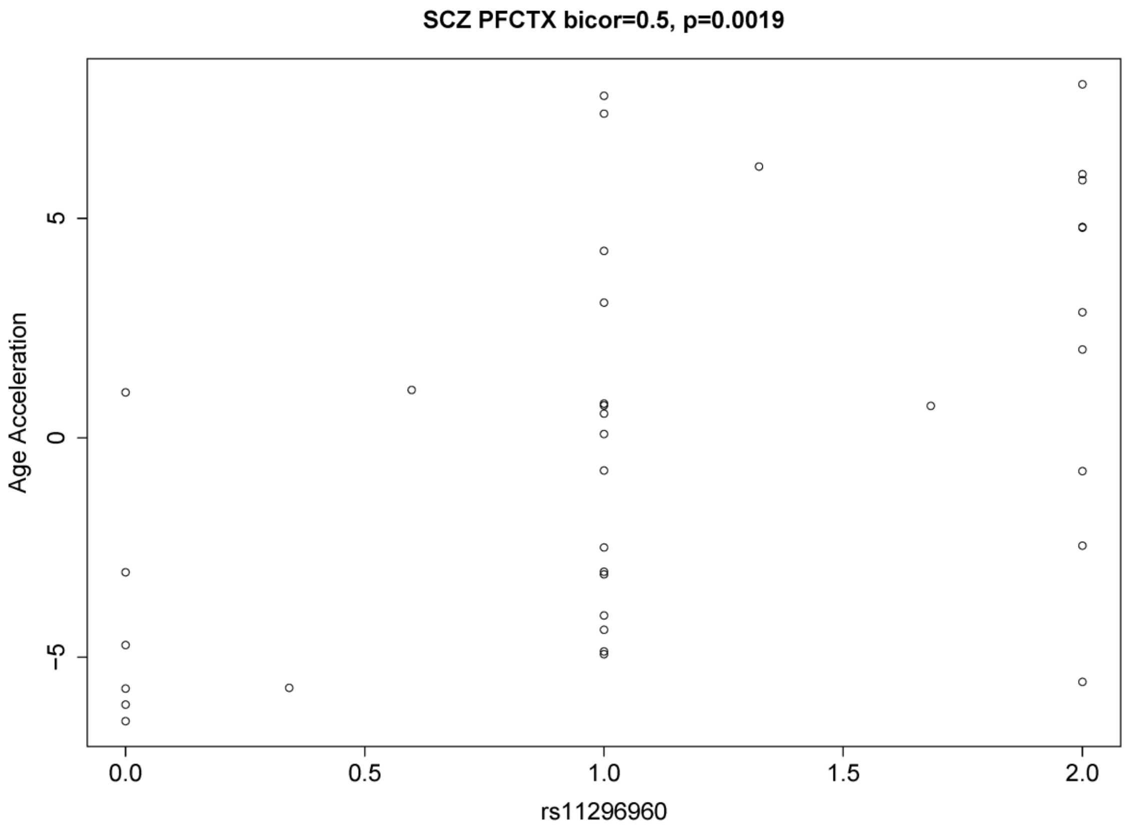

Supplementary Figure 9: Sensitivity analysis for chromosme 1 rs11296960 in study 4. The figure displays the scatter plot of age acceleration in prefrontal cortex (y-axis) versus the dosage of rs11296960 with respect to the minor allele (INDEL variant CT) in study 4 (*N*=36). The INDEL marker was significantly correlated with age acceleration (Pearson correlation r=0.50, $P = 2.3 \times 10^{-4}$). Here we used a robust correlation , bicor, which is robust to outliers[1]. Note that the INDEL shows the same magnitude for the robust correlation with age acceleration (bicor=0.50, $P = 1.9 \times 10^{-3}$), which shows that the correlation is not affected by outliers.

a)

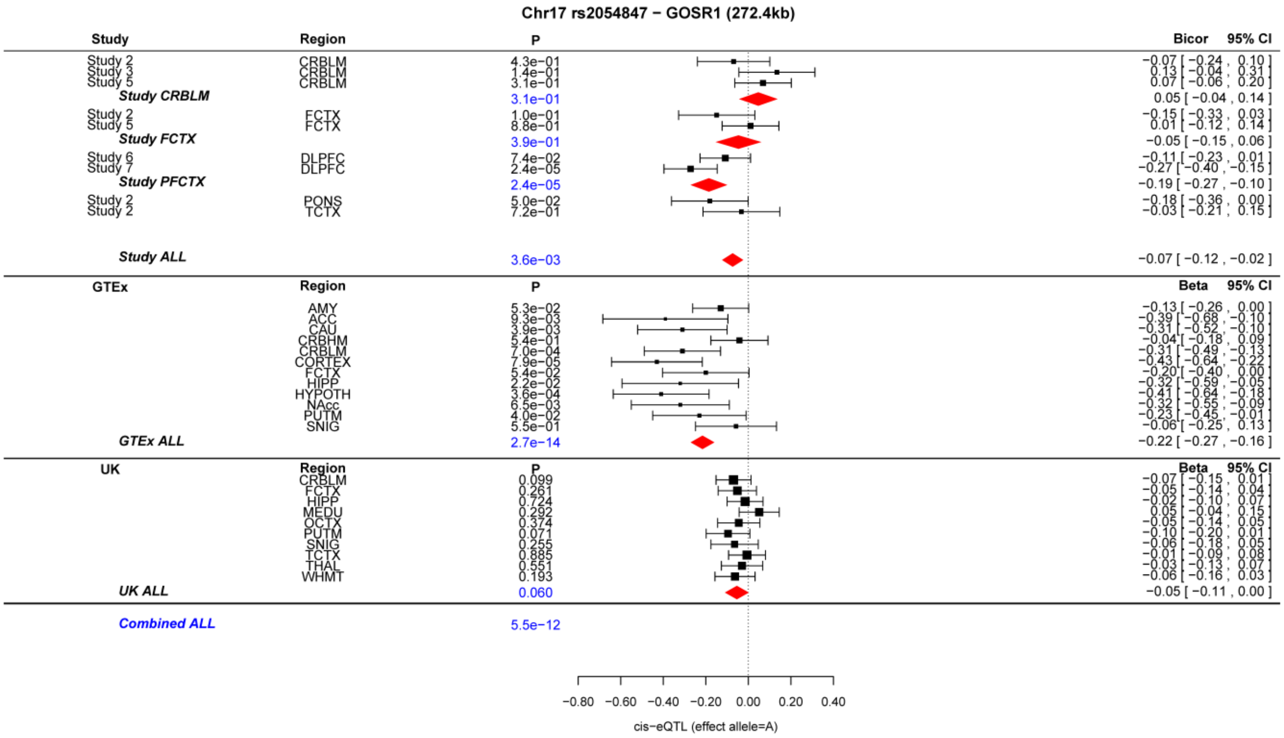

Chr17 rs2054847 − GOSR1 (272.4kb)
Study
Region
P
Bicor 95% CI
Study CRBLM
Study FCTX
Study PFCTX
Study ALL
GTEx
Beta 95% CI
GTEx ALL
UK
UK ALL
Combined ALL
5.5e−12
cis−eQTL (effect allele=A)

b)

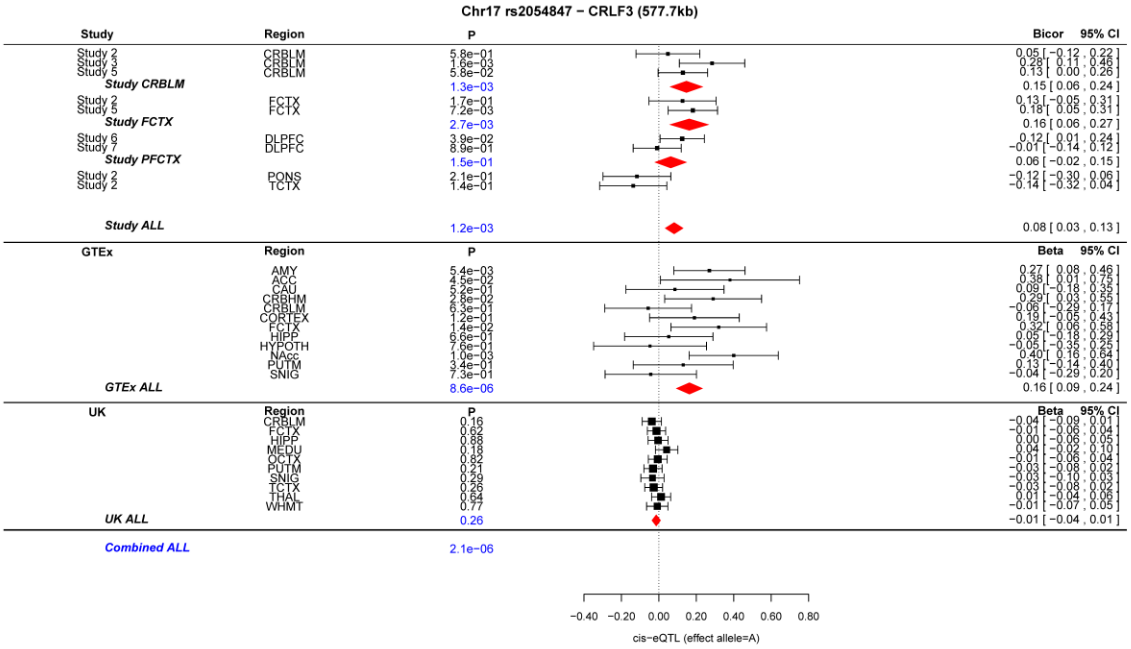

Chr17 rs2054847 − CRLF3 (577.7kb)
Study
Region
P
Bicor 95% CI
Study CRBLM
Study FCTX
Study PFCTX
Study ALL
GTEx
Beta 95% CI
GTEx ALL
UK
UK ALL
Combined ALL
2.1e−06
cis−eQTL (effect allele=A)

c)

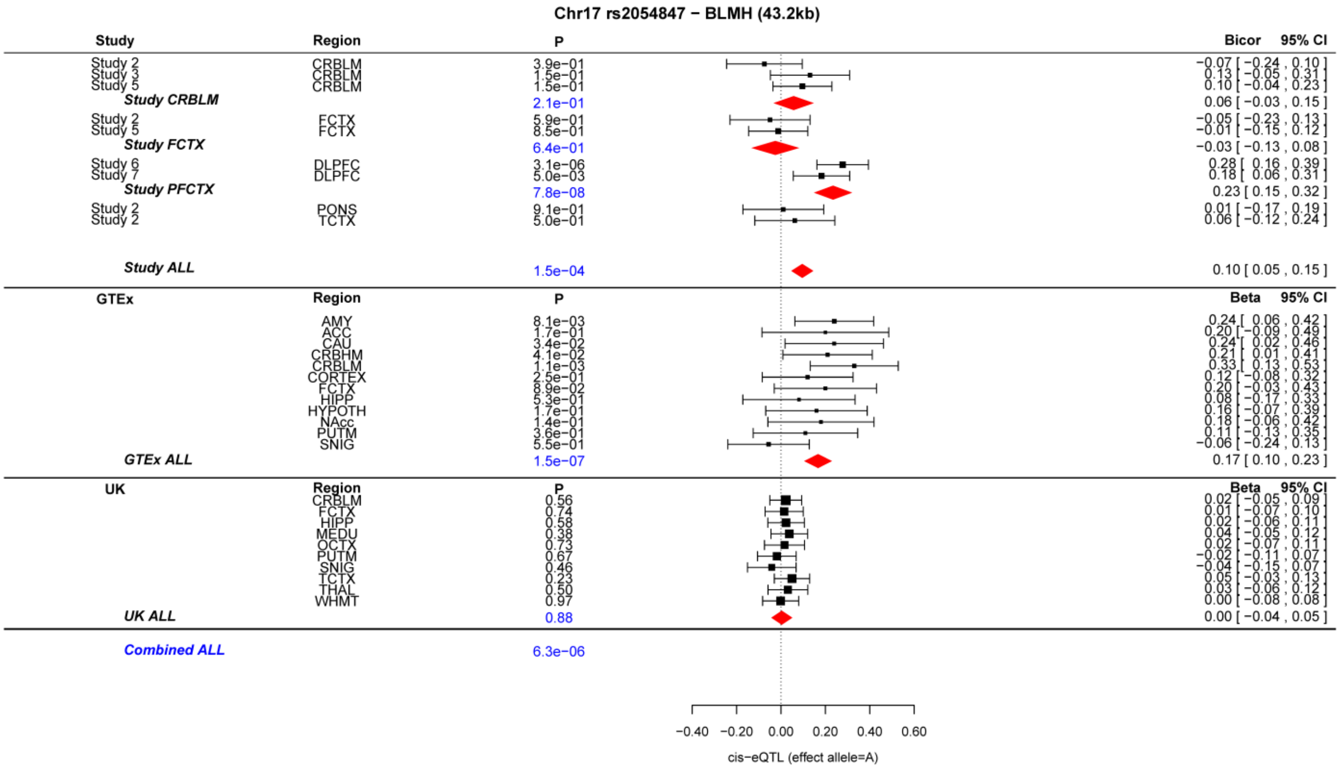

Supplementary Figure 10: Meta analysis forest plots of chromosome 17 rs2054847 evaluating *cis*-effects of proxmimal genes. The forest plots display the significant *cis*-eQTL results for pairs of (a) rs2054847 – *GOSR1* and (b) rs2054847 – *BLMH*, and (c) rs2054847 –*CRLF3* respectively. The descriptions are same as in Figure 4 of the main text.

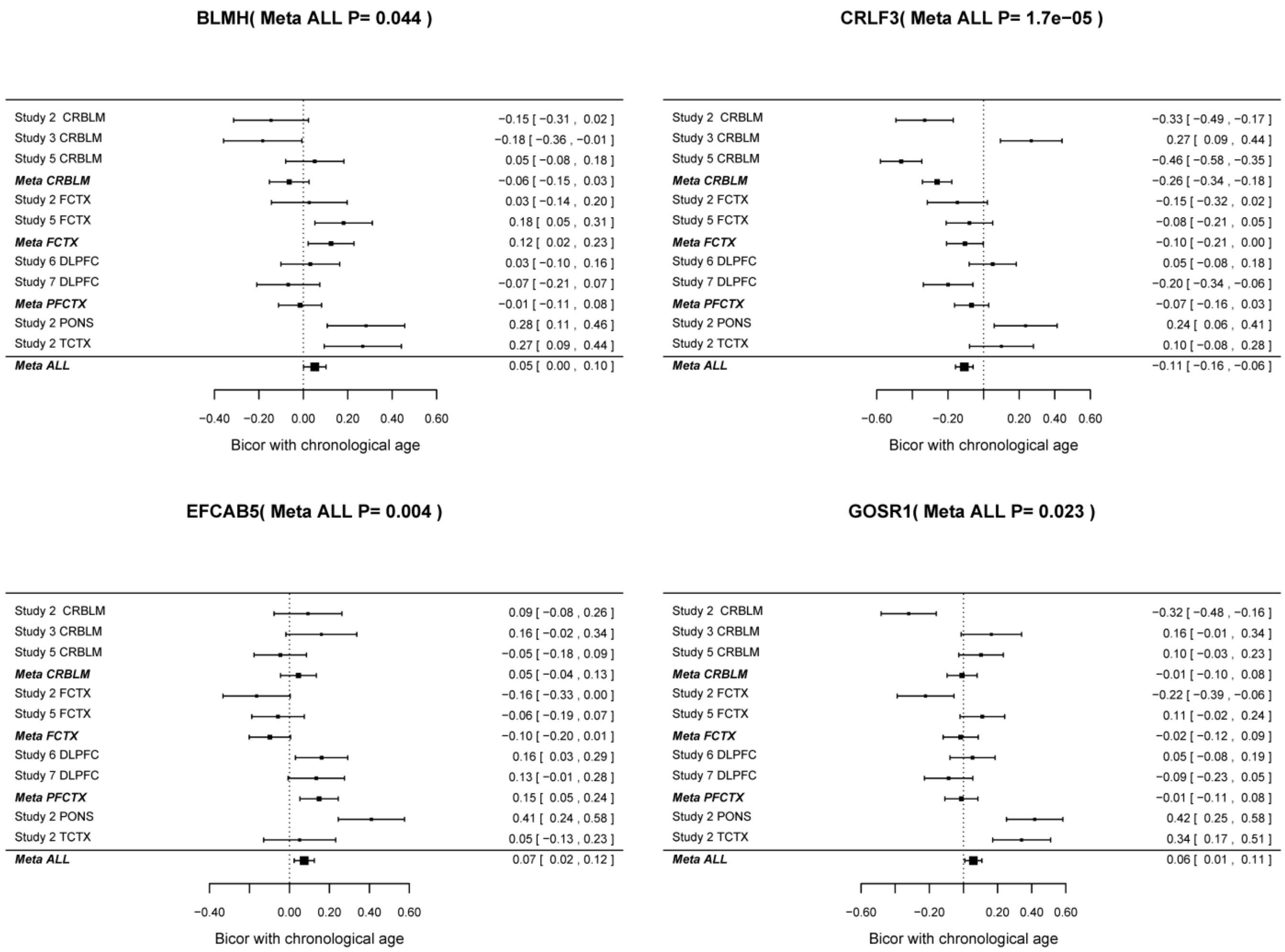

Supplementary Figure 11: Chronological age correlated with expression levels of genes in 17q11.2. The meta-analysis forest plots summarize the correlation between chronological age and the expression levels of (a) *BLMH*, (b) *CRLF3*, (c) *EFCAB5*, and (d) *GOSR1*, respectively. Each panel reports robust correlation coefficients based on our samples (up to 1705 brain tissues). We used the following abbreviations: cerebellum (CRBLM), dorsolateral prefrontal cortex (DLPFC), frontal cortex (FCTX), pons (PONS), and temporal cortex (TCTX). Meta-analysis was used to combine individual results from CRBLM into a single estimate, *Meta CRBLM*, in an analogous for *Meta FCTX* and *Meta PFCTX* (results from DLPFC). We combined all our study results into a single estimator, *Meta ALL*.

a) BLMH

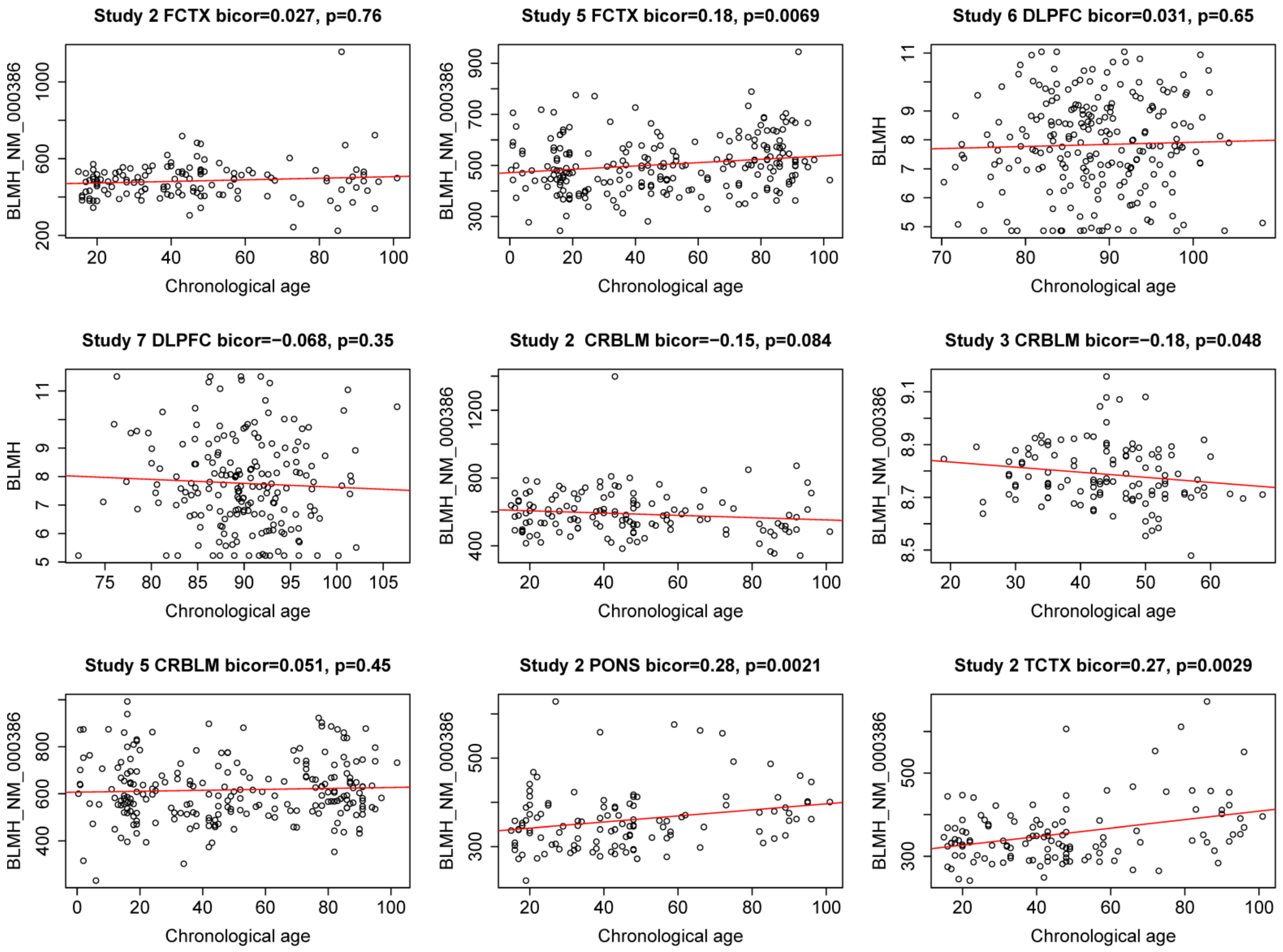
Study 2 FCTX bicor=0.027, p=0.76
Study 5 FCTX bicor=0.18, p=0.0069
Study 6 DLPFC bicor=0.031, p=0.65
Study 7 DLPFC bicor=−0.068, p=0.35
Study 2 CRBLM bicor=−0.15, p=0.084
Study 3 CRBLM bicor=−0.18, p=0.048
Study 5 CRBLM bicor=0.051, p=0.45
Study 2 PONS bicor=0.28, p=0.0021
Study 2 TCTX bicor=0.27, p=0.0029
BLMH_NM_000386
BLMH
Chronological age

b) CRLF3

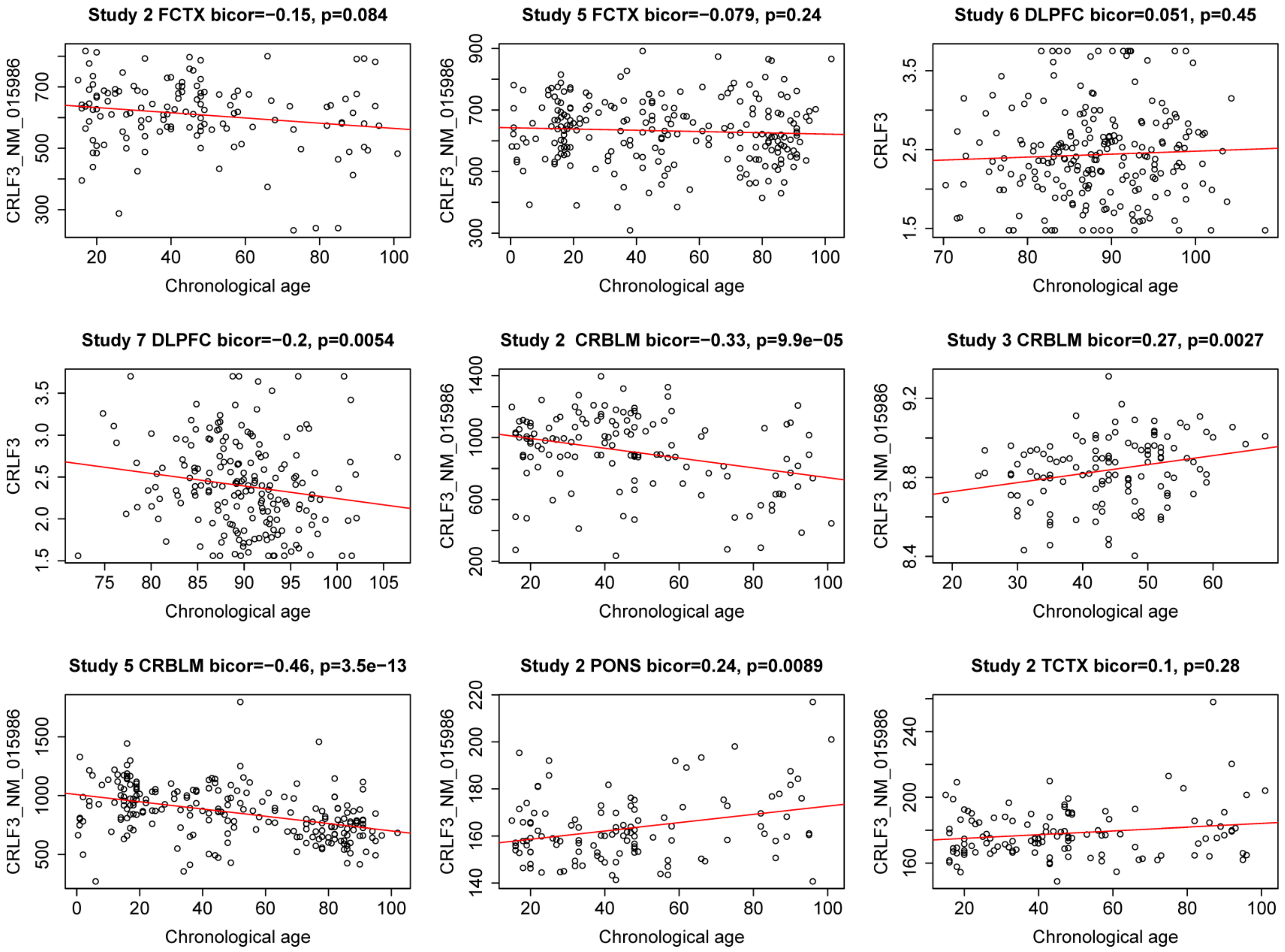
Study 2 FCTX bicor=−0.15, p=0.084
Study 5 FCTX bicor=−0.079, p=0.24
Study 6 DLPFC bicor=0.051, p=0.45
Study 7 DLPFC bicor=−0.2, p=0.0054
Study 2 CRBLM bicor=−0.33, p=9.9e−05
Study 3 CRBLM bicor=0.27, p=0.0027
Study 5 CRBLM bicor=−0.46, p=3.5e−13
Study 2 PONS bicor=0.24, p=0.0089
Study 2 TCTX bicor=0.1, p=0.28
CRLF3_NM_015986
CRLF3
Chronological age

c) EFCAB5

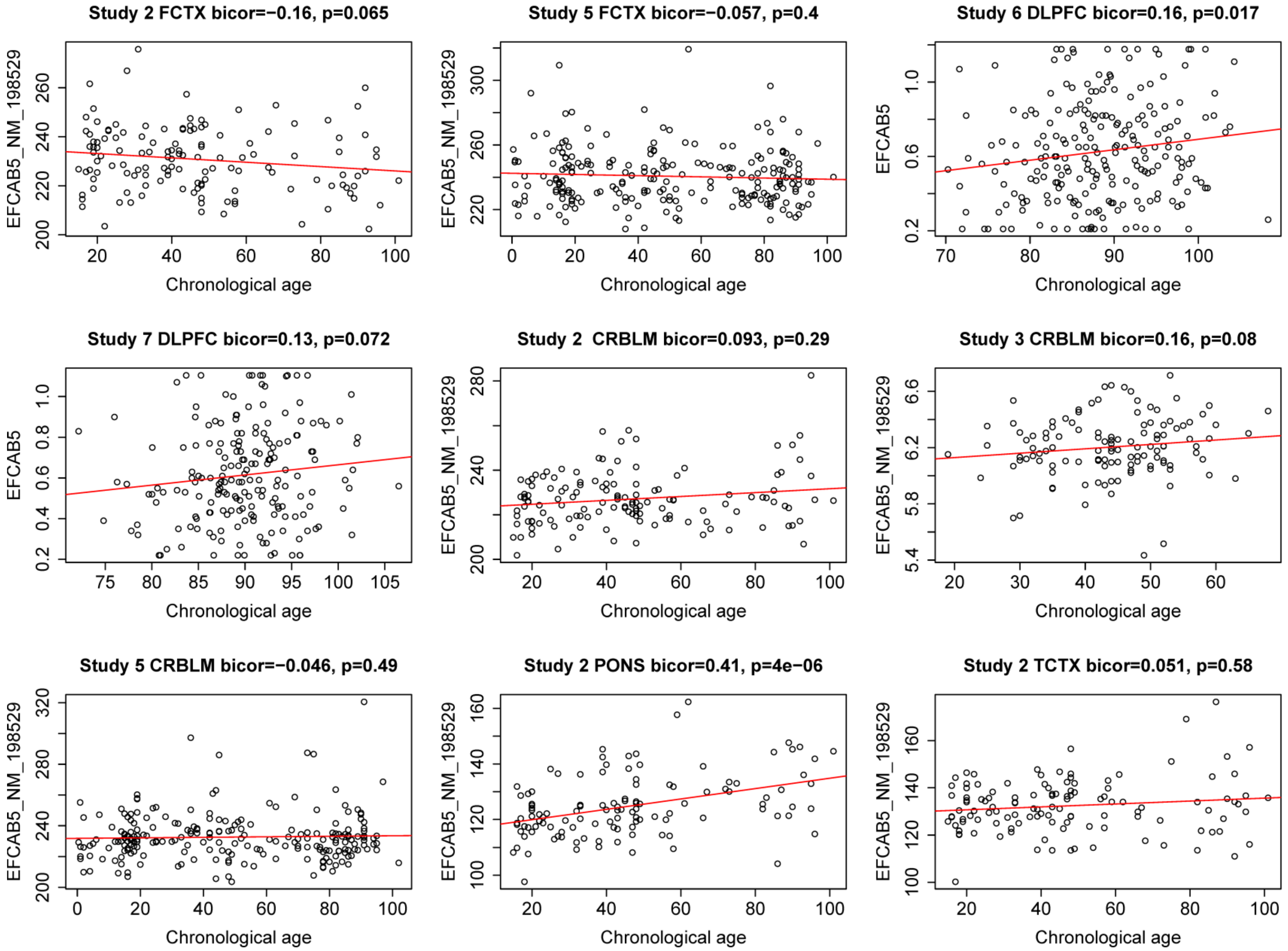

Study 2 FCTX bicor=−0.16, p=0.065
Study 5 FCTX bicor=−0.057, p=0.4
Study 6 DLPFC bicor=0.16, p=0.017
Study 7 DLPFC bicor=0.13, p=0.072
Study 2 CRBLM bicor=0.093, p=0.29
Study 3 CRBLM bicor=0.16, p=0.08
Study 5 CRBLM bicor=−0.046, p=0.49
Study 2 PONS bicor=0.41, p=4e−06
Study 2 TCTX bicor=0.051, p=0.58
EFCAB5_NM_198529
EFCAB5
Chronological age

d) GOSR1

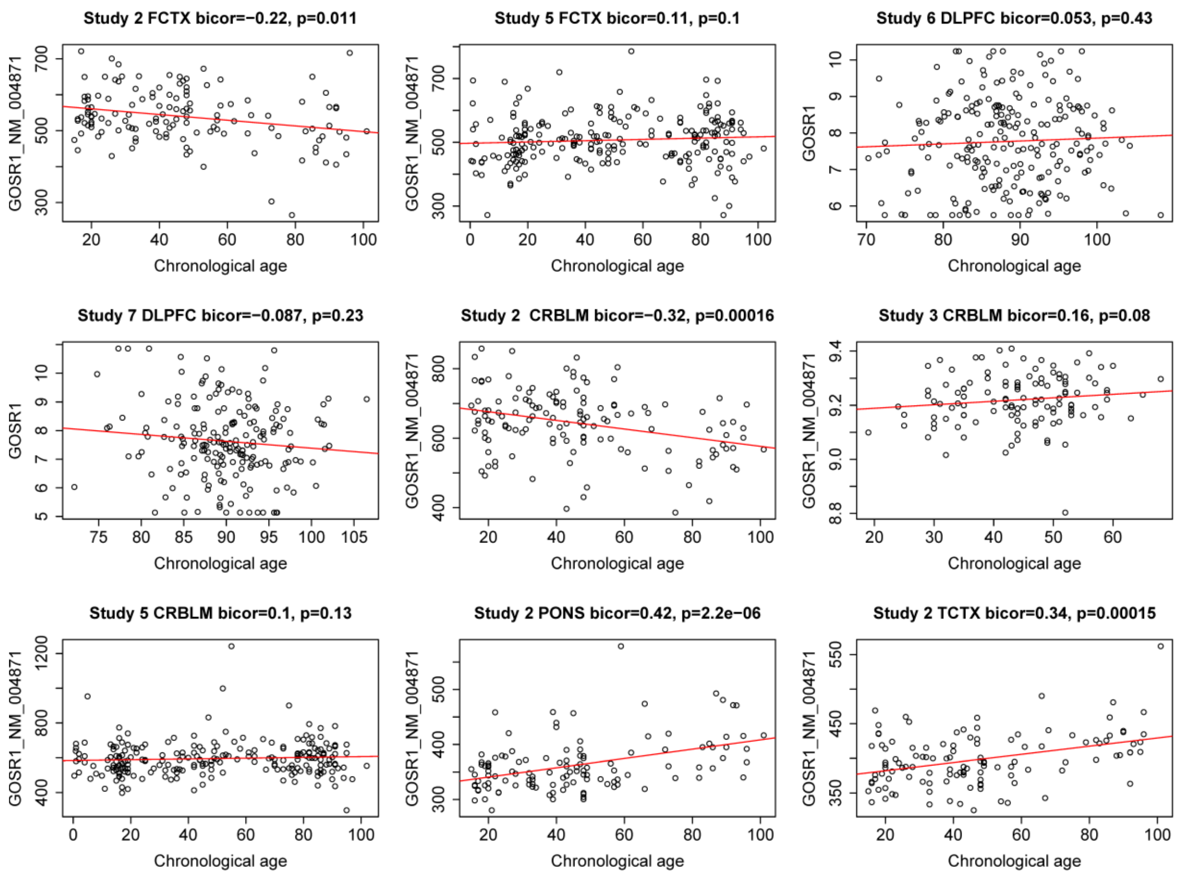

Supplementary Figure 12: Scatter plots of chronological age versus expression levels of genes in 17q11.2. The scatter plots relate the expression levels of a) *BLMH*, b) *CRLF3*, c) *EFCAB5* and d) *GOSR1* (y-axis) to chronological age (x-axis) in all brain data sets for which gene expression levels were available: study 2 (4 brain regions), study 3, study 5 (2 brain regions), study 6 and study 7. Each panel reports the results of a robust correlation (biweight midcorrelation estimation).

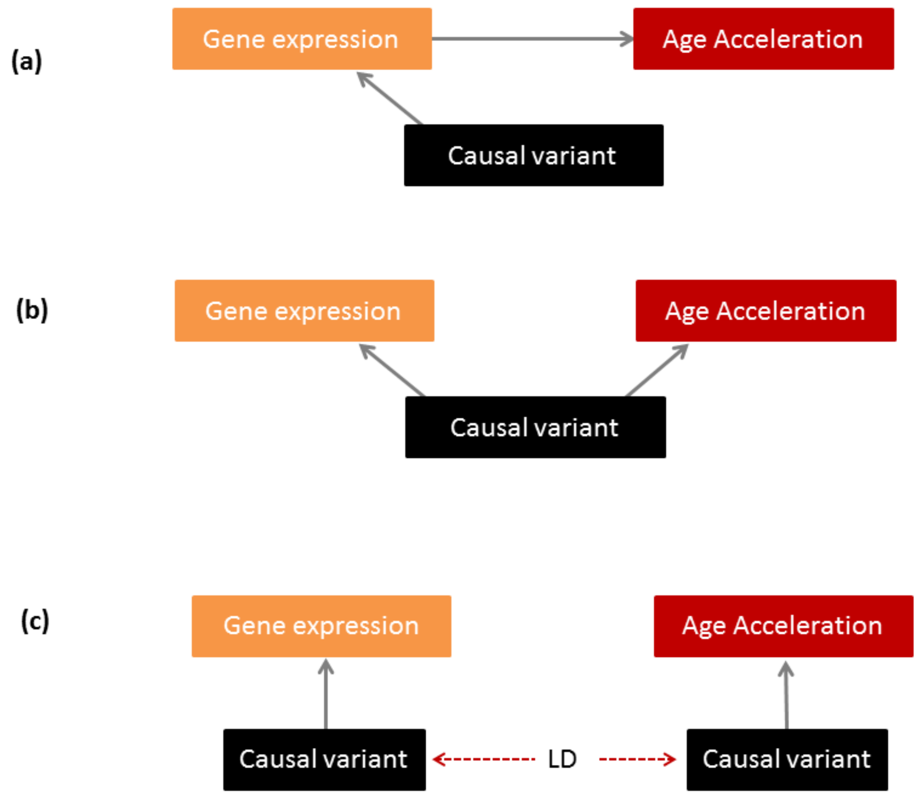

Supplementary Figure 13: Different causal scenarios evaluated by the HEIDI test. The diagrams depict the relationships between causal variants (e.g. a SNP), a gene expression trait, and the phenotype (age acceleration). The HEIDI test allows one to distinguish model (c) from the other two models. Thus, the models under the null hypothesis are (a) causal effect of gene expression on age acceleration or (b) pleiotropy. The causal model under the alternative hypothesis is (c) linkage, indicating linkage disequilibrium (LD) between two causal variants.

a)10q26

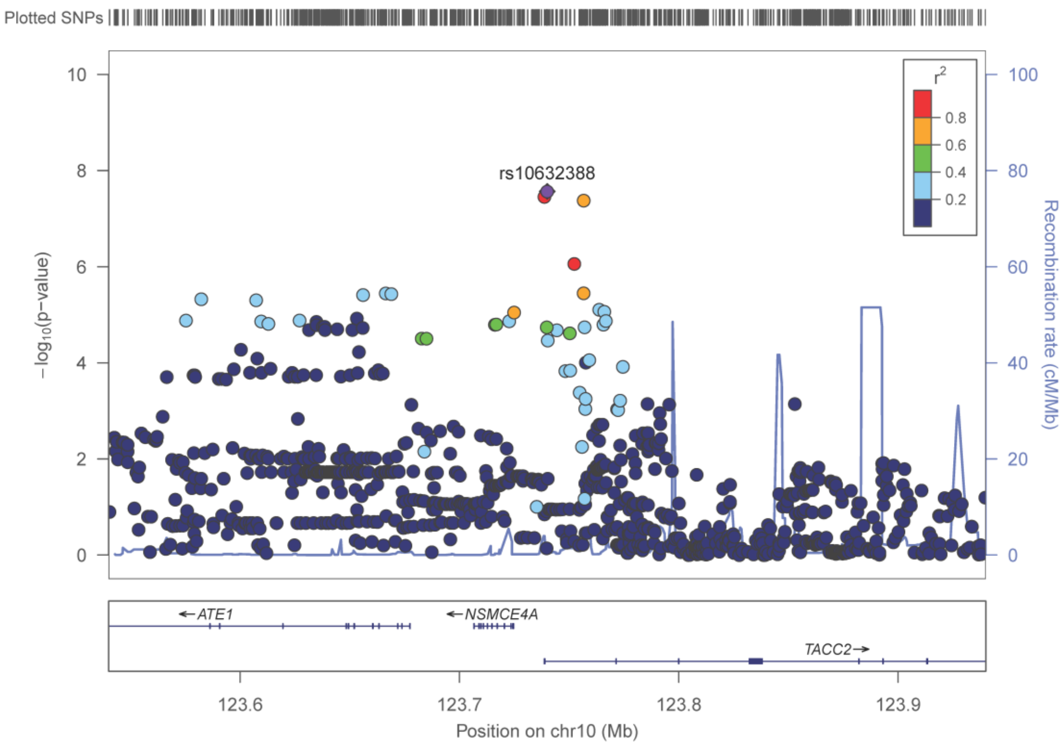

(b)12p13.31

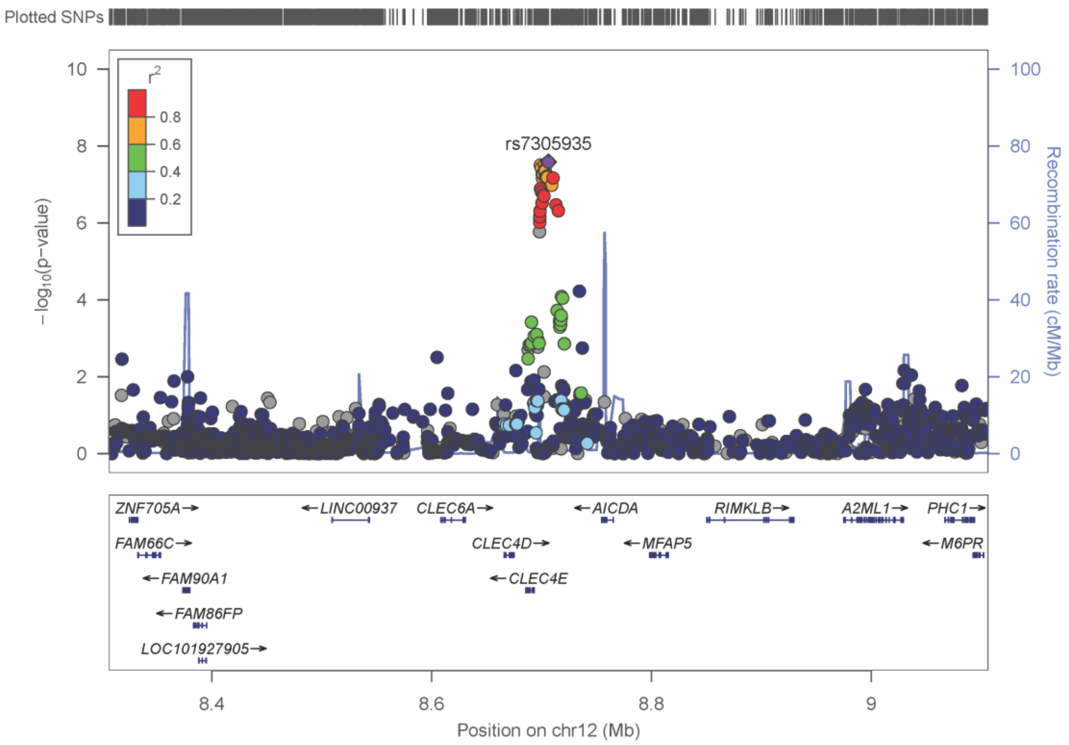

Supplementary Figure 14: Regional Manhattan plots for loci that affect the proportion of neurons in PFCTX . The plots display the association results surrounding the leading SNP of each genome-wide significant locus for the proportion of neurons in PFCTX: (a) 10q26, and (b) 12p13.31. The association P values resulted from the meta-analysis that combined GWAS from studies 4, 6 and 7. The colors visualize linkage disequilibrium (LD) $\boldsymbol{r^2}$ between the leading SNPs (colored in purple) and neighboring SNPs.

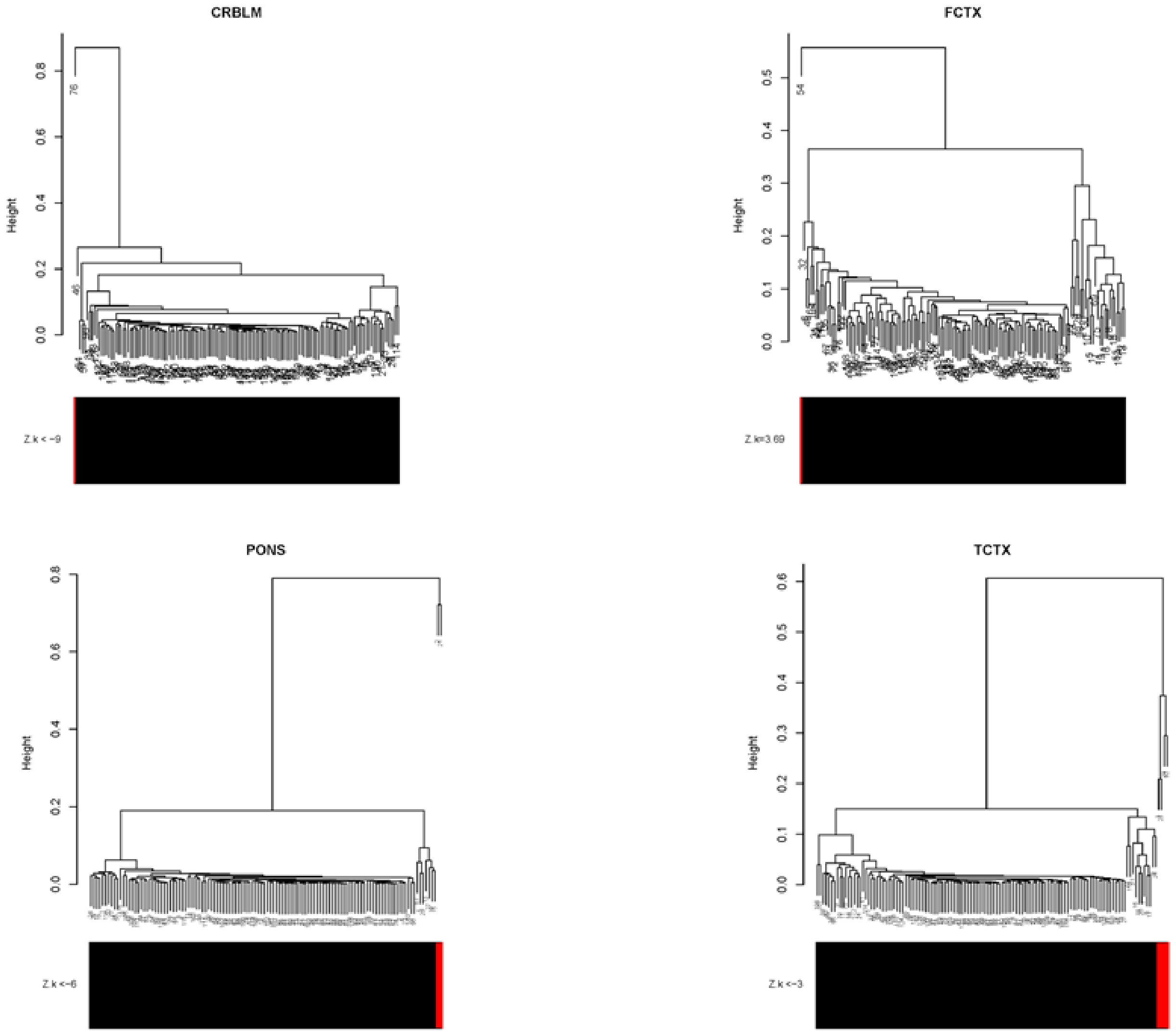
CRBLM
FCTX
PONS
TCTX
Height
Z.k < -9
Z.k=3.69
Z.k <-6
Z.k <-3

Supplementary Figure 15: Hierarchical clustering of the brain expression data from Study 2. These unsupervised hierachical clustering plots were used to identify outlying samples. The plots present four dendrograms based on mRNA expression profiled from CRBLM, frontal cortex (FCTX), pons (PONS) and temporal cortex (TCTX) tissues respectively. Expression data in CRBLM and FCTX were measured using Illumina HumanHT-12 V3.0 (m=48806) while PONS and TCTX were measured using Illumina humanRef-8 v2.0 (m=22184). The color band underneath the dendrogram represents standardized connectivity measures, $\boldsymbol{Z_k}$, (based on interarray correlation) with outliers colored in red. The outliers were removed from the cis-eQTL analysis.

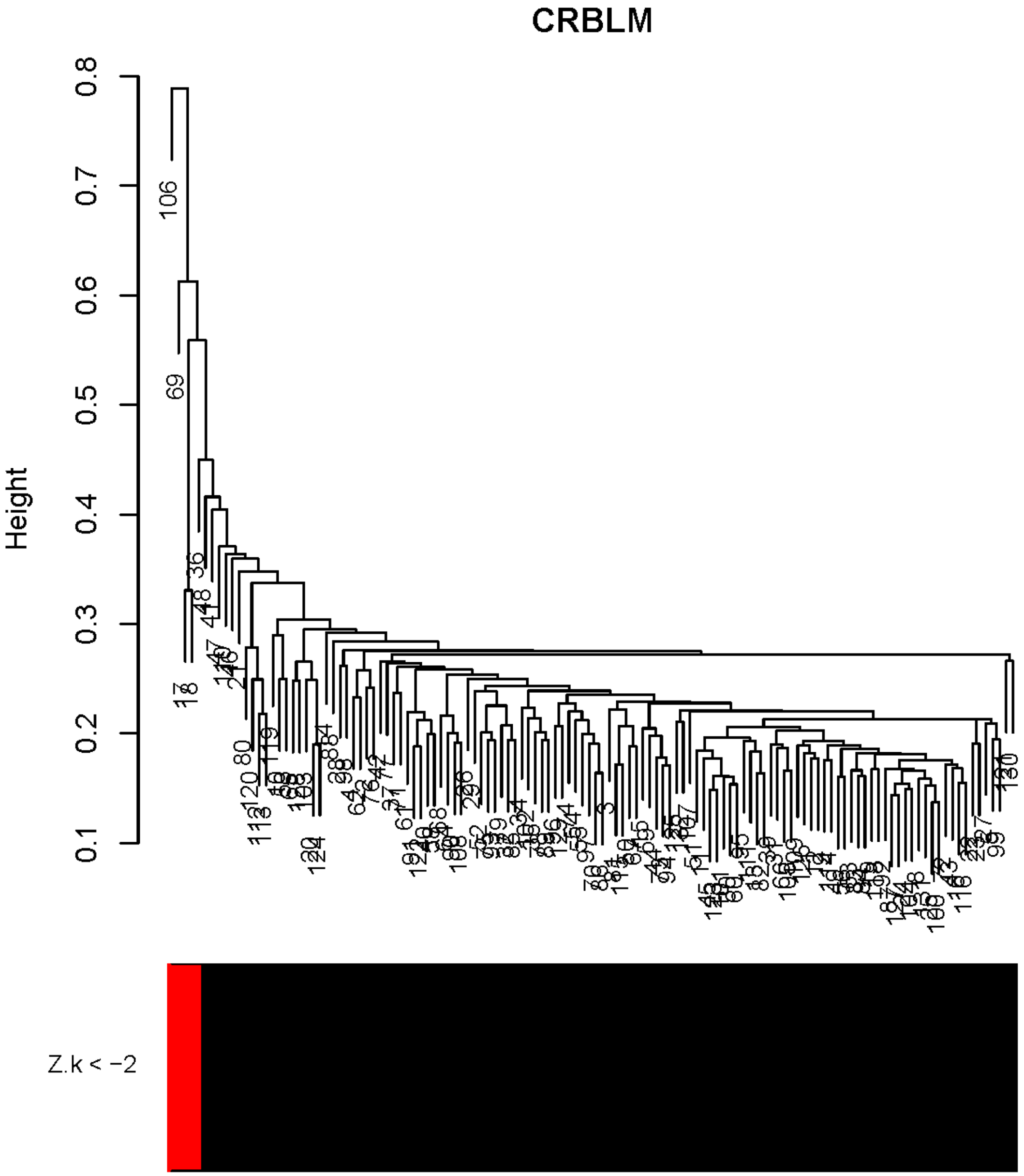

Supplementary Figure 16: Hierarchical clustering of the brain expression data from Study 3. Dendrogram based on mRNA expression profiled from the cerebellum. The color band underneath the dendrogram represents standardized connectivity measures, $\boldsymbol{Z_k}$, with outliers colored in red. The outliers were removed from the cis-eQTL analysis. The outliers were removed from the cis-eQTL analysis.

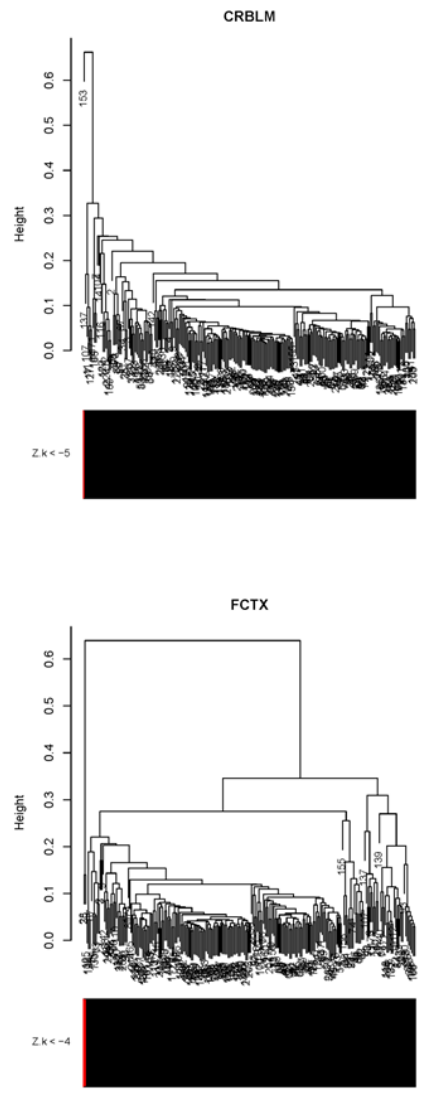

Supplementary Figure 17: Hierarchical clustering of the brain expression data from Study 5. The plots present dendrograms based on mRNA expression profiled from CRBLM (in the top) and FCTX (in the bottom), respectively. The color band underneath the dendrogram represents standardized connectivity measures, $\boldsymbol{Z_k}$, with outliers colored in red.

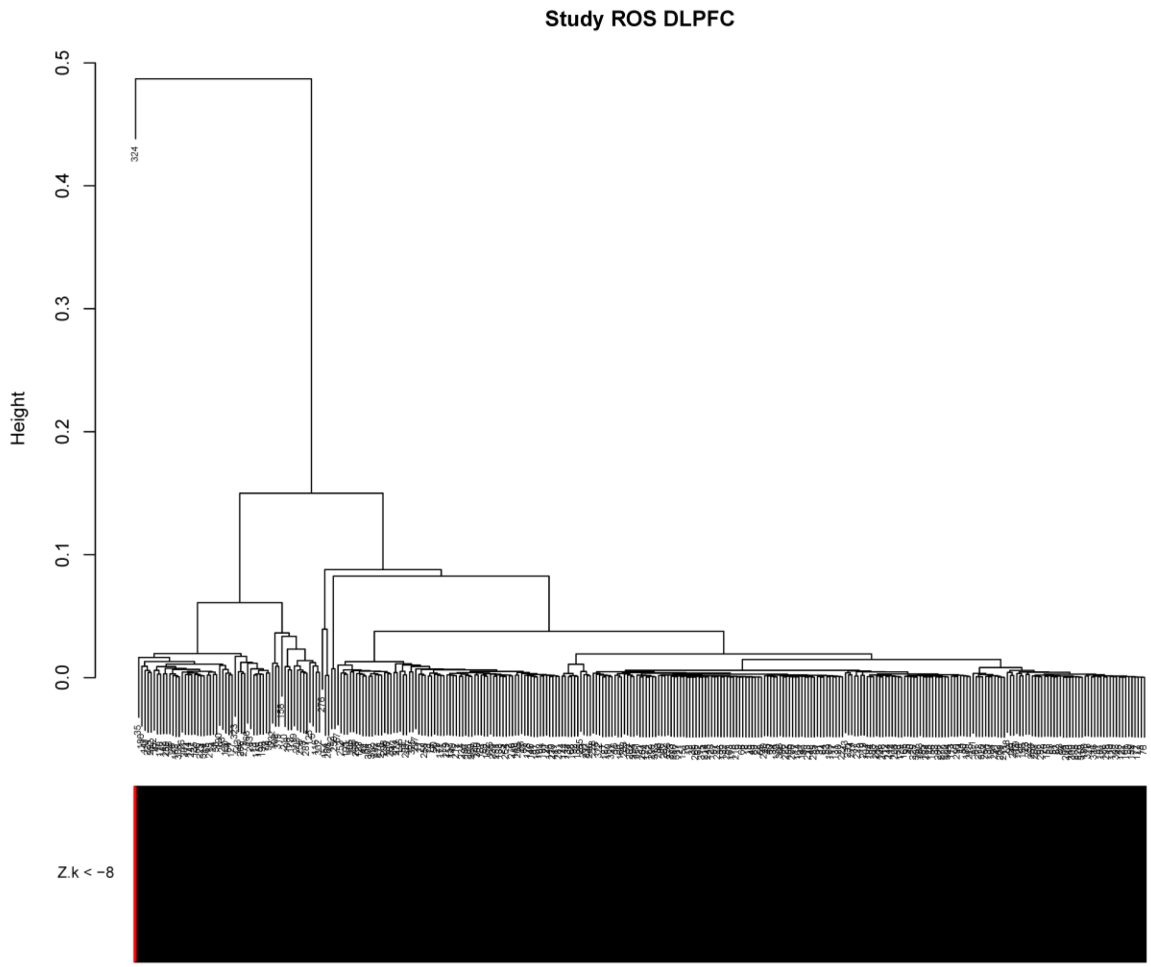

Supplementary Figure 18: Hierarchical clustering of the brain expression data from Study 6. Dendrogram based on mRNA expression profiled from dorsolateral prefrontal cortex (DLPFC). To identify outliers at subject level, we removed the probes of highly skewed distributions indicated by coefficient variation > 4, yielding 29625 probes remained for the hierarchical clustering analysis. The color band underneath the dendrogram represents standardized connectivity measures, $\boldsymbol{Z_k}$, with outliers colored in red.

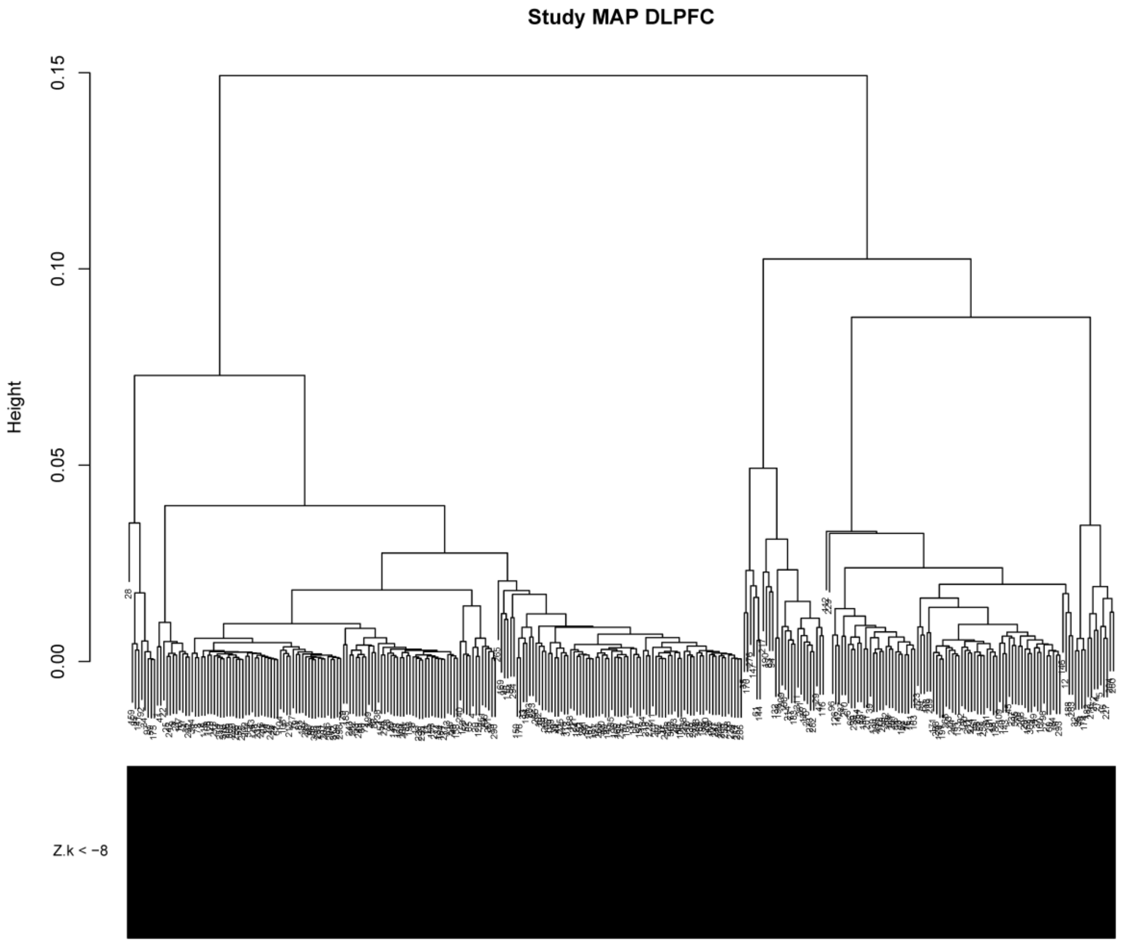

Supplementary Figure 19: Hierarchical clustering of the brain expression data from Study 7. The plots present dendrograms based on mRNA expression profiled from dorsolateral prefrontal cortex (DLPFC). To identify outliers at subject level, we removed the probes of highly skewed distributions indicated by coefficient variation > 4, yielding 29625 probes remained for the hierarchical clustering analysis. The color band underneath the dendrogram represents standardized connectivity measures, $\boldsymbol{Z_k}$, in which all subjects have $\boldsymbol{Z_k > -8}$ and are remained in *cis*-eQTL analysis.

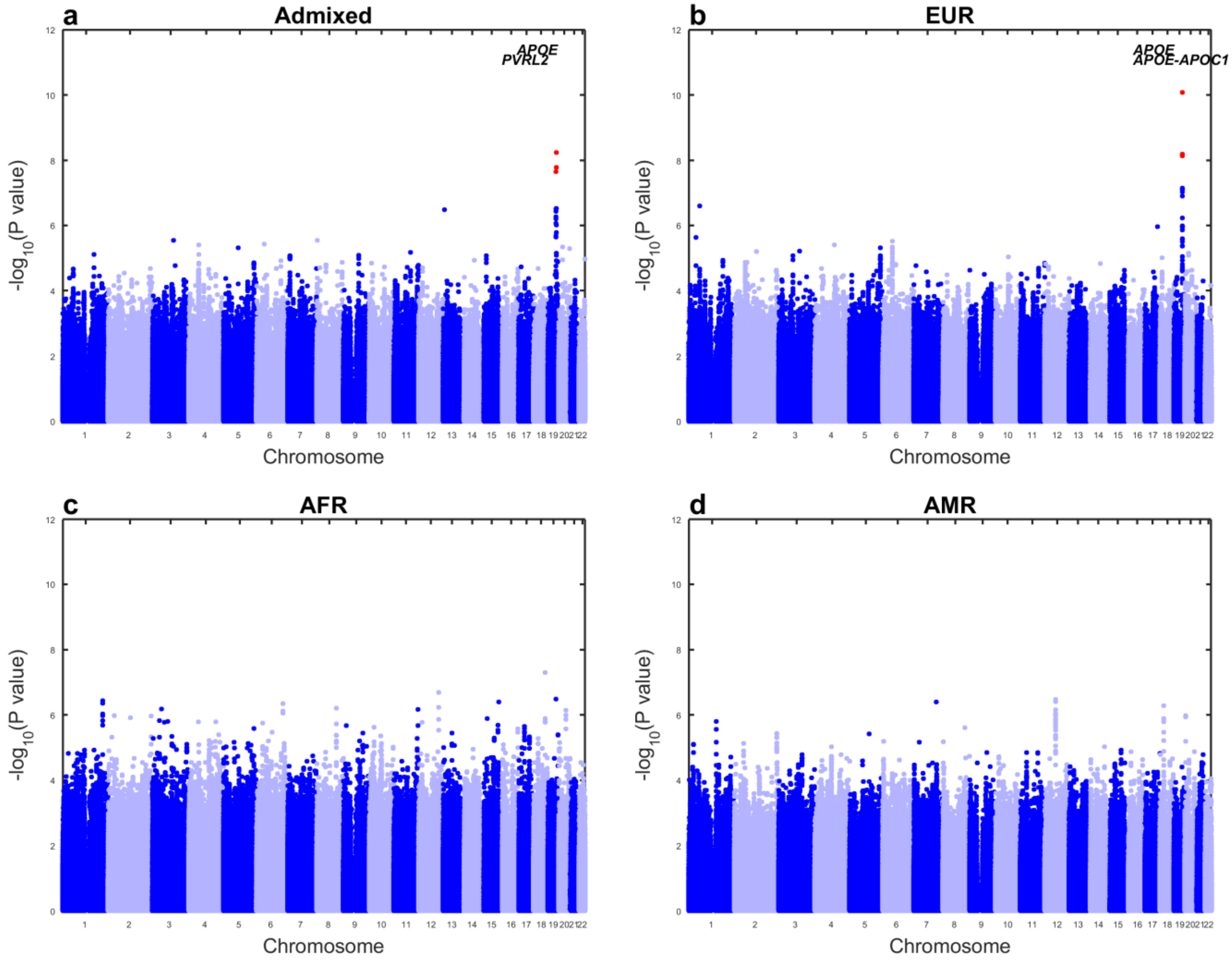

Supplementary Figure 20: Manhattan plot of a GWAS of cognitive decline/slope in the HRS. a) Manhattan plots for the GWAS for cognitive slope, performed on all participants of the Health and Retirement Study across all genetic ethnicity groups (Admixed). Analogous Manhattan plots for individuals of b) European (EUR), c) African (AFR), and d) Hispanic (AMR) ancestry. SNPs associated at $P < 5.0\text{x}10^{-8}$ are coded in red color, with neighboring gene names reported above significant loci.

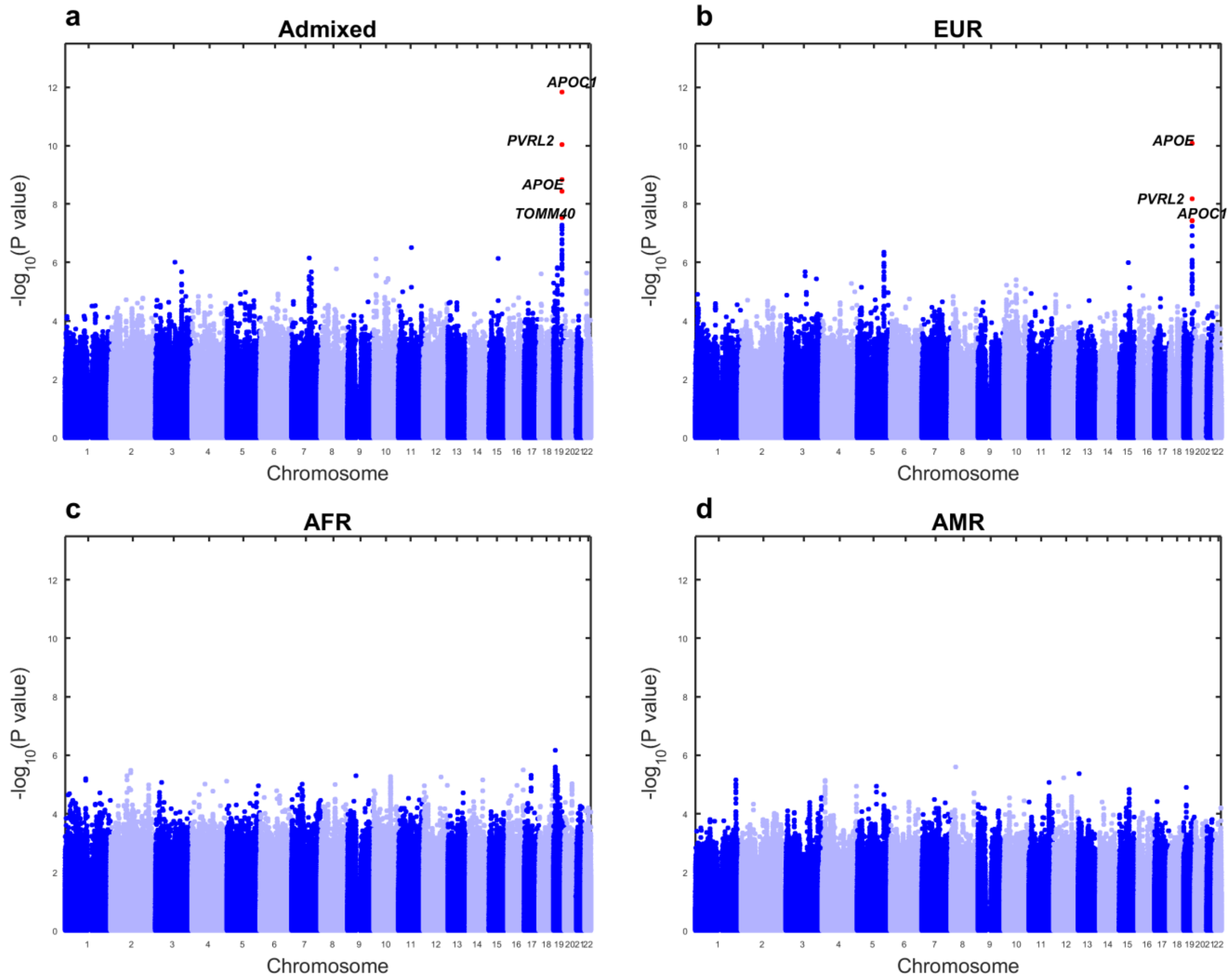

Supplementary Figure 21: Manhattan plot for the GWAS of dementia status in the HRS. The plot presents the Manhattan plots for the GWAS for dementia, performed on a) all the HRS participants across all genetic ethnicity groups (Admixed) as well as individuals of b) European (EUR), c) African (AFR) and d) Hispanic (AMR) ancestry. SNPs associated at $P < 5.0\text{x}10^{-8}$ are coded in red color, with their loci (gene names) listed in top.

Supplementary Tables

Data	SNP	DNA methylation	Gene expression
**Study 1**	Illumina 610-Quad,<br>Illumina 666W-Quad	Illumina 450K	Not available
**Study 2**	Illumina<br>HumanHap550v3	Illumina 27	Illumina<br>humanRef-8 v2.0<br>HumanHT-12 V3.0
**Study 3**	Affymetrix<br>SNP Array 5.0	Illumina 27	Affymetrix<br>Human Gene 1.0 ST Array
**Study 4**	Illumina<br>HumanOmniExpress	Illumina 450K	Not available
**Study 5**	Illumina 610-Quad	Illumina 27	Illumina<br>HumanHT-12 V3.0
**Studies 6 & 7**	Affymetrix SNP Array 6.0<br>Illumina HumanOmniExpress	Illumina 450K	Illumina<br>HiSeq

Supplementary Table 1: Genomic platforms in the different studies. The table present the platform types used for SNP, DNA methylation and gene expression array data in our study sets.

a)QC prior imputation

**Data**	**# SNPs**	**MAF**	**HWE** ***P***	**Missing rate threshold[a]**	**Genotyping rate**
**Study 1**	528739	≥ 1%	>1.0 x 10 $^{-4}$	3% (MAF > 5%)<br>1% (MAF > 1%)	>.97
**Study 2**	495169	≥ 5%	>1.0 x 10 $^{-4}$	0%[b]	>.99
**Study 3**	368899	≥ 5%	>1.0 x 10 $^{-4}$	30%	.97
**Study 4**	601993	≥ 5%	>1.0 x 10 $^{-4}$	30%	>.99
**Study 5**	515214	≥ 5%	>1.0 x 10 $^{-4}$	5%	>.99
**Study 6**	569364[c]<br>578503[d]	≥ 5%	>1.0 x 10 $^{-4}$	5%<br>5%	>.99<br>>.99
**Study 7**	569364[c]<br>578503[d]	≥ 5%	>1.0 x 10 $^{-4}$	5%<br>5%	>.99<br>>.99

b)QC post imputation and GWAS

**Data**	**Brain region**	λ_{GC}	**#SNPs**	**Ref. version**	**Imputation software**	**Info**	**genotype method[e]**	**MAF**	**# PCA**	**GWAS software**
**Study 1**	CRBLM<br>PFCTX	0.98<br>1.00	5552013	2012<br>Mar.	SHAPEIT/<br>IMPUTE2	0.4	Threshold at 0.80	≥ 5%	0	Plink
**Study 2**	CRBLM<br>FCTX<br>PONS<br>TCTX	1.03<br>1.02<br>1.00<br>1.04	6999923	2014<br>Oct.	SHAPEIT/<br>IMPUTE2	0.4	Expected dosage	≥ 5%	2	*R*
**Study 3**	CRLM	1.05	6926816	2014<br>Oct.	SHAPEIT/<br>IMPUTE2	0.4	Expected dosage	≥ 5%	2	*R*
**Study 4**	CRBLM<br>PFCTX	1.06<br>1.07	7172864	2014<br>Oct.	SHAPEIT/<br>IMPUTE2	0.4	Expected dosage	≥ 5%	0	*R*
**Study 5**	CRBLM<br>FCTX	1.02<br>1.00	6988010	2014<br>Oct.	SHAPEIT/<br>IMPUTE2	0.4	Expected dosage	≥ 5%	0	*R*
**Study 6**	DLPFC	1.00	6965786	2014<br>Oct.	SHAPEIT<br>IMPUTE2	0.4	Expected dosage	≥ 5%	0	*R*
**Study 7**	DLPFC	0.99	6965786	2014<br>Oct.	SHAPEIT<br>IMPUTE2	0.4	Expected dosage	≥ 5%	0	*R*

Abbreviations: CRBLM=cerebellum; DLPFC=dorsolateral prefrontal cortex; FCTX=frontal cortex; HWE=Hardy-Weinberg equilibrium; MAF= minor allele frequency; NA=not applicable; PCA= principal component; PFCTX=prefrontal cortex; PONS=pons; TCTX=temporal cortex; λ_{GC} = genomic inflation. Ref. version.=Dates of 1000 genome haplotypes released.

[a] SNPs failing the threshold were removed prior imputation.

[b] > 99.5 % markers have missing rates < 10%.

[c] Affymetrix Snp 6.0 Array.

[d] Illumina HumanOmniExpress. [e] HWE *P* > 1.0 x 10^{-4} was required and minimum number of samples per marker level was requested ≥20 .

Supplementary Table 2: SNPs used for imputation and GWAS. The tables presents the guidelines for quality controls for SNP array data (a) prior imputation and (b) posterior imputation, as well as information for GWAS analysis.

Study	Covariate	β	SE	t-statistic	P
**Study 2**					
	Intercept	31.56	4.93	6.40	1.0E-08
	Age	0.55	0.06	8.75	3.0E-13
	Female	-1.21	1.11	-1.08	2.8E-01
	AD	0.92	1.24	0.74	4.6E-01
	propN	-6.50	5.34	-1.22	2.3E-01
**Study 6**					
	Intercept	28.27	2.86	9.89	4.0E-20
	Age	0.54	0.03	18.18	3.4E-50
	Female	-0.57	0.49	-1.16	2.5E-01
	AD	0.44	0.43	1.04	3.0E-01
	propN	-21.51	3.69	-5.82	1.5E-08
**Study 7**					
	Intercept	33.34	3.36	9.92	7.4E-20
	Age	0.50	0.04	13.70	1.9E-32
	Female	-1.30	0.48	-2.69	7.6E-03
	AD	1.18	0.43	2.76	**6.3E-03**
	propN	-23.70	3.52	-6.73	1.1E-10

Supplementary Table 3: Multivariate regression model analysis for showing that AD status is associated with epigenetic age acceleration in the prefrontal cortex. The table presents multivariate linear regression models of epigenetic age acceleration in PFCTX (dependent variable). We regressed DNAmAge on chronological age, gender, Alzheimer's disease (AD) status, and the proportion of neurons (propN). We performed the regression analysis in each of the three studies (2, 6, and 7) that included AD cases and controls. The results for AD status were combined using a fixed effects model weighted by inverse variance. The meta-analysis shows that epigenetic age acceleration is increased with AD status by 0.82 years (SE=0.30 and P=5.7x10-3). Note that AD status has consistent directions across all the three studies, of which study 7 reveals the strongest association (P=6.3x10-3).

Data	Brain Region	Chronological age	Gender	propN	Disease status
**Study 1**	CRBLM<br>PFCTX	yes<br>yes	no<br>no	no<br>no	no<br>no
**Study 2**	CRBLM<br>FCTX<br>PONS<br>TCTX	yes<br>yes<br>yes<br>yes	no<br>no<br>no<br>no	no<br>yes<br>no<br>yes	no<br>no<br>no<br>no
**Study 3**	CRLM	yes	no	no	no
**Study 4**	CRBLM<br>PFCTX	yes<br>yes	no<br>no	no<br>yes	maybe<br>maybe
**Study 5**	CRBLM<br>FCTX	yes<br>yes	no<br>no	no<br>no	no<br>no
**Study 6**	DLPFC	yes	yes	yes	yes
**Study 7**	DLPFC	yes	yes	yes	yes

Supplementary Table 4: Effect of age, gender, and proportion of neurons on DNAm age. The entries of the table indicate whether a given variable (columns) had a significant effect on DNAm age. Each row corresponds to a different study/brain region. This table was used to determine which covariate could have a confounding effect on epigenetic age acceleration. Our intrinsic measure of age acceleration adjusted for each covariate that is marked by "yes".

				CRBLM		FCTX		PFCTX		PONS		TCTX		ALL	
**Chr**	**SNP**	**Gene**	**Minor/ Major alleles**	**Corr. (SE)**	**Meta *P***	**Corr. (SE)**	**Meta *P***	**Corr. (SE)**	**Meta *P***	**Corr. (SE)**	**Meta *P***	**Corr. (SE)**	**Meta *P***	**Corr. (SE)**	**Meta *P***
2	rs6723868	*DHX57*	A/G	0.23 (0.04)	3.1×10^{-8}	0.03 (0.03)	0.6	0.06 (0.04)	0.2	0.15 (0.09)	0.09	0.21 (0.09)	2.0×10^{-2}	0.12 (0.03)	5.0×10^{-6}
16	rs30986	near *MLST8*	T/C	0.25 (0.04)	9.3×10^{-9}	0.13 (0.04)	0.01	-0.04 (0.04)	0.4	0.19 (0.09)	2.6×10^{-2}	0.12 (0.09)	0.2	0.09 (0.03)	2.1×10^{-3}

Chr=chromosome;
Corr.= Correlation with respect to minor allele.

Supplementary Table 5: Evaluating whether published SNPs that affect epigenetic age acceleration in the cerebellum also have a significant effect on age acceleration in independent data sets. The table presents the meta-analysis association results for the two SNPs identified in our earlier GWAS of cerebellar age acceleration[3]. The first macro column "CRBLM" lists the association results from our previous study. The 2nd to 5th macro column list the results from our current study using the following brain regions: FCTX, PFCTX, PONS, and TCTX. The last macro column "ALL" lists the results using the tissues from all the test brain regions.

**Brain tissue**	**Number of RNA-seq and genotyped samples**	**Number of RNA-seq Samples**
Amygdala	62	72
Anterior Cingulate Cortex	72	84
Caudate Basal Ganglia	100	117
Cerebellar Hemisphere	89	105
Cerebellum	103	125
Cortex	96	114
Frontal Cortex	92	108
Hippocampus	81	94
Hypothalamus	81	96
Nucleus Accumbens	93	113
Putamen	82	97
Substantia Nigra	56	63

Original results were downloaded from http://www.gtexportal.org/home/tissueSummaryPage.

Supplementary Table 6: Sample size distribution in the GTEx brain atlas used for brain *cis*-eQTL analysis. The table presents tissue counts stratified by 12 brain regions released from the data summary in the Genotype-Tissue Expression (GTEx) project[4]. In the latest released version (V6), there were 1,007 brain samples from up to 449 individuals.

Gene	Data	region	Probe ID	Bicor	SE	*P*	N	$\alpha_{Bonferroni}$
BLMH	Study 2	FCTX	ILMN_1721921	-0.05	0.09	5.93E-01	40	1.25E-03
	Study 5	FCTX	ILMN_1721921	-0.01	0.07	8.54E-01	40	1.25E-03
	Study 6	DLPFC	ENSG00000108578.10	0.28	0.06	**3.09E-06**	73	6.85E-04
	Study 7	DLPFC	ENSG00000108578.10	0.18	0.06	4.98E-03	73	6.85E-04
	Study 2	CRBLM	ILMN_1721921	-0.07	0.09	3.91E-01	40	1.25E-03
	Study 3	CRBLM	X8014008	0.13	0.09	1.53E-01	27	1.85E-03
	Study 5	CRBLM	ILMN_1721921	0.10	0.07	1.54E-01	40	1.25E-03
	Study 2	PONS	ILMN_1721921	0.01	0.09	9.11E-01	28	1.79E-03
	Study 2	TCTX	ILMN_1721921	0.06	0.09	4.99E-01	28	1.79E-03
CRLF3	Study 2	FCTX	ILMN_2155228	0.13	0.09	1.68E-01	40	1.25E-03
	Study 5	FCTX	ILMN_2155228	0.18	0.07	7.17E-03	40	1.25E-03
	Study 6	DLPFC	ENSG00000176390.10	0.12	0.06	3.93E-02	73	6.85E-04
	Study 7	DLPFC	ENSG00000176390.10	-0.01	0.07	8.94E-01	73	6.85E-04
	Study 2	CRBLM	ILMN_2155228	0.05	0.09	5.77E-01	40	1.25E-03
	Study 3	CRBLM	X8014037	0.28	0.09	**1.58E-03**	27	1.85E-03
	Study 5	CRBLM	ILMN_2155228	0.13	0.07	5.77E-02	40	1.25E-03
	Study 2	PONS	ILMN_1660579	-0.12	0.09	2.07E-01	28	1.79E-03
	Study 2	TCTX	ILMN_1660579	-0.14	0.09	1.36E-01	28	1.79E-03
EFCAB5	Study 2	FCTX	ILMN_1788305	0.04	0.09	6.55E-01	40	1.25E-03
	Study 5	FCTX	ILMN_1788305	0.02	0.07	7.85E-01	40	1.25E-03
	Study 6	DLPFC	ENSG00000176927.10	0.05	0.06	3.91E-01	73	6.85E-04
	Study 7	DLPFC	ENSG00000176927.10	0.15	0.07	2.49E-02	73	6.85E-04
	Study 2	CRBLM	ILMN_1788305	0.05	0.09	5.46E-01	40	1.25E-03
	Study 3	CRBLM	X8006085	0.37	0.09	**2.30E-05**	27	1.85E-03
	Study 5	CRBLM	ILMN_1788305	-0.01	0.07	9.21E-01	40	1.25E-03
	Study 2	PONS	ILMN_1788305	0.05	0.09	6.16E-01	28	1.79E-03
	Study 2	TCTX	ILMN_1788305	0.09	0.09	3.29E-01	28	1.79E-03
GOSR1	Study 2	FCTX	ILMN_1798816	-0.15	0.09	1.05E-01	40	1.25E-03
	Study 5	FCTX	ILMN_1798816	0.01	0.07	8.80E-01	40	1.25E-03
	Study 6	DLPFC	ENSG00000108587.10	-0.11	0.06	7.40E-02	73	6.85E-04
	Study 7	DLPFC	ENSG00000108587.10	-0.27	0.06	**2.43E-05**	73	6.85E-04
	Study 2	CRBLM	ILMN_1798816	-0.07	0.09	4.26E-01	40	1.25E-03
	Study 3	CRBLM	X8006148	0.13	0.09	1.42E-01	27	1.85E-03
	Study 5	CRBLM	ILMN_1798816	0.07	0.07	3.09E-01	40	1.25E-03
	Study 2	PONS	ILMN_1712631	-0.18	0.09	4.97E-02	28	1.79E-03
	Study 2	TCTX	ILMN_1712631	-0.03	0.09	7.23E-01	28	1.79E-03

Supplementary Table 7: Cis-expression QTL analysis of SNPs associated with epigenetic age acceleration in human brain. We conducted *cis*-eQTL analysis based on three categories of brain expression data, as listed in **Methods** in the main article. We present the results using the expression data from category one (individual level data) in which we identified genes exhibiting *cis*-effects, referred as to step (1) in **Methods**. Category one involved the gene expression data from our study sets 2, 3, 5, 6 and 7 that allowed us to correlate our leading SNPs on susceptibility loci with brain gene expression levels of adjacent genes (i.e. genes that are located within +/- 1Mb of the test SNPs).

For each study and brain region, we examined *cis*-effects and highlighted the genes surpassing the Bonferroni corrected significance level $\alpha_{Bonferroni}$. The highlighted genes (colored in red) were subsequently analyzed using the brain expression data from category 2 (GTEx) and category 3 (the UK database). As a result, there are four *cis*-genes identified in 17q11.2. Each row corresponds to a study/brain region. The columns report the biweight midcorrelation "Bicor", standard error "SE" and the corresponding nominal p-value "P". The column "N" lists the number of (SNP, gene expression) pairs tested. The significance level (corrected for the number N of multiple comparisons) of each brain region can be found in the last column "$\alpha_{Bonferroni}$". The P values are colored in bold red if they were smaller than $\alpha_{Bonferroni}$.

.

Gene	Ensembl gene ID	P	Effect Size	Tissue
ANKRD13B	ENSG00000198720.8	9.70E-09	2.40E-01	Thyroid
BLMH	ENSG00000108578.10	1.30E-08	2.60E-01	Artery - Tibial
CORO6	ENSG00000167549.14	5.30E-28	7.30E-01	Thyroid
CRYBA1	ENSG00000108255.3	8.60E-06	-5.70E-01	Esophagus - Gastroesophageal Junction
EFCAB5	ENSG00000176927.10	3.40E-11	4.80E-01	Thyroid
EFCAB5	ENSG00000176927.10	2.00E-08	4.30E-01	Cells - Transformed fibroblasts
EFCAB5	ENSG00000176927.10	2.80E-07	3.10E-01	Colon - Transverse
EFCAB5	ENSG00000176927.10	1.40E-06	3.40E-01	Nerve - Tibial
EFCAB5	ENSG00000176927.10	3.00E-06	4.40E-01	Brain - Cerebellum
EFCAB5	ENSG00000176927.10	1.50E-05	3.00E-01	Skin - Sun Exposed (Lower leg)
GIT1	ENSG00000108262.11	4.50E-08	2.90E-01	Thyroid
RP11-68I3.10	ENSG00000264007.1	6.00E-17	5.60E-01	Thyroid
SLC6A4	ENSG00000108576.5	3.00E-05	-3.10E-01	Nerve - Tibial
SSH2	ENSG00000141298.13	2.00E-11	3.50E-01	Thyroid
SSH2	ENSG00000141298.13	1.90E-08	2.20E-01	Lung
SSH2	ENSG00000141298.13	7.20E-08	1.90E-01	Nerve - Tibial
SSH2	ENSG00000141298.13	1.60E-05	8.10E-02	Whole Blood
TP53I13	ENSG00000167543.11	2.50E-07	1.80E-01	Thyroid

The table contents are sorted by gene.
Original results were downloaded from http://www.gtexportal.org/home/documentationPage#AboutGTEx.

Supplementary Table 8: Significant GTEx *cis*-eQTL for chromosome 17 rs2054847 across human tissue types.

The table presents a total of 18 significant *cis*-eQTL analysis results for chromosome 17 rs2054847 reported from GTEx V6. In the V6 version, GTEx reports *cis*-eQTL results that were significant after multiple corrections. For each gene, a permutation test was used to determine a threshold that corrects for multiple comparisons across genes and tissue types. Nominal P values are reported in the colum "*P*". The 18 *cis*-eQTL results (rows) correspond to 10 distinct gene symbols (and ensemble gene identifiers) in 10 human tissue types.

		Our study				GTEx				UK			
**Gene**	**Region**	**β**	$\boldsymbol{P_{SMR}}$	$\boldsymbol{P_{HEIDI}}$	$\boldsymbol{N_{SNP}}$	**β**	$\boldsymbol{P_{SMR}}$	$\boldsymbol{P_{HEIDI}}$	$\boldsymbol{N_{SNP}}$	**β**	$\boldsymbol{P_{SMR}}$	$\boldsymbol{P_{HEIDI}}$	$\boldsymbol{N_{SNP}}$
BLMH	CRBLM	--	--	--	--	-0.45	2.1E-01	--	--	--	--	--	--
	PFCTX[a]	-0.09	7.8E-01	2.4E-04	631	--	--	--	--	--	--	--	--
CRLF3	CRBLM[b]	-5.4	**1.5E-02**	0.09	315	-0.48	2.1E-01	0.96	6	--	--	--	--
	FCTX[c]	-0.003	3.0E-01	8.6E-02	666	-0.27	3.2E-01	0.22	132	--	--	--	--
	PFCTX[a]	-0.88	2.0E-01	0.09	341	--	--	--	--	--	--	--	--
EFCAB5	CRBLM [b]	-6.20	**3.4E-04**	0.9	485	-1.73	**7.1E-05**	0.85	305	-5.71	**1.2E-04**	0.15	437
	FCTX	--	--	--	--	-1.75	**3.0E-03**	0.09	22	-7.88	**3.9E-04**	0.03	556
	PFCTX[a]	-14.0	**9.2E-03**	0.8	8	--	--	--	--	--	--	--	--
	TCTX	--	--	--	--	--	--	--	--	-6.93	**2.9E-04**	0.15	476
	ALL[e]	--	--	--	--	--	--	--	--	-9.57	**1.8E-05**	0.54	496
GOSR1	FCTX	--	--	--	--	0.43	3.2E-01	0.29	1	--	--	--	--
	PFCTX[a]	0.60	1.5E-01	0.1	845	--	--	--	--	--	--	--	--
	PONS[d*]	0.02	0.09	0.4	37	--	--	--	--	--	--	--	--
	ALL[e]	--	--	--	--	--	--	--	--	2.55	1.8E-01	-1.00	--

$\boldsymbol{P_{SMR}} \leq 0.05$ marked in bold.
-- denoted not available.
* There was no eQTL result with P less than the defined threshold 1.57E-03. Thus, the threshold was set at 0.01 in order to perform the SMR and HEIDEI tests.
[a] the analysis result from our study was performed on a combined sample studies 6 & 7;
[b] the analysis result from our study was performed on study 3; [c] the analysis result from our study was performed on study 5; [d] the analysis result from our study was performed on study 2; [e] denote the expression averaged on all the 10 brain regions in the UK database.

Supplementary Table 9: SMR and HEIDI analyses for *cis*-genes identified in 17q11.2. Results from a summary data-based Mendelian randomization analysis (SMR) in conjunction with heterogeneity in dependent instruments (HEIDI) analysis. SMR and HEIDI were performed using our GWAS results surrounding the 4 genes BLMH, CRLF3, EFCAB5 and GOSR1 and the corresponding cis-eQTL results from (1) our individual level data, (2) GTEx and (3) BRAINEAC (denoted by UK). The SMR test yields a slope estimate β for the change in epigenetic age acceleration per unit expression and the associated P value (PsMR). The HEIDI test yields a P value (PHEIDI) for interpreting the association between expression and age acceleration, where nonsignificant PHEIDI (≥ 0.01) are desirable because they suggest the test expression and age acceleration are affected by the same causal variants. For each macro column, we list the SMR results: β and PsMR and the HEIDI results: PHEIDI and NSNP for the number of SNPs used in HEIDI.

				75th		95th	
**Brain Region**	**Data base**	**Pathways/Gene set**	**size**	**Nominal *P*MAGENTA**	**FDR**	**Nominal *P*MAGENTA**	**FDR**
**ALL**	KEGG	Drug metabolism cytochrome P450	51	3.30E-03	**0.23**	1.11E-01	1
	KEGG	MAPK signaling pathway	248	4.00E-03	**0.16**	2.30E-02	1
	KEGG	Vibrio cholerae infection	52	4.10E-03	**0.17**	4.81E-01	1
	Ingenuity	Insulin receptor signaling	34	3.50E-03	**0.20**	5.14E-01	1
	PB	Fatty acid beta-oxidation	23	4.60E-03	**0.23**	2.52E-02	0.4
**PFCTX**	KEGG	Neuroactive ligand receptor interaction	239	5.17E-02	0.76	1.44E-04	**0.08**
	GO	G-protein coupled receptor activity	306	2.55E-02	0.84	1.00E-04	**0.2**
	GO	GTPase inhibitor activity	11	2.00E-04	**0.047**	9.85E-02	1
	Ingenuity	Role of BRCA1 in DNA damage response	29	2.20E-03	**0.07**	5.11E-02	1

PB=PANTHER biological process.
FDR ≤ 0.25 marked in bold.
*resulted from the MAGENTA algorithm.

Supplementary Table 10: Functional enrichment study of SNP sets associated with epigenetic brain aging. Here we used the MAGENTA software to evaluate what is known about the set of SNPs that is associated with epigenetic age acceleration in (1) all the test brain regions (ALL) and (2) prefrontal cortex (PFCTX). The analysis is based on the *P* values from the meta-analysis GWAS results. The cutoffs of gene set enrichment analysis (GSEA) in the MAGENTA algorithm were set at 95th and 75th percentiles. We listed the results corresponding to the most significant results (at a FDR < 0.25 in either cutoffs) from the MAGENTA analysis.

a) **Results for ALL**

GWAS	POP	Gender	Hypergeometric P	FDR	#genes overlap /annotation
**(a) Neurodegenerative disorder**					
AMD	EUR+ASN	M & F	8.5E-01	> 0.99	9/478
AMD Geographic Atrophy	EUR+ASN	M & F	6.5E-01	> 0.99	11/478
AMD Neovascular	EUR+ASN	M & F	9.9E-01	> 0.99	5/478
ALZ stage 1	EUR	M & F	7.7E-01	> 0.99	10/482
ALZ	EUR	M & F	> 0.99	> 0.99	0/50
Age at Huntington's disease motor onset	EUR	M & F	9.8E-01	> 0.99	6/478
Parkinson’s disease	EUR	M & F	7.5E-01	> 0.99	10/476
**(b) Neuropsychiatric disorder**					
ADHD	Admixed	M & F	> 0.99	> 0.99	4/439
BIP	Admixed	M & F	9.6E-01	> 0.99	6/439
MDD	EUR	M & F	9.6E-01	> 0.99	6/430
Schizophrenia	Admixed	M & F	9.2E-01	> 0.99	8/483
**(c) Cognitive functioning from HRS study**					
Cognitive slope	Admixed	M & F	1.6E-01	> 0.99	16/485
	EUR	M & F	3.3E-01	> 0.99	14/485
	AFR	M & F	3.3E-01	> 0.99	14/485
	AMR	M & F	8.6E-01	> 0.99	9/485
Dementia	Admixed	M & F	5.5E-01	> 0.99	12/485
	EUR	M & F	2.0E-02	6.50E-01	20/485
	AFR	M & F	5.4E-04	3.50E-02	25/485
	AMR	M & F	3.6E-02	7.70E-01	19/484
**(d) GIANT Body fat distribution**					
HIP	Admixed	M & F	9.6E-01	> 0.99	6/439
	EUR	M & F	9.6E-01	> 0.99	6/439
	Admixed	M	7.6E-01	> 0.99	9/438
	EUR	M	9.8E-01	> 0.99	5/438
	Admixed	F	8.5E-01	> 0.99	8/439
	EUR	F	8.5E-01	> 0.99	8/439
HIP adj. BMI	Admixed	M & F	7.6E-01	> 0.99	9/440
	EUR	M & F	9.6E-01	> 0.99	6/440
	Admixed	M	> 0.99	> 0.99	3/438
	EUR	M	9.8E-01	> 0.99	5/437
	Admixed	F	4.0E-01	> 0.99	12/439
	EUR	F	4.0E-01	> 0.99	12/439
WC	Admixed	M & F	9.2E-01	> 0.99	7/440
	EUR	M & F	9.2E-01	> 0.99	7/440
	Admixed	M	9.6E-01	> 0.99	6/438
	EUR	M	9.6E-01	> 0.99	6/438

	Admixed	F	5.2E-01	> 0.99	11/439
	EUR	F	6.5E-01	> 0.99	10/439
WC adj. BMI	Admixed	M & F	9.9E-01	> 0.99	5/440
	EUR	M & F	9.2E-01	> 0.99	7/440
	Admixed	M	7.6E-01	> 0.99	9/438
	EUR	M	8.5E-01	> 0.99	8/438
	Admixed	F	9.9E-01	> 0.99	5/439
	EUR	F	9.2E-01	> 0.99	7/439
WHR	Admixed	M & F	6.5E-01	> 0.99	10/440
	EUR	M & F	6.5E-01	> 0.99	10/440
	Admixed	M	1.3E-01	> 0.99	15/438
	EUR	M	2.0E-01	> 0.99	14/438
	Admixed	F	9.2E-01	> 0.99	7/439
	EUR	F	7.6E-01	> 0.99	9/439
WHR adj. BMI	Admixed	M & F	9.6E-01	> 0.99	6/440
	EUR	M & F	9.6E-01	> 0.99	6/440
	Admixed	M	> 0.99	> 0.99	4/438
	EUR	M	9.8E-01	> 0.99	5/437
	Admixed	F	9.2E-01	> 0.99	7/439
	EUR	F	9.2E-01	> 0.99	7/439
**(e) GIANT BMI & Height**					
GIANT BMI	EUR	M & F	2.0E-01	> 0.99	14/439
GIANT Height	EUR	M & F	2.9E-01	> 0.99	13/439
**(f) metabolic outcomes and diseases**					
T2D stage1	EUR	M & F	2.0E-01	> 0.99	14/440
T2D combined	EUR+SAS	M & F	6.5E-01	> 0.99	9/396
Fast Glucose	EUR	M & F	2.0E-01	> 0.99	14/441
Fast Insulin	EUR	M & F	8.6E-01	> 0.99	8/441
**(g) Inflammatory bowel disorder**					
IBD	EUR	M & F	7.7E-01	> 0.99	10/485
IBD Crohn's disease	EUR	M & F	5.5E-01	> 0.99	12/485
IBD Ulcerative colitis	EUR	M & F	9.2E-01	> 0.99	8/485
**(h) Longevity**					
Longevity > 90	EUR	M & F	9.6E-01	> 0.99	6/440

b) results for PFCTX

GWAS	POP	Gender	Hypergeometric P	FDR	#genes overlap /annotation
**(a) Neurodegenerative disorder**					
AMD	EUR+ASN	M & F	> 0.99	> 0.99	2/478
AMD Geographic Atrophy	EUR+ASN	M & F	8.5E-01	> 0.99	9/478
AMD Neovascular	EUR+ASN	M & F	9.9E-01	> 0.99	5/478
ALZ stage 1	EUR	M & F	4.9E-03	4.0E-02	22/482
ALZ	EUR	M & F	7.2E-01	> 0.99	1/50
Age at Huntington's disease motor onset	EUR	M&F	9.8E-01	> 0.99	6/478
Parkinson’s disease	EUR	M & F	9.5E-01	> 0.99	7/476
**(b) Neuropsychiatric disorder**					
ADHD	Admixed	M & F	> 0.99	> 0.99	4/439
BIP	Admixed	M & F	2.9E-01	8.3E-01	13/439
MDD	EUR	M & F	1.1E-01	5.3E-01	15/430
Schizophrenia	Admixed	M & F	9.9E-01	> 0.99	5/483
**(c) Cognitive functioning from HRS study**					
Cognitive slope	Admixed	M & F	1.2E-03	1.8E-02	24/485
	EUR	M & F	6.7E-01	> 0.99	11/485
	AFR	M & F	3.2E-01	8.8E-01	14/485
	AMR	M & F	1.6E-01	6.0E-01	16/485
Dementia	Admixed	M & F	> 0.99	> 0.99	4/485
	EUR	M & F	1.2E-03	1.8E-02	24/485
	AFR	M & F	2.6E-03	2.8E-02	23/485
	AMR	M & F	5.5E-01	> 0.99	12/484
**(d) GIANT Body fat distribution**					
HIP	Admixed	M & F	2.9E-01	8.3E-01	13/439
	EUR	M & F	2.0E-01	7.2E-01	14/439
	Admixed	M	2.9E-01	8.3E-01	13/438
	EUR	M	1.3E-01	5.3E-01	15/438
	Admixed	F	6.5E-01	> 0.99	10/439
	EUR	F	2.9E-01	8.3E-01	13/439
HIP adj. BMI	Admixed	M & F	3.3E-03	3.0E-02	21/440
	EUR	M & F	2.6E-02	1.7E-01	18/440
	Admixed	M	1.4E-03	1.8E-02	22/438
	EUR	M	6.4E-03	4.6E-02	20/437
	Admixed	F	2.9E-01	8.3E-01	13/439
	EUR	F	4.6E-02	2.7E-01	17/439
WC	Admixed	M & F	6.5E-01	> 0.99	10/440
	EUR	M & F	6.5E-01	> 0.99	10/440

	Admixed	M	> 0.99	> 0.99	4/438
	EUR	M	> 0.99	> 0.99	4/438
	Admixed	F	4.0E-01	9.4E-01	12/439
	EUR	F	4.0E-01	9.4E-01	12/439
WC adj. BMI	Admixed	M & F	1.3E-01	5.3E-01	15/440
	EUR	M & F	8.1E-02	4.4E-01	16/440
	Admixed	M	6.0E-04	1.8E-02	23/438
	EUR	M	2.5E-04	1.6E-02	24/438
	Admixed	F	7.6E-01	> 0.99	9/439
	EUR	F	6.5E-01	> 0.99	10/439
WHR	Admixed	M & F	> 0.99	> 0.99	4/440
	EUR	M & F	> 0.99	> 0.99	4/440
	Admixed	M	9.8E-01	> 0.99	5/438
	EUR	M	9.8E-01	> 0.99	5/438
	Admixed	F	7.6E-01	> 0.99	9/439
	EUR	F	6.5E-01	> 0.99	10/439
WHR adj. BMI	Admixed	M & F	9.9E-01	> 0.99	5/440
	EUR	M & F	9.9E-01	> 0.99	5/440
	Admixed	M	8.5E-01	> 0.99	8/438
	EUR	M	9.2E-01	> 0.99	7/437
	Admixed	F	8.5E-01	> 0.99	8/439
	EUR	F	9.2E-01	> 0.99	7/439
**(e) GIANT BMI & Height**					
GIANT BMI	EUR	M & F	9.2E-01	> 0.99	7/439
GIANT Height	EUR	M & F	1.3E-01	5.3E-01	15/439
(f) metabolic outcomes and diseases					
T2D stage1	EUR	M & F	8.6E-01	> 0.99	8/440
T2D combined	EUR+SAS	M & F	3.9E-01	9.4E-01	11/396
Fast Glucose	EUR	M & F	9.6E-01	> 0.99	6/441
Fast Insulin	EUR	M & F	7.7E-01	> 0.99	9/441
**(g) Inflammatory bowel disorder**					
IBD	EUR	M & F	> 0.99	> 0.99	4/485
IBD Crohn's disease	EUR	M & F	> 0.99	> 0.99	3/485
IBD Ulcerative colitis	EUR	M & F	> 0.99	> 0.99	2/485
**(h) Longevity**					
Longevity > 90	EUR	M & F	4.1E-01	9.4E-01	12/440

Supplementary Table 11: GWAS based overlap analysis between epigenetic age acceleration and complex phenotypes from 65 large scale GWAS. Overlap analysis between 65 GWAS of complex phenotypes with our GWAS of age acceleration from (a) all brain regions (ALL) and (b) in prefrontal cortex (PFCTX), respectively. Column 1 lists the names for the other study outcomes stratified by 8 categories (a to h). We used the following abbreviations for studies (listed following the order of rows): age-related macular degeneration (AMD), Alzheimer's disease (ALZ), attention deficit and hyperactivity disorder (ADHD), bipolar disorder (BIP), major depression disorder (MDD), body mass index (BMI), waist circumference (WC), waist to hip ratio (WHR), type 2 diabetes (T2D), and inflammatory bowel disorder (IBD). Column 2 lists genetic ancestry with abbreviations listed in the following: Asian (ASN), African (AFR), Hispanic (AMR), European (EUR), and SAN (southern Asians) ancestry. The third to fifth columns list one-sided hypergeometric *P* values, Benjamin-Hochberg false discovery rate and the proportion of trait related genes that also relate to brain age acceleration respectively, stratified by the overlap results with ALL and PFCTX.

Band	#Markers	SNP	Nearby Gene	Position (bp)	Minor/ Major alleles	MAF	EUR MAF	Corr. (SE)	Meta *P*	I_2 (%) (*P*)
10q26	3	rs10632388	*TACC2*	123750056	GCC/ G	0.37	0.41	-0.22 (0.04)	2.7E-08	32.2 (0.2)
12p13.31	10	rs7305935	*CLEC4E-AICDA*	8706474	T/ A	0.47	0.49	-0.22 (0.04)	2.6E-08	15.8 (0.3)

Position bp based on Hg19 assembly;
#Markers=number of markers with association $P < 5.0x10^{-8}$;
Corr.= Correlation with respect to minor allele;
MAF=mean of minor allele frequency estimates across studies weighted by study sample sizes;
EUR MAF=minor allele frequency calculated using 1000 genome individuals with ancestry of European (released in December 2013).

Supplementary Table 12: SNPs significantly associated with proportion of neurons in PFCTX. We present the loci with SNP associations at $5.0x10^{-8}$ and report the most significant SNP within each locus. Fixed effects meta-analysis was used to estimate the correlation coefficient and standard error ("Corr. (SE)") between the minor allele and the proportion of neurons in PFCTX. The corresponding meta-analysis P values can be found in the column "Meta P". The brain regions in prefrontal cortex (PFCTX) include dorsolateral prefrontal cortex.

GWAS	POP	Gender	Hypergeometric P	FDR	#genes overlap /annotation
**(a) Neurodegenerative disorder**					
AMD	EUR+ASN	M & F	3.8E-06	2.1E-05	30/478
AMD Geographic Atrophy	EUR+ASN	M & F	1.5E-07	1.0E-06	33/478
AMD Neovascular	EUR+ASN	M & F	1.4E-12	3.1E-11	42/478
ALZ stage 1	EUR	M & F	9.2E-01	> 0.99	8/482
ALZ	EUR	M & F	> 0.99	> 0.99	0/50
Age at Huntington's disease motor onset	EUR	M&F	9.1E-02	1.5E-01	14/478
Parkinson's disease	EUR	M & F	8.6E-03	2.1E-02	21/476
**(b) Neuropsychiatric disorder**					
ADHD	Admixed	M & F	7.6E-01	8.7E-01	9/439
BIP	Admixed	M & F	5.2E-01	6.4E-01	11/439
MDD	EUR	M & F	6.2E-01	7.2E-01	10/430
Schizophrenia	Admixed	M & F	1.6E-09	1.7E-08	37/483
**(c) Cognitive functioning from HRS study**					
Cognitive slope	Admixed	M & F	2.3E-01	3.2E-01	15/485
	EUR	M & F	1.0E-01	1.6E-01	17/485
	AFR	M & F	6.1E-02	1.1E-01	18/485
	AMR	M & F	5.5E-01	6.5E-01	12/485
Dementia	Admixed	M & F	5.3E-04	1.7E-03	25/485
	EUR	M & F	3.6E-02	7.2E-02	19/485
	AFR	M & F	2.3E-01	3.2E-01	15/485
	AMR	M & F	9.9E-02	1.6E-01	17/484
**(d) GIANT Body fat distribution**					
HIP	Admixed	M & F	5.2E-01	6.4E-01	11/439
	EUR	M & F	1.3E-02	3.1E-02	19/439
	Admixed	M	5.2E-07	3.1E-06	30/438
	EUR	M	1.6E-07	1.1E-06	31/438
	Admixed	F	1.3E-02	3.1E-02	19/439
	EUR	F	1.3E-01	1.9E-01	15/439
HIP adj. BMI	Admixed	M & F	1.1E-04	3.8E-04	25/440
	EUR	M & F	1.5E-03	4.2E-03	22/440
	Admixed	M	4.1E-09	3.8E-08	34/438
	EUR	M	1.1E-09	1.4E-08	35/437
	Admixed	F	5.2E-01	6.4E-01	11/439
	EUR	F	5.2E-01	6.4E-01	11/439
WC	Admixed	M & F	5.3E-01	6.4E-01	11/440
	EUR	M & F	8.6E-01	9.6E-01	8/440
	Admixed	M	2.5E-02	5.5E-02	18/438

	EUR	M	4.0E-01	5.4E-01	12/438
	Admixed	F	2.6E-02	5.5E-02	18/439
	EUR	F	4.6E-02	8.8E-02	17/439
WC adj. BMI	Admixed	M & F	2.6E-02	5.5E-02	18/440
	EUR	M & F	3.3E-03	8.8E-03	21/440
	Admixed	M	6.0E-04	1.9E-03	23/438
	EUR	M	9.9E-05	3.8E-04	25/438
	Admixed	F	1.3E-01	1.9E-01	15/439
	EUR	F	4.6E-02	8.8E-02	17/439
WHR	Admixed	M & F	2.0E-01	2.9E-01	14/440
	EUR	M & F	4.7E-02	8.8E-02	17/440
	Admixed	M	1.4E-03	4.1E-03	22/438
	EUR	M	6.5E-03	1.7E-02	20/438
	Admixed	F	> 0.99	> 0.99	4/439
	EUR	F	> 0.99	> 0.99	2/439
WHR adj. BMI	Admixed	M & F	1.1E-04	3.8E-04	25/440
	EUR	M & F	4.0E-05	1.8E-04	26/440
	Admixed	M	4.8E-06	2.2E-05	28/438
	EUR	M	4.6E-06	2.2E-05	28/437
	Admixed	F	1.3E-01	1.9E-01	15/439
	EUR	F	8.0E-02	1.4E-01	16/439
**(e) GIANT BMI & Height**					
GIANT BMI	EUR	M & F	9.6E-01	> 0.99	6/439
GIANT Height	EUR	M & F	5.2E-01	6.4E-01	11/439
**(f) metabolic outcomes and diseases**					
T2D stage1	EUR	M & F	2.8E-13	9.1E-12	41/440
T2D combined	EUR+SAS	M & F	1.4E-04	4.9E-04	23/396
Fast Glucose	EUR	M & F	9.9E-01	> 0.99	5/441
Fast Insulin	EUR	M & F	8.2E-02	1.4E-01	16/441
**(g) Inflammatory bowel disorder**					
IBD	EUR	M & F	9.2E-12	1.5E-10	41/485
IBD Crohn's disease	EUR	M & F	6.0E-09	4.9E-08	36/485
IBD Ulcerative colitis	EUR	M & F	<1.0E-20	<1.0E-20	53/485
**(h) Longevity**					
Longevity > 90	EUR	M & F	9.9E-01	> 0.99	5/440

Supplementary Table 13: GWAS based overlap analysis between the proportion of neurons and complex phenotypes from 65 large scale GWAS. Overlap analysis results with a total of 65 GWAS results for age-related outcomes using our GWAS results from neuronal proportion (NP) in PFCTX. Column 1 lists the names for age-related study outcomes stratified by 8 categories (a to h). We used the following abbreviations for studies (listed following the order of rows) : age-related macular degeneration (AMD), Alzheimer's disease, attention deficit and hyperactivity disorder (ADHD), bipolar disorder (BIP), major depression disorder (MDD), body mass index (BMI), waist circumference (WC), waist to hip ratio (WHR), type 2 diabetes (T2D), and inflammatory bowel disorder (IBD). Column 2 lists genetic ancestry with abbreviations listed in the following: Asians (ASN), Africans (AFR), Americans (AMR), Europeans (EUR), and southern Asians (SAS). The third to fifth columns list one-sided hypergeometric *P* values, Benjamin-Hochberg false discovery rate and the proportion of trait related genes that also relate to NP.

Outcome	Exposure	#SNPs	Pleiotropic effect β_{0E} (SE)	Pleiotropic effect P	Causal effect β_E (SE)	Causal effect P
Epigenetic age acceleration	HIP adj. BMI	39	-0.008 (0.049)	0.9	-1.168 (1.198)	0.3
	WC adj. BMI	29	-0.005 (0.042)	0.9	-1.883 (1.224)	0.2
Proportion of neurons	HIP adj. BMI	39	0.021(0.045)	0.6	-0.088 (1.099)	0.9
	WC adj. BMI	29	-0.016 (0.048)	0.7	1.862 (1.397)	0.2

HIP adj. BMI=hip circumference adjusted for body mass index; WC=waist circumference.

Supplementary Table 14: MR-Egger regression analysis. The table presents the results from a total of four MR-Egger regression models, stratified by exposure and outcome variables. The third column lists number of uncorrelated GWAS SNPs as instrumental variables. The fourth macro column lists the estimate of average pleiotropic effect from SNPs directly affecting an outcome variable. A significant P value indicates directional pleiotropy that pleiotropic effects over the SNPs are not balanced about the null. The fifth macro column lists the bias-reduced estimate of causal effect (exposure -> outcome) that already removes the bias due to pleiotropic effect.

Body fat composition	Platform	β	SE	P
**HIP adj. BMI**	Illumina	0.08	0.15	0.62
	Affymetrix	0.67	0.38	0.09
	Combined	0.16	0.14	0.27
**WC adj. BMI**	Illumina	0.13	0.15	0.37
	Affymetrix	-0.35	0.36	0.35
	Combined	0.06	0.14	0.63

Supplementary Table 15: Causal effect of body fat distribution traits on epigenetic age acceleration in PFCTX. The table presents the effect of body fat compositions on the age acceleration for stratified and combined results, listing effect sizes (β), standard errors (SE) and P values**.** We conducted Mendelian randomization based approach to examine the causal effect of body fat composition on epigenetic age acceleration in PFCTX using all the individuals from our studies 6 &7. We generated a genetic measure of hip circumference using polygenetic risk score (PRS). The PRS variable is a sum of risk alleles weighted by effect sizes using the GWAS results of hip adjusted for body mass index (HIP adj. BMI) based on 93,965 *males* of European ancestry in the GIANT consortium[5]. Thus, the generated hip measure was independent with BMI and reflects sex-specific effect. The PRS variable was constructed based on all genome-wide association results that we used independent SNPs selected by LD-based clumping procedure. We implemented the analysis in PLINK with a threshold of r^2 set at 0.2 and a threshold of *P* value set at 1, in a window size of 250kb. The qualities of SNPs were controlled by info measure ≥ 0.4 and MAF ≥ 0.10 prior the clumping procedure. The LD estimation was calculated using the 379 individuals with European ancestry from 1000 Genome reference panel (released in December 2013). A regression analysis of intrinsic epigenetic age acceleration (adjusted for gender, disease

status and proportion of neurons) on hip adj. BMI was performed. In an analogous manner, we conducted the same regression analysis using the predictor based on the PGS of waist circumference adjusted for BMI (WC adj. BMI).

As noted, the study individuals were collected from Rush Alzheimer's Disease Center with genotyping performed on Affymetrix SNP Array 6.0 or Illumina HumanOmniExpress platform. Imputation was performed on the individuals stratified by platform type. We therefore conducted the analysis on the individuals stratified by platform type then combined the results via fix-effects models weighted by inverse variance.

Body fat composition	Platform	β	SE	P
**HIP adj. BMI**	Illumina	0.000	0.003	0.9
	Affymetrix	-0.002	0.007	0.8
	Combined	0.0003	0.002	0.9
**WC adj. BMI**	Illumina	3.82E-05	0.002	1.0
	Affymetrix	-0.004	0.007	0.5
	Combined	0.000	0.002	0.8

Supplementary Table 16: Causal effect of body fat distribution traits on proportion of neurons in PFCTX. We conducted the same Mendelian randomization based approach (described in Supplementary Table 15) to examine the causal effect of body fat composition on the measure of brain aging based on the proportion of neurons adjusted for chronological age.

Genetic Ancestry	N	Male	Age at Wave 3	Age at Wave 9	College[a]	Cognitive slope	Dementia (case/control)
ALL	12452	41%	62 (7.4) [50,90]	70 (9.5) [50,102]	22%	1.04 (0.68) [-7.13, 14]	690 (9%)/7229 (91%)
EUR	9564	42%	62 (7.5) [50,90]	71 (9.5) [50,100]	25%	1.05 (0.64) [-6.33, 12.2]	348(5%)/6116(95%)
AFR	1553	36%	62 (7.1) [50,89]	69 (9.2) [50,102]	12%	1.04 (0.82) [-7.13, 14]	212 (27%)/578 (73%)
AMR	1035	40%	61 (6.7) [50,84]	67 (9.2) [52,96]	7%	0.96 (0.73) [-2.67, 5.1]	108 (21%)/409(79%)
ASN	91	32%	61 (6.3) [50,80]	69 (9.6) [54,92]	47%	1.09 (0.92) [-0.65, 6.38]	2(4%)/45(96%)
Others	209	32%	61 (7.2) [50,79]	70 (9.2) [54,92]	14%	0.99 (0.81) [-4.98, 3.47]	20(20%)/81(80%)

Quantitative variables are presented in the format of mean(sd) [range].
Abbreviations: ALL=all genetic ancestry; EUR=Europeans; AFR=Africans; AMR=Americas; ASN=Asians.
[a]College and above.

Supplementary Table 17: Characteristics of the participants of the HRS.

We retrieved the HRS longitudinal data during 1996 to 2010 (Waves 3 to 10). The characters of study individuals are listed including the distributions of three cognitive functioning measurements used for GWAS analysis. In particular, the cognitive slope is a measure that assesses the change in cognitive age given the change in chronological age over the fourteen years (1996-2010). The binary dementia outcome combines the assessments for dementia at Waves 8 and 9 as listed in **Supplementary note 2**.

Trait	Genetic ancestry	λ_{GC}	No. SNPs	No. PCs	Age	Gender	College[a]
**Cognitive slope**	Admixed	0.99	4754646	4	N	Y	Y
	EUR	1.00	5798029	2	N	Y	Y
	AFR	1.00	7982837	2	N	Y	Y
	AMR	0.99	5888282	2	N	Y	Y
**Dementia**	Admixed	1.00	5474123	4	Y	Y	Y
	EUR	1.01	5798126	2	Y	Y	Y
	AFR	1.00	7599097	2	Y	Y	Y
	AMR	1.01	5819731	2	Y	Y	Y

[a]Education for college and above.

Supplementary Table 18: Genomic inflation estimates for cognitive traits in the HRS. Genomic inflation estimates $\boldsymbol{\lambda_{GC}}$ for cognitive functioning traits in different ethnic strata of the Health and Retirement Study. Column 2 reports the group of individuals with "Admixed" denoting all samples combined irrespective of race/ethnicity. The GWAS was conducted using the number of SNPs (column "No. SNPs" with common variants (MAF ≥ 5%)). To protect against spurious association results, the analysis was adjusted for principal components (PCs), gender, and education (college or above).

Supplementary Note 1: Description of datasets

Individuals of European ancestry from each study were included in our GWA meta-analysis. Genetic ancestry was identified in PLINK or EIGENSTRAT[6] for each study, respectively. Here we describe resources of each study and disease status of study individuals.

Study 1: These brain tissues are part of the study samples used in a study for Alzheimer's disease[7], archived in the MRC London Brain bank for Neurodegenerative Disease. We obtained genotyping and DNA methylation data for 63 individuals, including 38 diagnosed with Alzheimer's disease. DNA methylation data are available for free public download (Table 1). Individuals with missing age acceleration estimates were removed from the GWAS, yielding 63 subjects remained for analysis.

Study 2: All of the 148 individuals were neurologically normal[8]. The SNP data was archived in dbGAP, http://www.ncbi.nlm.nih.gov/gap, with accession: phs000249.v1.p1. The GWAS was conducted in 142 individuals with age acceleration estimates for brain tissues. Gene expression and DNA methylation data are available for free public download (Table 1).

Study 3: The 153 individuals were used for a case control (121 cases/32 controls) study for psychiatric disorders[9]. The SNP data was downloaded from Stanley Medical Research Institute https://www.stanleygenomics.org/stanley/standard/studyDetail.jsp?study_id=20. Assays of genotyping and DNA methylation profiled in 147 individuals were used in our study. Gene expression and DNA methylation data are available for free public download (Table 1).

Study 4: We obtained genotyping and DNA methylation data for 44 individuals from a case control study for Schizophrenia in [10]. Of those, 37 individuals (19 controls/ 17 cases) verified of European ancestry were used in our study. DNA methylation data are available for free public download (Table 1).

Study 5: All subjects (n=232) were neurologically normal[11]. The SNP data was archived in dbGAP, http://www.ncbi.nlm.nih.gov/gap, with accession: phs000249.v2.p1. We excluded 3 individuals genotyped in a different platform, 6 identified as genetic outliers, 13 who failed the QC in DNA methylation arrays in terms of ambiguous gender, DNAmAge estimates outliers (deviating from chronological age greater than 40 years). In addition, ambiguous gender was indicated by the predicted gender via the algorithm of DNAm age calculation (see Methods). This yields a total of 209 distinct individuals available for GWAS study with DNA methylation measured in cerebellum or frontal cortex region. Gene expression and DNA methylation data are available for free public download (Table 1).

Study 6: We obtained genotyping data for 923 individuals of European ancestry from the Religious Order Study (ROS) for a case control study for Alzheimer's disease[12-14]. DNA methylation data profiled from dorsolateral prefrontal cortex region were collected from deceased participants[15]. After applying QC in DNA methylation array, 303 individuals (161 controls/142 cases) were available for GWAS study.

Study 7: We obtained genotyping data for 1167 individuals of European ancestry from the Rush Memory and Aging Project (MAP) for a case control study for Alzheimer's disease[12-14]. DNA methylation data profiled from dorsolateral prefrontal cortex region were collected from deceased participants[15]. After applying QC in DNA methylation array, 262 individuals (149 controls/ 113 cases) were available for GWAS study.

Supplementary Note 2: Age-related studies used in our hypergeometric based overlap analysis

Below, we briefly describe large scale GWAS studies that were cross referenced to our GWAS study of epigenetic age acceleration. The GWAS results are from previously published articles, except cognitive functioning traits using the Health and Retirement Study (HRS) data. We performed two GWAS for one cognitive functioning trait: one from our previous study[3] and the other one conducted in this study, as described in more details below.

Age-related macular degeneration (AMD)

A large-scale GWAS meta-analysis was performed in the study including >17,100 advanced AMD cases and >60,000 controls of European and Asian ancestry in the analysis[16], conducted by AMD Gene Consortium Study of Age Related Macular Degeneration. We downloaded three summary results of ~ 2.4 million markers for studying advanced AMD versus control subjects, including two AMD subtypes: geographic atrophy and neovascular, and AMD with any of subtype, from http://www.sph.umich.edu/csg/abecasis/public/amdgene2012/.

Alzheimer's disease

The IGAP consortium performed a GWAS meta-analysis on 74,046 individuals of European ancestry[17]. We downloaded the summary results of GWAS from http://www.pasteur-

lille.fr/en/recherche/u744/igap/igap_download.php. Two sets of association results are available. The first set includes the GWAS results of meta-analysis based upon 17,008 Alzheimer's disease cases and 37,154 controls at stage 1 analysis. A total of 11,632 SNPs exhibited moderate evidence of association ($P < 1.0 \times 10^{-3}$) at stage 1. The second set includes the *P* values of the 11,632 SNPs from the final meta-analysis that combined stages 1 &2 results. We used both association results for our overlap analysis.

DIAGRAM Type 2 diabetes

The DIAbetes Genetics Replication and Meta-analysis (DIAGRAM) consortium performed a GWAS meta-analysis for type 2 diabetes (T2D) in [18]. The first set includes the GWAS results of meta-analysis based upon 22,669 cases and 58,119 controls of European decent at stage 1 analysis. The stage 2 analysis was performed on 1,178 cases and 2,472 controls of Pakistani decent. A subsequent meta-analysis was performed to combine both stages 1 and 2 results. Stage 1 and the combined meta-analyses results are available for download from http://diagram-consortium.org/downloads.html. We used both association results for our overlap analysis.

GIANT body fat distribution

The genetic investigation of anthropometric trait (GIANT) consortium performed a GWAS meta-analysis for body fat distribution traits on 22,459 individuals of European (the majority), East Asian, South Asia, and African American ancestry[5]. Three body fat distributions traits were analyzed including hip, waist circumference and waist-to-hip ratio, each involved 12 GWAS results stratified by gender (males, females and both) cross genetic ancestry (EUR or admixed) cross adjusted for body mass index (BMI, yes or no). This yielded a total of 36 GWAS analysis available for overlap analysis. We downloaded the summary results of GWAS from https://www.broadinstitute.org/collaboration/giant/index.php/GIANT_consortium_data_files.

GIANT BMI and height

The GIANT consortium performed a GWAS meta-analysis for BMI and height traits on ~170,000 individuals of Europe ancestry[19]. The majority individuals (*N*=133,454) were used for discovery phase and the others were used for replication. GWAS results are for only available for discovery phase that can be downloaded from,

https://www.broadinstitute.org/collaboration/giant/index.php/GIANT_consortium_data_files.

IIBDGC Inflammatory bowel disease

The International Inflammatory Bowel Disease Genetics Consortium (IIBDGC) performed a large-scale GWAS meta-analysis for studying inflammatory bowel disease (IBD) on 86,640 European individuals at discovery phase and 9,846 individuals of East Asian, Indian or Iranian decent at replication phase[20]. Three traits were analyzed including two forms of IBD: ulcerative colitis and Crohn's disease, as well as IBD in either form. As the results for replication analysis performed by Bayesian trans-ancestry meta-analysis that reports Bays factor rather than *P* values, we only conducted the overlap analysis using the GWAS results at discovery phase for the three IBD forms, respectively. At discovery phase, 34652 individuals were available for genome-wide genotype data and the other 51988 individuals were only available for Immnuochip genotype data. The GWAS results using the genotypes of 34,652 individuals can be downloaded from http://www.ibdgenetics.org/.

The Health and Retirement Study (HRS)

The Health and Retirement Study (1992-2012) is a longitudinal panel study of a representative sample of Americans over age 50 (and their spouses), collected every two years (**Supplementary Table 8**). We downloaded study materials, genotype (including imputed markers) and phenotype data of ~ 12,500 individuals from dbGAP with access: phs000428.v1.p1. To evaluate the genetic

variants overlapping between epigenetic age acceleration and cognitive functioning, GWAS was performed on genotyped and imputed markers for two relative traits: (1) cognitive aging slope, (2) an overall binary dementia status that combined the assessments for dementia at wave 8 (diagnosed in year 2006) and wave 9 (diagnosed in year 2008), respectively. Specifically, the dementia assessments yielded a three category variable: normal, mild cognitive impairment or cognitive impairment not demented (CIND), and dementia, at waves 8 and 9, respectively. To combine the assessments of the two waves, we assigned individuals as dementia if he was allocated to dementia in either wave, assigned individuals as normal if he was allocated to normal in both waves, and missing for the others (see **Supplementary Table 17**). The procedures of GWAS and the definitions of the traits are descried below.

GWAS in the HRS

We performed association analysis on genotyped and imputed SNPs with common variants. Genotyping data were performed on Illumina's Human Omni2.5-Quad (Omni2.5) platform and imputed data were computed with IMPUTE2. Quality control of SNPs was guided by HWE $P >$ 1.0×10^{-6} along with info measure > 0.4 for imputed markers and thresholded genotypes set at 0.9 for imputed genotypes. In addition, we required minimum number of samples at 200 per marker. Since the HRS study is comprised of different racial/ethnic groups, we either restricted the GWAS analysis to a given ethnic group or used principal components (from an identity by state analysis) in multivariate regression models. Post association analysis, we pruned out the SNPs associated with large effect sizes guided by odds ratios (> 3 or < 1/3). More details for model framework and assessments for GWAS results can be found in **Supplementary Table 9**.

Cognitive slope for measuring cognitive decline

The cognitive slope defines the change in cognitive age given the change in chronological age over fourteen years (1996-2010). To calculate the slope, we determined cognitive ages for each participant at each wave, based on data from wave three (1996) through wave ten (2010) for four measures of cognitive functioning—delayed recall, immediate recall, serial 7s, and backwards counting. For all waves, the score for immediate and delayed recall were both based on the number of words correctly recalled out of ten possible. The delayed recall was assessed approximately five minutes after the word list was read, between which other tasks were performed. The scores for serial 7s ranged from 0-5. Respondents were asked to continuously subtract by 7, starting from 100. Points were awarded for correct responses for a total of five subtractions. For backwards counting scores, respondents were asked to count backwards, 10 consecutive times, beginning with the number 20. A total of two points were allotted for two attempts at backwards counting—participants received a zero if they were incorrect on both tries; a 1 if they were incorrect on the first try, but correct on the second; and a 2 if they correctly completed the task on the first try.

A cognitive age was calculated for each participant at each wave. The method for calculating cognitive age was based on an algorithm proposed by Klemera & Doubal[21] that is typically used for calculating biological ages from biomarker data[22]. This method has been validated using both real and simulated data. Cognitive age estimates combine information from equations of chronological age regressed on each of the cognitive functioning markers. The equation for calculating cognitive age is:

$$\text{Cognitive age} = \frac{\sum_{j=1}^{m}(x_{ji}-q_j)\frac{k_j}{s_j^2}+\frac{CA_i}{s_{BA}^2}}{\sum_{j=1}^{m}\left(\frac{k_j}{s_j}\right)^2+\frac{1}{s_{BA}^2}}$$

Where, k_j and q_j are the slope and intercept, respectively, for the regression of chronological age and each cognitive measure, x_{ji} is the value of cognitive measure j for participant i, s_j is the root mean squared error of chronological age regressed on the jth cognitive measure, and CA_i is chronological age for participant i. Additionally s_{BA}^2, the variance of the random variable, takes into account the variability in the first half of the equation, the mean variance of the cognitive measures that is explained by chronological age, and the range of chronological age. Overall, the mean cognitive age of a population should equal the mean chronological age of the population[21]. Once cognitive ages were determined for each participant at each wave we calculated the slope for the change in cognitive age given the change in chronological age over eight waves (1996-2010).

Dementia assessment at each wave

The HRS participants with ages 70 and over (n=6,412) were classified into normal, CIND, and dementia groups. Predicted dementia status also relied on the four cognitive functioning variables that were used to estimate cognitive aging—delayed recall, immediate recall, serial 7s, and backwards counting. However, dementia status also took into account proxy responses for participants who were unable to complete the cognitive battery. For those who were able to respond, scores across the four variables were summed. Participants with total score ranging between 12 and 27 were categorized as having normal cognitive functioning; those with scores between 7 and 11 were categorized as having CIND, and those with scores of six or less were categorized as having dementia.

Participants whose status relied on proxy respondents were categorized in accordance with the method proposed by Langa et al.[23]. For these participants, cognitive status was based on the sum of scores from three measures taken from memory assessments by a proxy (0=excellent, 1=very good, 2=good, 3=fair, 4=poor), the participant's total number of IADL limitations, and the

interviewer's assessment of whether the participant had difficulty completing the cognitive battery due to cognitive limitations (0-2 indicating, no limitation, some limitation, and limitation prevents completion, respectively). After these three measures were summed, participants with total scores between 0 and 2 were categorized as having normal cognitive functioning, those with scores between 3 and 5 were categorized as having CIND, and those with scores of 6 or more were categorized as demented.

Age at Huntington's Disease (HD) motor onset

The GWAS meta-analysis was performed for 4,082 HD subjects collected from the Massachusetts HD Center Without Wall (MAHDC) and the Genetics Modifies of HD (GeM-HD) [24]. All the HD subjects are with European ancestry. Age at onsite motor was adjusted for the influence of *HTT* CAG repeat then the residual values were used as phenotypes for quantitative association analysis. We downloaded the summary data from the Genetic Modifies of Motor Onset Age (GeM-MOA, http://chgr.partners.org/cgi-bin/gem.moa/gem.moa.py), with access granted by the GeM-MOA consortium.

Longevity study

The GWAS meta-analyses study was performed for 98,066 individuals of European ancestry[25], including discovery, replication and joint analyses. The summary results at discovery phase analysis can be downloaded from http://hmg.oxfordjournals.org/content/23/16/4420/suppl/DC1. The results include two types of association *P* values with respect to (1) individuals with longevity > 85 versus < 65 and (2) individuals with longevity > 90 versus < 65. We only report the results for comparison (2), i.e. longevity >90 versus <65. The results for the first comparison were similar.

MAGIC Glycemic measure

The Meta-Analyses of Glucose and Insulin-related traits (MAGIC) consortium performed a GWAS meta-analysis on (a) 58,074 non-diabetic individuals for fasting glucose trait and (b) 51,750 non-diabetic individuals for fasting insulin trait, respectively. All study individuals are of European descent. BMI variation was accounted for GWAS in both traits. Data on glycemic traits have been contributed by MAGIC investigators and have been downloaded from www.magicinvestigators.org and the relevant article can be found in [26].

Parkinson's disease study

A two stage genome wide association study for Parkinson's disease was conducted in 13,625 individuals of European ancestry[27]. For our analysis, we utilized the results of 463187 SNPs at stage 1 phase released in dbGAP with accession: pha002868.1 downloaded from, http://www.ncbi.nlm.nih.gov/projects/gap/cgibin/analysis.cgi?study_id=phs000501.v1.p1&pha=2868.

PGC Attention-deficit/ hyperactivity disorder

The Psychiatric Genomics Consortium (PGC) performed a GWAS meta-analysis for studying attention-deficit/hyperactivity disorder (ADHD)[28]. The study individuals were across pedigree data (2,064 trios) and case-control (896 cases and 2,455 controls) data sets, with the majority of European ancestry. The GWAS results can be downloaded from http://www.med.unc.edu/pgc/downloads.

PGC bipolar disorder

The PGC performed a combined GWAS meta-analysis for studying bipolar disorder[29]. The study comprised 7,481 cases and 9,250 controls. Ancestry of individuals identified by multi-dimensional

scaling (MDS) analysis was used to identify ancestry of study individuals and was used to correct population stratification in association analysis. The GWAS results can be downloaded from http://www.med.unc.edu/pgc/downloads.

PGC major depression disorder

The PGC performed a combined GWAS meta-analysis for studying major depression disorder[30]. Association analysis was performed on discovery phased (9,240 cases and 9,519 controls of European ancestry), replication phase (6,783 cases and 50,695 controls), and mega-analysis (9,238 major depression disorder cases/8,039 controls, and 6,998 bipolar disorder cases and 7775 controls) for cross-disorder trait, the last two only involved a small number of SNPs ($m \leq 819$). Only the GWAS results for discovery phase are available for download from http://www.med.unc.edu/pgc/downloads.

PGC Schizophrenia

The PGC performed a multi-stage large-scale GWAS meta-analysis for studying schizophrenia up to 36,989 cases and 113,075 controls[31]. The majority of individuals are of European ancestry. A primary GWAS meta-analysis was performed on 49 case-control samples (46 of European and 3 of East Asian ancestry, 34,241 cases and 45,604 controls) and 3 family-based samples of European ancestry (1,235 parent affected-offspring trios). The overlap analysis was based on the primary results downloaded from http://www.med.unc.edu/pgc/downloads.

Supplementary References

1. Langfelder, P. & Horvath, S. WGCNA: an R package for weighted correlation network analysis. *BMC Bioinformatics* **9**, 559 (2008).
2. Roadmap Epigenomics, C. *et al.* Integrative analysis of 111 reference human epigenomes. *Nature* **518**, 317-30 (2015).
3. Lu, A.T. *et al.* Genetic variants near MLST8 and DHX57 affect the epigenetic age of the cerebellum. *Nat Commun* **7**, 10561 (2016).
4. Consortium, G.T. Human genomics. The Genotype-Tissue Expression (GTEx) pilot analysis: multitissue gene regulation in humans. *Science* **348**, 648-60 (2015).
5. Shungin, D. *et al.* New genetic loci link adipose and insulin biology to body fat distribution. *Nature* **518**, 187-96 (2015).
6. Price, A.L. *et al.* Principal components analysis corrects for stratification in genome-wide association studies. *Nat Genet* **38**, 904-9 (2006).
7. Lunnon, K. *et al.* Methylomic profiling implicates cortical deregulation of ANK1 in Alzheimer's disease. *Nat Neurosci* **17**, 1164-70 (2014).
8. Gibbs, J.R. *et al.* Abundant quantitative trait loci exist for DNA methylation and gene expression in human brain. *PLoS Genet* **6**, e1000952 (2010).
9. Zhang, D. *et al.* Genetic control of individual differences in gene-specific methylation in human brain. *Am J Hum Genet* **86**, 411-9 (2010).
10. Pidsley, R. *et al.* Methylomic profiling of human brain tissue supports a neurodevelopmental origin for schizophrenia. *Genome Biol* **15**, 483 (2014).
11. Hernandez, D.G. *et al.* Distinct DNA methylation changes highly correlated with chronological age in the human brain. *Hum Mol Genet* **20**, 1164-72 (2011).
12. Bennett, D.A., Schneider, J.A., Arvanitakis, Z. & Wilson, R.S. Overview and findings from the religious orders study. *Curr Alzheimer Res* **9**, 628-45 (2012).
13. Bennett, D.A. *et al.* Overview and findings from the rush Memory and Aging Project. *Curr Alzheimer Res* **9**, 646-63 (2012).
14. Shulman, J.M. *et al.* Genetic susceptibility for Alzheimer disease neuritic plaque pathology. *JAMA Neurol* **70**, 1150-7 (2013).
15. De Jager, P.L. *et al.* Alzheimer's disease: early alterations in brain DNA methylation at ANK1, BIN1, RHBDF2 and other loci. *Nat Neurosci* **17**, 1156-63 (2014).
16. Fritsche, L.G. *et al.* Seven new loci associated with age-related macular degeneration. *Nat Genet* **45**, 433-9, 439e1-2 (2013).
17. Lambert, J.C. *et al.* Meta-analysis of 74,046 individuals identifies 11 new susceptibility loci for Alzheimer's disease. *Nat Genet* **45**, 1452-8 (2013).
18. Morris, A.P. *et al.* Large-scale association analysis provides insights into the genetic architecture and pathophysiology of type 2 diabetes. *Nat Genet* **44**, 981-90 (2012).
19. Yang, J. *et al.* FTO genotype is associated with phenotypic variability of body mass index. *Nature* **490**, 267-72 (2012).
20. Liu, J.Z. *et al.* Association analyses identify 38 susceptibility loci for inflammatory bowel disease and highlight shared genetic risk across populations. *Nat Genet* **47**, 979-86 (2015).
21. Klemera, P. & Doubal, S. A new approach to the concept and computation of biological age. *Mech Ageing Dev* **127**, 240-8 (2006).

22. Levine, M.E. Modeling the rate of senescence: can estimated biological age predict mortality more accurately than chronological age? *J Gerontol A Biol Sci Med Sci* **68**, 667-74 (2013).
23. Langa, K.M. *et al.* The Aging, Demographics, and Memory Study: study design and methods. *Neuroepidemiology* **25**, 181-91 (2005).
24. Genetic Modifiers of Huntington's Disease, C. Identification of Genetic Factors that Modify Clinical Onset of Huntington's Disease. *Cell* **162**, 516-26 (2015).
25. Deelen, J. *et al.* Genome-wide association meta-analysis of human longevity identifies a novel locus conferring survival beyond 90 years of age. *Hum Mol Genet* **23**, 4420-32 (2014).
26. Manning, A.K. *et al.* A genome-wide approach accounting for body mass index identifies genetic variants influencing fasting glycemic traits and insulin resistance. *Nat Genet* **44**, 659-69 (2012).
27. Simon-Sanchez, J. *et al.* Genome-wide association study reveals genetic risk underlying Parkinson's disease. *Nat Genet* **41**, 1308-12 (2009).
28. Neale, B.M. *et al.* Meta-analysis of genome-wide association studies of attention-deficit/hyperactivity disorder. *J Am Acad Child Adolesc Psychiatry* **49**, 884-97 (2010).
29. Psychiatric, G.C.B.D.W.G. Large-scale genome-wide association analysis of bipolar disorder identifies a new susceptibility locus near ODZ4. *Nat Genet* **43**, 977-83 (2011).
30. Major Depressive Disorder Working Group of the Psychiatric, G.C. *et al.* A mega-analysis of genome-wide association studies for major depressive disorder. *Mol Psychiatry* **18**, 497-511 (2013).
31. Schizophrenia Working Group of the Psychiatric Genomics, C. Biological insights from 108 schizophrenia-associated genetic loci. *Nature* **511**, 421-7 (2014).
